# Synthesis and Biophysical Characterization of Fingolimod Derivatives as Cardiac Troponin Antagonists

**DOI:** 10.1021/acsmedchemlett.3c00511

**Published:** 2024-02-07

**Authors:** Laszlo Kondacs, Priyanka Parijat, Alexander J. A. Cobb, Thomas Kampourakis

**Affiliations:** † Department of Chemistry, King’s College London, Britannia House, London SE1 1DB, United Kingdom; ‡ Randall Centre for Cell and Molecular Biophysics and British Heart Foundation Centre of Research Excellence, King’s College London, London SE1 1UL, United Kingdom

**Keywords:** Cardiac troponin, Cardiomyopathy, Heart failure, Small molecules

## Abstract

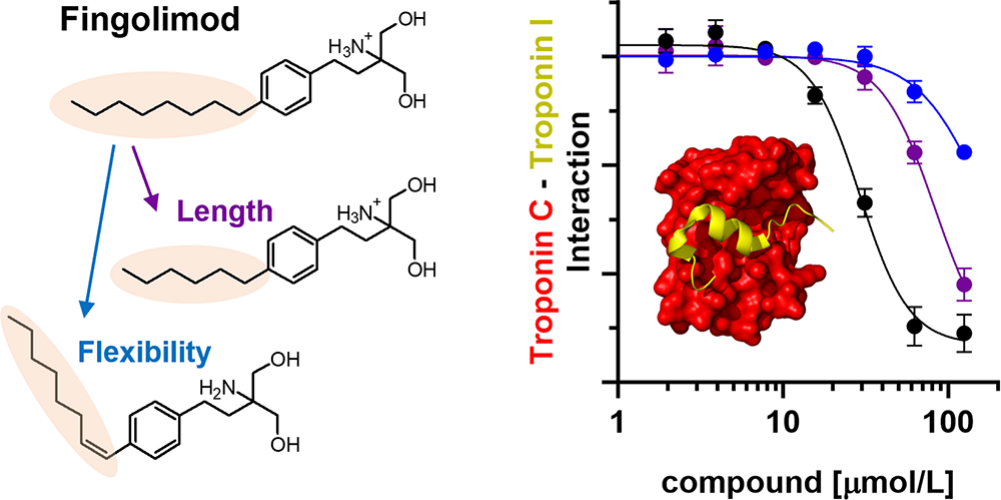

Calcium binding to cardiac troponin C (cTnC) in the thin filaments acts as a trigger for cardiac muscle contraction. The N-lobe of cTnC (NcTnC) undergoes a conformational change in the presence of calcium that allows for interaction with the switch region of cardiac troponin I (cTnI_SP_), releasing its inhibitory effect on the thin filament structure. The small molecule fingolimod inhibits cTnC–cTnI_SP_ interactions via electrostatic repulsion between its positively charged tail and positively charged residues in cTnI_SP_ and acts as a calcium desensitizer of the contractile myofilaments. Here we investigate the structure–activity relationship of the fingolimod hydrophobic headgroup and show that increasing the alkyl chain length increases both its affinity for NcTnC and its inhibitory effect on the NcTnC–cTnI_SP_ interaction and that decreasing flexibility completely abolishes these effects. Strikingly, the longer derivatives have no effect on the calcium affinity of cTnC, suggesting that they act as better inhibitors.

Contraction and relaxation of cardiac muscle is controlled by Ca^2+^ activation of the actin-containing thin filaments and mediated by the troponin–tropomyosin regulatory system^1^ (Figure 1A). The calcium-dependent interaction between the N-terminal lobe (N-lobe) of the troponin C subunit (NcTnC) and the switch region of the troponin I subunit (cTnI_SP_) leads to the azimuthal movement of tropomyosin on the surface of the thin filaments, exposing myosin-binding sites on actin^2^ (Figure 1A). Subsequently, myosin heads from the neighboring thick filaments can strongly attach to actin and, fueled by the hydrolysis of ATP, undergo the “working stroke”, leading to pN-scale force development or nanometer-scale displacement of the thin filaments toward the center of the sarcomere. Conversely, Ca^2+^ dissociation from NcTnC reduces its intermolecular interaction with cTnI_SP_, leading to tropomyosin being positioned to block myosin-binding sites on actin, the dissociation of myosin heads from actin, and the onset of mechanical relaxation of the heart.

**Figure 1:**
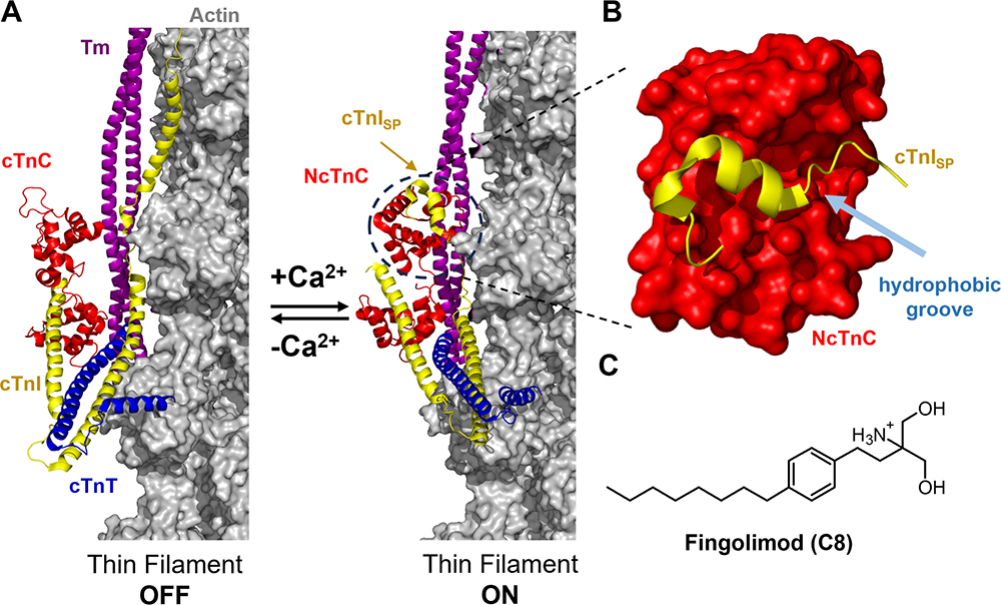
**Thin filament activation in cardiac muscle.** (A) Cryo-electron microscopy-based models of the cardiac thin filament in the absence (left, PDB entry 7KO4) or presence of Ca^2+^ (right, PDB entry 7KO5). Troponin C (cTnC), troponin I (cTnI), troponin T (cTnT), tropomyosin (Tm), and actin are shown in red, yellow, blue, purple, and gray, respectively. (B) Surface representation of the N-lobe of cTnC (NcTnC) bound to the switch region of troponin I (cTnI_SP_) shown in cartoon representation. (C) Chemical structure of fingolimod.

The Ca^2+^-dependent interaction between NcTnC and cTnI_SP_ is an attractive target for the development of new therapeutic interventions for both cardiomyopathies and heart failure^3,4^ (Figure 1B). Directly targeting the contractile myofilaments has some potential key advantages over traditional therapies based on neurohumoral modulation or changes in the intracellular Ca^2+^ transient, which are associated with unwanted side effects such as arrhythmias, tachycardias or bradycardias, myocardial energy wastage, or systemic side effects.^5,6^ Small-molecule effectors that could either increase or decrease the interaction might shift the balance between myocardial activation and relaxation and therefore could be useful interventions to treat both systolic and diastolic heart failure, respectively.^3^ However, the interaction interface between troponin C and troponin I is not muscle isoform-specific, complicating the development of a cardiac-muscle-specific therapeutic intervention.^7^

Several small-molecule effectors that target cardiac troponin have been extensively studied, such as levosimendan, EGCg, bepridil, W7, trifluoperazine, and others.^4,8^ However, currently only the troponin agonist levosimendan is prescribed as part of a heart failure therapy, but even in this case the molecular targets and precise mechanism of action are not well-characterized.^9,10^ Further progress in the development of novel heart failure therapies based on small-molecule modulators of troponin is likely hampered by the fact that most compounds were identified based on either homology to other EF-hand group proteins,^11^ as additives during structural studies or phenotypical assays that usually do not allow the direct identification of the drug target.^12^ It follows that the majority of currently studied compounds are either promiscuous *in vivo* or show low affinity.

To overcome this, we have developed *in vitro* screening assays that allow for the identification of both activators and inhibitors of the NcTnC–cTnI_SP_ interface^13,14^ and have identified fingolimod as an antagonist that decreases myocardial calcium sensitivity by reducing the affinity of NcTnC for cTnI_SP_^14^ (Figure 1C). However, fingolimod also increases the Ca^2+^ affinity of isolated NcTnC, which is a common feature of troponin-directed small-molecule effectors, likely by stabilizing its open conformation.^15,16^ We proposed that the balance between NcTnC’s affinity for cTnI_SP_ and Ca^2+^ determines whether a compound acts as a cardiac muscle activator or inhibitor.

Most troponin-directed small-molecule effectors have a similar structure with a hydrophobic headgroup that anchors the molecule to the hydrophobic groove of NcTnC (Figure 1B) and a polar or charged tail that likely interacts with residues on cTnI. We previously investigated fingolimod’s structure–activity relationship by replacing its positively charged ammonium group with a negatively charged carboxyl group, which abolished its inhibitory effect.^14^ This is in good agreement with analogue experiments performed with the inhibitor W-7,^17^ suggesting that a positively charged tail is necessary to reduce the interaction between NcTnC and cTnI_SP_, likely by mediating electrostatic repulsion between the amino group and positively charged residues on cTnI_SP_.

In the current study, we assessed the effect of fingolimod’s hydrophobic headgroup on its structure–activity relationship. We synthesized four new fingolimod derivatives which deviate from the parent compound by one or two carbons either way to give the hexyl, heptyl, nonyl, and decyl derivatives (Schemes S1 and S2 and Figure 2A). We subsequently refer to this group of compounds as C_6_–C_10_-fingolimod.

**Figure 2:**
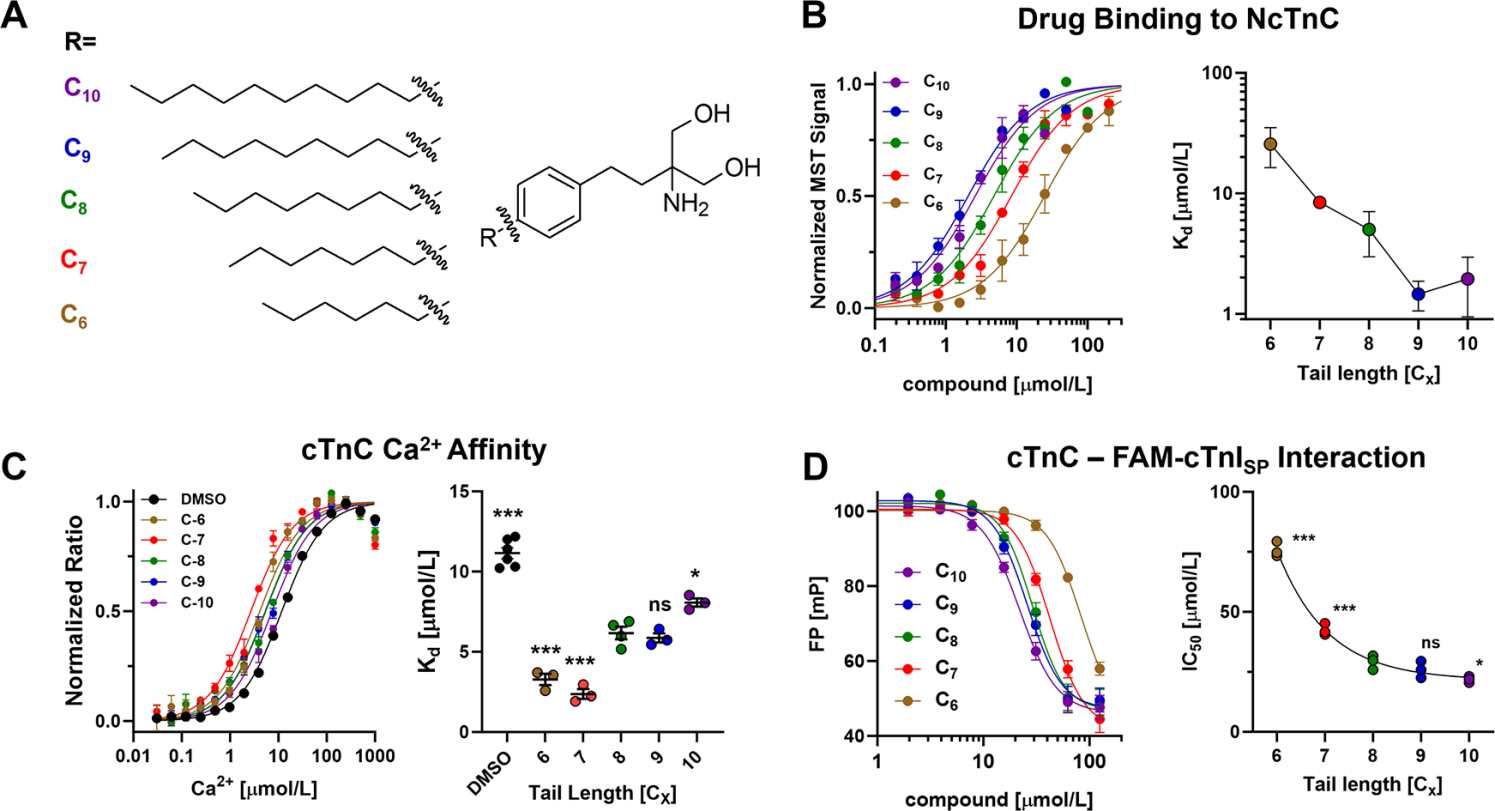
**Biophysical characterization of fingolimod derivatives binding to NcTnC and cTnC.** (A) Chemical structure of fingolimod derivatives with different alkyl chain lengths. (B) Normalized microscale thermophoresis binding curves for fingolimod derivatives titrated against Alexa647-labeled N-terminal domain of cTnC (NcTnC). (C) Ca^2+^ binding to full-length cTnC was monitored by changes in BADAN fluorescence in the absence or presence of 200 μmol/L compounds. (D) Competitive titration of compounds into a mixture of full-length cTnC and FAM-labeled cTnI switch peptide (FAM-cTnI_SP_) monitored by fluorescence polarization (FP). Means ± SEM, *n* = 2–5 independent repeats. Statistical significance of differences between the parent compound (C_8_) and other derivatives was assessed with a one-way ANOVA followed by Tukey’s post-hoc test: *, *p* < 0.05; **, *p* < 0.01; ***, *p* < 0.001; ns, not significant.

The affinity of the fingolimod series of compounds to the isolated N-lobe of cTnC (NcTnC) in the presence of Ca^2+^ was determined by microscale thermophoresis (Figure 2B and Table 1). The parent compound C_8_-fingolimod binds NcTnC with a steady-state dissociation constant *K*_d_ of about 5 μmol/L. Decreasing the alkyl chain length by one or two methylene groups decreased the affinity with *K*_d_ of about 8 and 25 μmol/L, respectively. Conversely, increasing the length of the alkyl chain increased the affinity by about a factor of 2–3 with a *K*_d_ of about 2 μmol/L for both the C_9_ and C_10_ derivatives. This suggests that the interaction between fingolimod and NcTnC is mainly mediated via hydrophobic interactions of its alkyl moiety with the hydrophobic groove of NcTnC.

**Table 1:** Summary of Biophysical Parameters for the Effect of Fingolimod Derivatives on Cardiac Troponin C Ca^2+^ Affinity and Interaction with cTnI Switch Peptide^a^

	*K*_d_ [μmol/L]		
	drug binding to NcTnC	NcTnC Ca^2+^ affinity	cTnC–cTnI_SP_ IC_50_ [μmol/L]
control	–	11.2 ± 0.3	–
C_6_-fingolimod	25.7 ± 6.6	3.3 ± 0.3	75.8 ± 1.8
C_7_-fingolimod	8.4 ± 0.1	2.4 ± 0.3	42.5 ± 1.4
C_8_-fingolimod	5.0 ± 1.4	6.2 ± 0.4	29.1 ± 1.7
C_9_-fingolimod	1.5 ± 0.3	5.9 ± 0.3	26.0 ± 2.0
C_10_-fingolimod	1.9 ± 0.7	8.1 ± 0.3	21.9 ± 0.8
*Z*-fingolimod	77.3 ± 13.1	10.4 ± 0.7	cnd

a Means ± SEM, *n* = 2–5. cnd: cannot be reliably determined.

Next, we used saturating concentrations of fingolimod derivatives to test for their effect on cTnC calcium affinity (Figure 2C and Table 1). We monitored Ca^2+^ binding to cTnC by changes in the fluorescence of a BADAN probe attached to cysteine-84.^14^ As reported previously, the parent compound C_8_-fingolimod significantly increased the cTnC Ca^2+^ affinity, as indicated by a decrease in the *K*_d_ from about 12 μmol/L under control conditions to 6 μmol/L in the presence of the compound. Reducing the alkyl chain length further decreased the *K*_d_ to about 2–3 μmol/L for both the C_6_ and the C_7_ derivatives. Strikingly, however, increasing the alkyl chain length had the opposite effect and decreased cTnC calcium affinity to values close to the control.

We titrated increasing concentrations of these derivatives into a mixture of 6-carboxyfluorescein (FAM)-labeled cTnI switch peptide (FAM-cTnI_SP_) and full-length cTnC in the presence of saturating [Ca^2+^] and monitored the displacement of the peptide from the complex by fluorescence polarization from the FAM probe (Figure 2D and Table 1). In very good agreement with the affinity measurements described above, shortening fingolimod’s alkyl chain significantly increased the IC_50_ for displacing FAM-cTnI_SP_ from cTnC, whereas the longer variants showed lower IC_50_ values, suggesting that the longer derivatives were more efficient in inhibiting NcTnC–cTnI_SP_ interactions.

Taken together, these results suggest that derivatives with longer alkyl chains are better inhibitors that bind more tightly to isolated cTnC and therefore more efficiently inhibit its interaction with the switch region of cTnI. More strikingly, however, although the longer derivatives bind more tightly, the effect on cTnC calcium affinity is markedly reduced, suggesting that this series of compounds might act as a better calcium desensitizer of the contractile myofilaments.

We next tested whether conformational flexibility in the fingolimod alkyl chain is required for its inhibitory effect on the cTnC–cTnI_SP_ interaction. We synthesized a derivative with a double bond between C15 and C16 (Scheme S3 and Figure 3A), yielding the *Z* isomer in a 9:1 ratio over the *E* isomer (subsequently referred to as “*Z*-fingolimod”). Strikingly, introduction of the double bond strongly reduced NcTnC affinity (*K*_d_ of about 80 μmol/L vs 5 μmol/L for C_8_; Figure 3B and Table 1) and almost completely abolished the inhibitory effect on the cTnC–cTnI_SP_ interaction (Figure 3C), as indicated by an increase in the IC_50_ in the fluorescence polarization displacement assay from about 30 μmol/L to more than 150 μmol/L. Similarly, 200 μmol/L *Z*-fingolimod had no effect on the calcium affinity of cTnC as reported from BADAN fluorescence (Figure 3D and Table 1).

**Figure 3:**
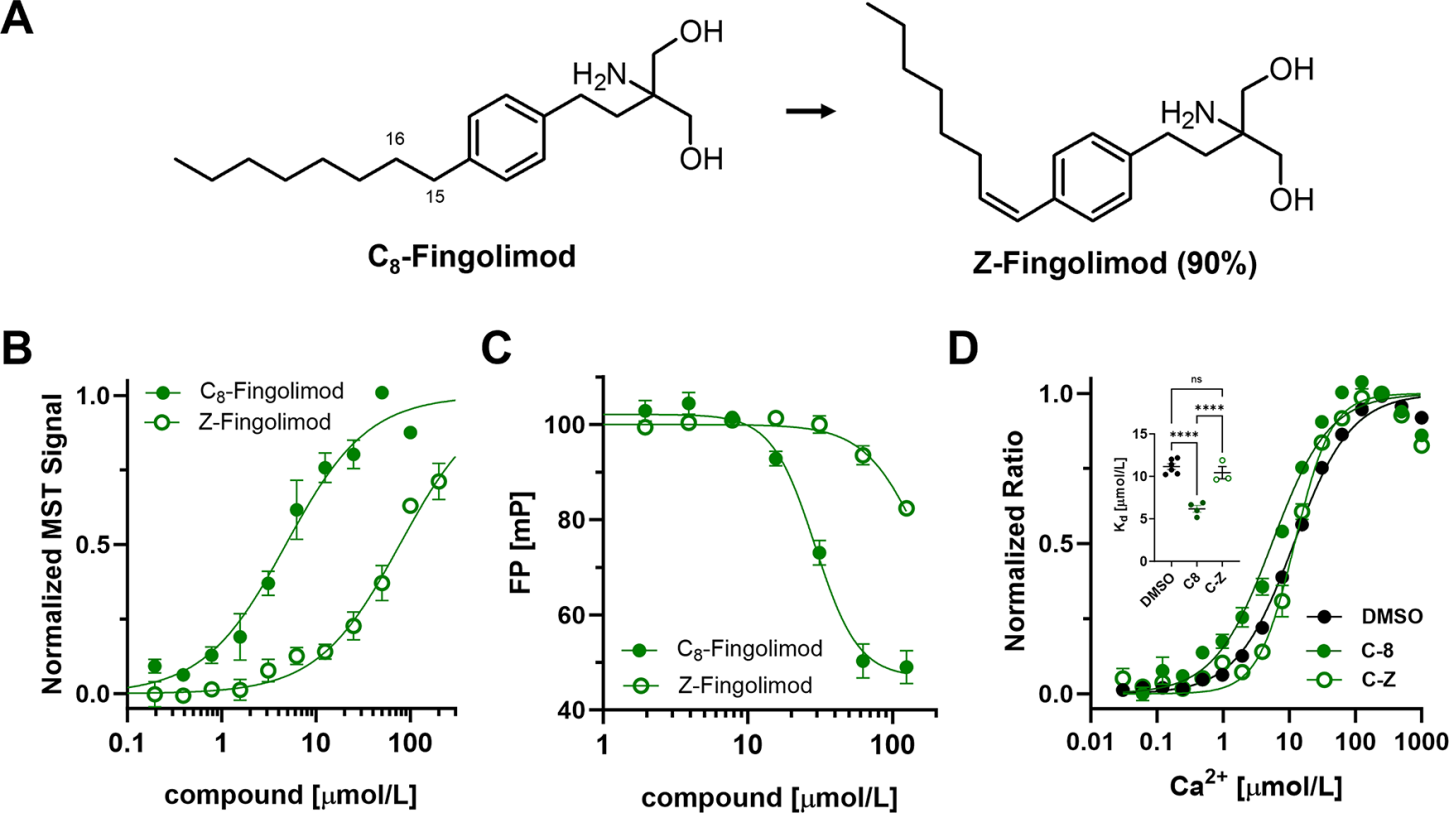
**Effect of alkyl chain flexibility on cTnC inhibition by fingolimod.** (A) Chemical structures of C_8_-fingolimod and *Z*-fingolimod. (B) Normalized microscale thermophoresis binding curves for C_8_-fingolimod and *Z*-fingolimod titrated against Alexa647-labeled N-terminal lobe of cTnC (NcTnc). (C) Competitive titration of compounds into a mixture of full-length cTnC and FAM-labeled cTnI switch peptide monitored by fluorescence polarization (FP). (D) Ca^2+^ binding to full-length cTnC monitored by changes in BADAN fluorescence in the absence or presence of 100 μmol/L compounds. Means ± SEM, *n* = 3–5 independent repeats. Statistical significance of differences between two groups was assessed with an unpaired two-tailed Student’s *t* test and between more than two groups with one-way ANOVA followed by Tukey’s multiple comparison test: ***, *p* < 0.001; ****, *p* < 0.0001; ns, not significant.

The functional effects of fingolimod derivatives were tested in isolated bovine cardiac myofibrils (CMFs) with intact thin and thick filaments organized into the native myofilament lattice. CMFs were pre-incubated with 200 μmol/L compounds in buffer containing suboptimal [Ca^2+^], corresponding to about 70% maximal activation, and the ATPase activity was measured in the presence of 2.5 mmol/L ATP (Figure 4). In excellent agreement with the biophysical characterization described above, increasing the aliphatic tail length of fingolimod increased its inhibitory effect on the CMF ATPase activity from about 20% for the C_6_ derivative to about 80% for C_10_-fingolimod. Moreover, *Z*-fingolimod showed a significantly lower inhibitory effect on the ATPase activity than the parent compound C_8_-fingolimod, which is in excellent agreement with its lower affinity for NcTnC and its effect on the cTnC–cTnI_SP_ interaction (Figure 3).

**Figure 4:**
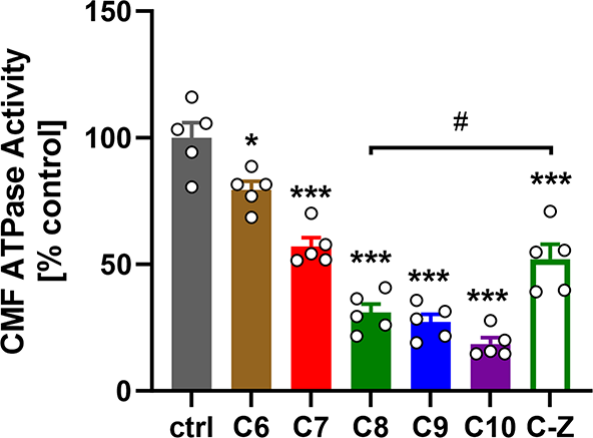
**Effect of fingolimod derivatives on the ATPase activity of bovine cardiac myofibrils (CMFs).** ATPase activity of CMFs was measured at suboptimal [Ca^2+^] concentration (pCa = 5.2, corresponding to about 70% maximal activation) in the presence of 200 μmol/L compounds. Statistical significance of differences between values was assessed with a one-way ANOVA followed by Tukey’s multiple comparison test: *, *p* < 0.05; ***, *p* < 0.001 for compounds vs ctrl and ^#^, *p* < 0.05 for C_8_ vs *Z*. Means ± SEM, *n* = 5.

In summary, in this note we have shown that increasing the length of the hydrophobic alkyl chain of fingolimod significantly increases its inhibitory effect on thin filament activation by increasing its affinity toward NcTnC and reducing NcTnC’s affinity toward the switch region of cTnI. More importantly, however, derivatives with longer alkyl chains reduce the effect on NcTnC Ca^2+^ affinity, further shifting the equilibrium toward inhibition and acting as better inhibitors of the contractile myofilaments. This suggests that directly modulating the balance between increased calcium affinity and decreased cTnI_SP_ interaction of NcTnC can be achieved by specifically modulating the interaction of the hydrophobic core of NcTnC-directed small-molecule effectors. Moreover, we have shown that reducing the flexibility of fingolimod reduces its inhibitory effect, suggesting that conformational flexibility in the fingolimod aliphatic tail is required to provide better binding of the compound to troponin C to modulate function.^18^

However, the development of fingolimod as a negative cardiac inotrope is complicated by the fact that it was developed as a sphingosine-1-phosphate (S1P) receptor modulator, which affects cardiac cell survival, hypertrophy, and contractile function via attenuation of S1P receptor subtype-1 signaling.^19^ Moreover, S1P receptors have important functions in endothelial and smooth muscle cells, controlling peripheral vascular tone, and a wide range of endothelial responses.

## Supporting Information Available

Experimental procedures for production of proteins and peptides, microscale thermophoresis, fluorescence intensity measurements, fluorescence polarization measurements, myofibrilar atpase activity measurements, and synthesis of fingolimod derivatives (Schemes S1–S3) and NMR, IR, and HRMS spectra of compounds (PDF)

## Abbreviations

cTnI_SP_: switch region of cardiac troponin I
FAM: 6-carboxyfluorescein
NcTnC: N-terminal lobe of cardiac troponin C
S1P: sphingosine-1-phosphate
TnC: troponin C
