# Supplementary material for: Synthesis and Biophysical Characterization of Fingolimod Derivatives as Cardiac Troponin Antagonists
Source: ACS Med Chem Lett. 2024 Feb 7;15(3):413–7. doi: 10.1021/acsmedchemlett.3c00511 (PMC10945792; doi:10.1021/acsmedchemlett.3c00511)
Supplement: Supplementary file 1 — ml3c00511_si_001.pdf [file ml3c00511_si_001.pdf]

Supporting Information

Synthesis and biophysical characterisation of fingolimod derivatives as cardiac troponin antagonists

Laszlo Kondacs[1], Priyanka Parijat[2], Alexander J. A. Cobb[1,*] and Thomas Kampourakis[2,*]

[1]Department of Chemistry, Britannia House, King's College London, SE1 1DB, United Kingdom

[2]Randall Centre for Cell and Molecular Biophysics, and British Heart Foundation Centre of Research Excellence, King's College London, London, SE1 1UL, United Kingdom

*corresponding authors: thomas.kampourakis@kcl.ac.uk
andre.cobb@kcl.ac.uk

Table of Contents

Experimental Procedures………………………………………………………..……… 3

Production of Proteins and Peptides……………………………………………………….3

Microscale Thermophoresis………………………………………………………………... 3

Fluorescence Titrations…………………………………………………………………….....3

Fluorescence Polarization Assays……………………………………………………………… 3

Myofibrilar ATPase Activity Measurements……………………………………………………..3

Synthesis of Fingolimod Derivatives…………………………………………………………... 5

NMR, IR and HRMS Spectra of Compounds…………………………………..……….15

Supporting Information References……………………………….………………….62

Experimental Procedures

Production of Proteins and Peptides

Full length human cardiac troponin C (cTnC) and its N-terminal domain (NcTnC) were expressed from a modified pET6a vector in BL21 (DE3)-RIPL cells (Agilent Technologies Inc.) fused to a hexa-histidine tag and TEV protease site. Proteins were purified on HisTrapFF columns (GE Heatlhcare), and histidine-tag was removed by treating proteins overnight with 1:100 stoichiometry of hexahistidine-tagged TEV protease. TEV protease and histidine-tag were removed by applying the mixture onto a 1 ml HisTrapFF column, and the flow-through containing the purified protein was collected. Protein purity was estimated to be > 95% by SDS-PAGE and electron spray ionization mass spectrometry (ESI–MS). The N-terminally 6-carboxy-fluoresceine (FAM) labelled A162H-mutated switch region peptide of human cardiac troponin I (sequence: TLRRVRISADAMMQALLGARHKESLDLR; C-terminus is free acid) was purchased from Fisher Scientific (> 99% purity, TFA removed).

Microscale Thermophoresis

Recombinant NcTnC was labelled with Alexa647-NHS (ThermoScientific) at a stoichiometry 1:1 according to the manufacturer's instructions. The reactions were stopped with 50 times molar excess Tris–HCl and cleaned of free dye using a Nap5 column. Dye incorporation efficiency (>80%) was confirmed by HPLC and using the Alexa647 extinction coefficient. MST buffer contained 20 mmol/l Mops, pH 7, 1 mmol/l $CaCl_2$, 1 mmol/l $MgCl_2$, 50 mmol/l KCl, 1 mmol/l DTT, 0.05% (v/v) Tween 20. Twelve (1:1) dilutions were prepared, producing ligand C0C2 concentrations ranging from 0.096 μmol/L to 200 μmol/L. The concentration of Alexa647-labeled NcTnC was 50 nmol/l. After 20 min incubation with drugs, followed by centrifugation at maximum speed in a table-top centrifuge for 1 min, the samples were loaded into Monolith NT.115 [Premium] Capillaries (NanoTemper Technologies). Experiments were performed at 20% LED power, 80% MST power, and 30 °C.

Fluorescence Titrations

Fluorescence titration experiments were performed in 20 mmol/L MOPS, pH 7, 100 mmol/L KCL, 1 mmol/L $MgCl_2$, and 1 mmol/L DTT, with a fixed BADAN-cTnC concentration of 1 μmol/L. BADAN-cTnC was excited at 380 nm, and fluorescence intensities were measured between 400 and 600 nm using the ClarioStar Plate reader. Raw fluorescence spectra were smoothed using a sliding mean algorithm and fluorescence intensity ratios extracted in MARS analysis software (BMG LabTech).

Fluorescence Polarization Assay

Fluorescence polarization dose–response experiments were performed in Greiner 384-well black plates in assay buffer (20 mmol/L Tris–HCl pH 7, 100 mmol/L NaCl, 1 mmol/L $CaCl_2$, 1 mmol/L DTT) at 25 °C containing 5 μmol/L FAM-labelled switch peptide and 5 μmol/L cTnC. Fluorescence polarization from each well was measured using a ClarioStar Plate reader with appropriate excitation and emission filter settings. Dose–response curves were constructed by serial dilution of compounds in DMSO and adding a fixed volume to a mixture of peptide and cTnC.

Myofibrillar ATPase Activity Measurements

Cardiomyofibrils (CMFs) were prepared by homogenizing freshly frozen bovine ventricular tissue samples in myofibril buffer (composition in mmol L^{-1}: 20 imidazole pH 7.4, 75 KCl, 2 MgCl2, 2 EDTA, 1 DTT, 1% (v/v) Triton X-100, protease inhibitor cocktail (Roche), PhosStop cocktail (Roche)) followed by centrifugation at 5000 g for 5 min at 4°C. CMFs were washed

and homogenized three more times in the same buffer without Triton X-100. CMFs were washed three times in ATPase assay buffer (composition in mmol L−1: 20 MOPS pH 7.0, 35 NaCl, 5 MgCl2, 1 EGTA, 1 DTT) and $CaCl_2$ was added to achieve the desired free [Ca^{2+}] (pCa 5.2). CMF concentrations were adjusted to 1 mg mL^{-1} , compounds or vehicle control (DMSO) were added and the mixture incubated at 30ºC at constant shaking for 10 mins. Reactions were started by the addition of 2.5 mmol L^{-1} Na_2ATP and samples quenched with 0.5 volumes ice cold 25% (w/v) TCA solution after 5 mins. Samples were kept on ice at all times, diluted with double-deionized water and inorganic phosphate content measured using the malachite green assay according to manufacturer's instructions (Sigma, MAK030).

Synthesis of Fingolimod Derivatives

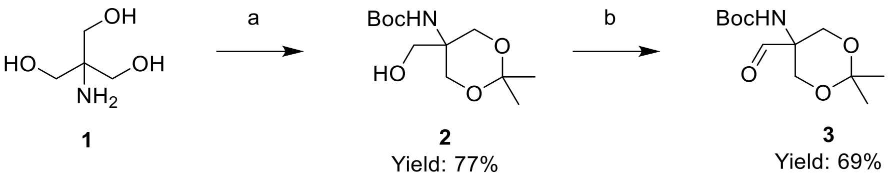

Scheme S1. *Synthesis of intermediate aldehyde* ***3****. Reagents and conditions: (a) (i), $(Boc)_2O$, DMF, r.t., (ii), 2,2-dimethoxypropane, p-toluenesulfonic acid, DMF, r.t.; (b) oxalyl chloride, DMSO, triethylamine, DCM, -78 °C.*[1]

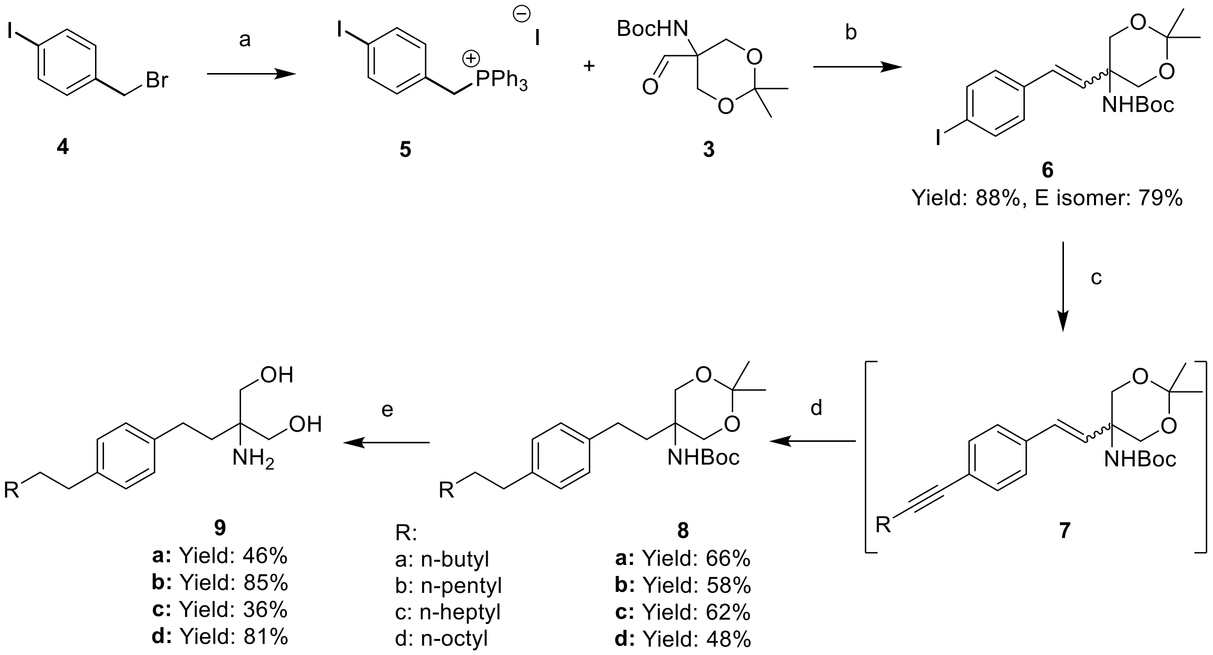

Scheme S2. *Synthesis of Fingolimod derivatives with different alkyl chains* ***9a-d****. Reagents and conditions: (a) PPh_3, toluene, reflux;*[2] *(b) K_2CO_3, THF, DMF, reflux; (c) PPh_3, Pd/C, CuI, triethylamine, acetonitrile, MW, 80 °C; (d) H_2, Pd/C, EtOAc, r.t.; (e) TFA, DCM, r.t..*[1]

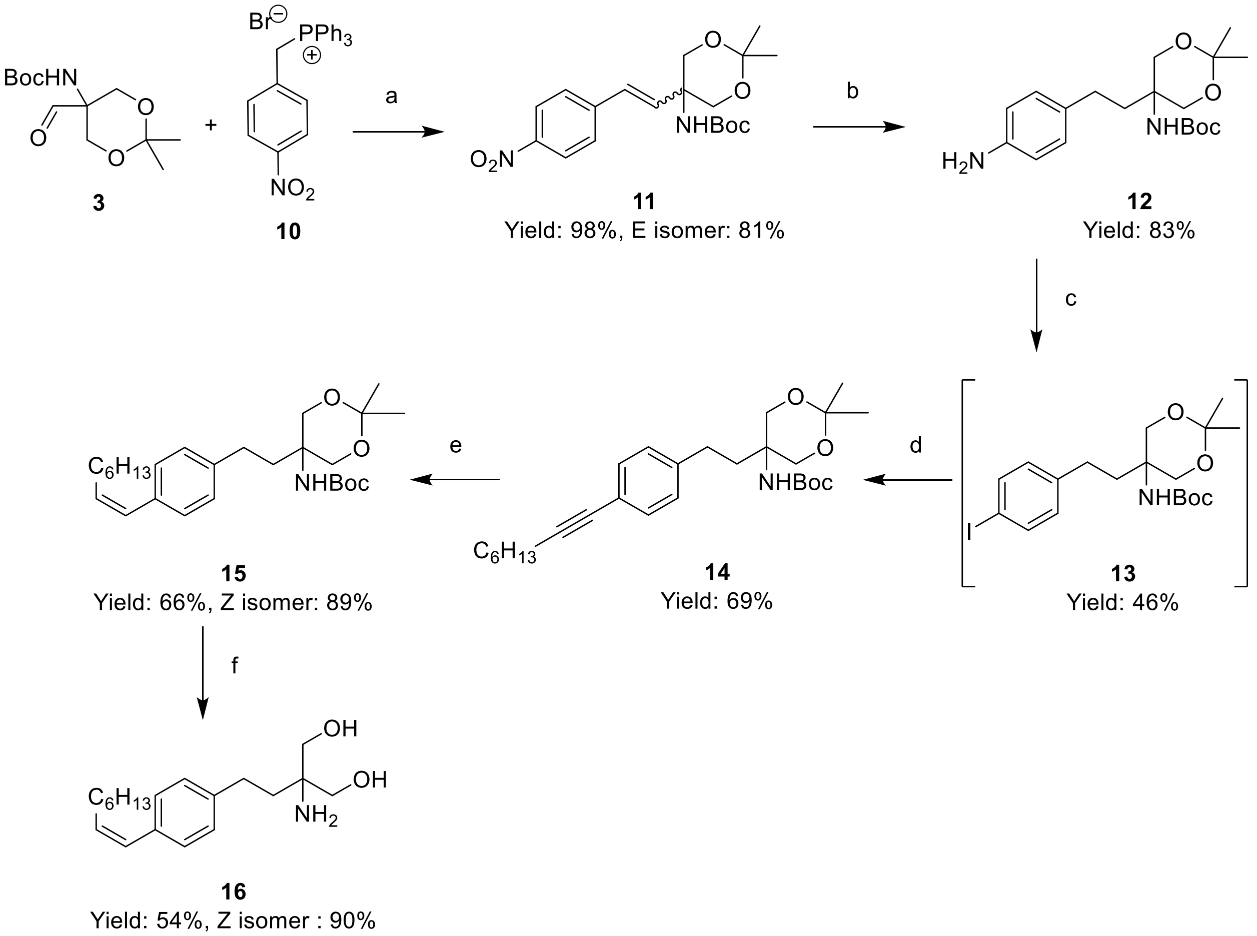

Scheme S3. *Synthesis of Fingolimod derivative with alkynyl chain **13**. Reagents and conditions: (a) K_2CO_3, THF, DMF, reflux; (b) H_2, Pd/C, methanol, r.t.; (c) tBuONO, I_2, toluene, r.t.; d: PPh_3, Pd/C, CuI, triethylamine, acetonitrile, MW, 80 °C; e: H_2, Lindlar catalyst, quinoline, ethyl acetate, r.t.; f: TFA, DCM, r.t..*

General information:

All commercially available reagents and solvents were obtained from Sigma-Aldrich, Apollo Scientific, Alfa Aesar, Fluorochem, VWR or Fischer Scientific and were used without further purification. Melting points were recorded on Stuart SMP30 melting point apparatus and are uncorrected. Infrared spectra were recorded in the range 4000 – 600 cm^{-1} using a Shimadzu IRAffinity-1S FTIR Spectrophotometer as a thin film. Bruker Ascend 400 at 400 MHz for 1H spectra or at 100 MHz for 13C spectra. Chemical shifts (δ) are quoted in parts per million (ppm) using the abbreviations: s: singlet; d: doublet; dd: double of dublets; t: triplet; quart: quartet; quint: quintet. Signals that could not be interpreted were designated multiplets (m) or broad (br). Coupling constants J are quoted in Hz. High resolution accurate mass measurements were collected by King’s College London mass spec service with an Acquity UPLC coupled to a Xevo G2-XS QTOF and UCL Mass Spectrometry Facility on an Orbitrap Q Exactive mass spectrometer. Teledyne ISCO CombiFlash Rf+ was used for flash chromatography, the samples were pre-absorbed onto silica 60 (40-63 micron) or injected as a solution of the weak solvent. Air and moisture sensitive reactions were carried out in oven dried glassware under a nitrogen atmosphere. Microwave reactions were carried out in a Biotage Initiator+ microwave synthesizer. Hydrogenation reactions were performed in Parr 3929 Hydrogenation
Apparatus under pressure, and with balloons under atmospheric pressure. Thin layer chromatography was performed on VWR silica gel 60 F254. Thin layer chromatography results were analysed by UV lamp (254 nm), ninhydrin stain (amine, amide content) and/or ceric ammonium molybdate stain (oxidising agent). All compounds are >95% pure by HPLC analysis.

General method A for Wittig reaction:

To the suspension of compound **3** (1.7 g, 6.6 mmol) in THF (30 mL) and DMF (10 mL) mixture the appropriate triphenylphosphonium bromide (7.9 mmol) and K_2CO_3 was added under inert atmosphere and the mixture was heated to reflux and stirred overnight. After the reaction was complete, THF was removed under reduced pressure, the residue was quenched with water, and extracted with ethyl acetate (2 x 60 mL). The combined organic phases was washed with brine (60 mL), dried over $MgSO_4$ and the solvent was removed under reduced pressure. The crude was purified by flash chromatography (eluent: hexane – ethyl acetate) to give the product as an E/Z isomer mixture.

General method B for Sonogashira coupling:

To the solution of the appropriate aryl iodide (0.87 mmol) in dry degassed acetonitrile (6 mL) triphenylphosphine (23.0 mg, 0.087 mmol), 10% palladium on carbon (92.0 mg, 0.087 mmol), copper(I) iodide (8.3 mg, 0.044 mmol) and triethylamine (0.36 mL, 2.61 mmol) were added, and the mixture was stirred for 25 minutes at room temperature. This was followed by the addition of the appropriate alkyne (1.31 mmol), then the reaction was stirred for 1 hour at room temperature and for 90 minutes at 80 °C in a microwave reactor. After the reaction was complete, it was diluted with ethyl acetate (6 mL) and filtered through celite. The crude was purified by flash chromatography (eluent: hexane – ethyl acetate) to give the product.

General method C for reducing simultaneously alkene and alkyne moieties:

To the solution of the appropriate compound **7** (0.32 g, 0.77 mmol) in ethyl acetate (20 mL) 10% palladium on carbon (0.032 g) was added portion wise and the mixture was hydrogenated under 30 psi for 2 hours. The incomplete reaction mixture was filtrated through celite, then the filtrate was purified by flash chromatography (eluent: hexane – ethyl acetate). This was further hydrogenated in ethyl acetate (20 mL) in the presence of 10% palladium on carbon (0.032 g) overnight under atmospheric pressure. After the reaction was complete, it was filtered through celite. The crude was purified by flash chromatography (eluent: hexane – ethyl acetate) to give the product **8**.

General method D for simultaneous Boc and ketal deprotection:

The solution of the appropriate compound (0.54 mmol) in the mixture of TFA (3 mL), dichloromethane (3 mL) and water (1.5 mL) was stirred overnight. After the reaction was complete, it was diluted with water (6 mL), neutralised with saturated $NaHCO_3$ solution (30 mL) and extracted with chloroform methanol mixture (4:1 ratio 4 x 20 mL). The combined organic phases was washed with water (20 mL), dried over $MgSO_4$ and evaporated under reduced pressure. The crude was purified by flash chromatography (eluent: dichloromethane – methanol) to give the pure product as white solid. This solid was participated between 1M NaOH aqueous solution (10 mL) and dichloromethane (10 mL). The organic phase was dried over $MgSO_4$ and evaporated under reduced pressure to give the pure free amine.

tert-Butyl-5-(hydroxymethyl)-2,2-dimethyl-1,3-dioxan-5-ylaminoformylate **2**[1]

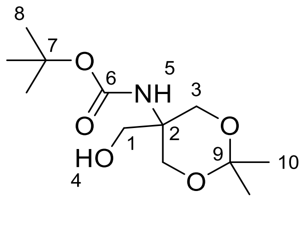

To the suspension of tris base **1** (7.00 g, 58.0 mmol) in DMF (100 mL) Boc anhydride (14.6 mL, 63.0 mmol) was added and the mixture was stirred for 2 hours at room temperature. To this solution 2,2-dimethoxypropane (8.51 mL, 69.0 mmol) and p-toluenesulfonic acid (0.55 g, 3.2 mmol) were added and the resulting mixture stirred overnight at room temperature. After

the reaction was complete it was diluted with diethyl ether (100 mL), washed with 5% aqueous Na_2CO_3 (50 mL), water (50 mL) and brine (50 mL), it was dried over $MgSO_4$ and the solvent was removed under reduced pressure to give the crude product as a white solid (11.67 g, 77%). Despite some Boc group containing impurity it was carried on to the next step without further purification. Mp: 81-84 °C; ^{1}H NMR (400 MHz, DMSO-d$_6$) δ_H 6.35 (1H, br s, 5-H), 4.73 (1H, t, *J*=5.6 Hz, 4-H), 3.94 (2H, d, *J*=10.8 Hz, 3a-H), 3.67 (2H, d, *J*=11.6 Hz, 3b-H), 3.59 (2H, d, *J*= 6.0 Hz, 1-H), 1.47 (9H, s, 8-H), 1.37 (3H, s, 10a-H), 1.27 (3H, s, 10b-H); ^{13}C NMR (101 MHz, DMSO-d$_6$) δ_c 146.7 (6-C), 97.9 (9-C), 86.1 (7-C), 62.1 (3-C), 60.9 (1-C), 52.7 (2-C), 27.3 (8-C), 26.2 (10a-C), 21.9 (10b-C); ν_{max}/cm^{-1} 3264 (O-H, N-H), 1680 (C=O), 1560 (N-H) 1161 (C-O); HRMS (Found (M+Na)$^+$ 284.1454. Calcd. for $C_{12}H_{23}O_5N$: (M+Na)$^+$ 284.1468.).

tert-Butyl-5-formyl-2,2-dimethyl-1,3-dioxan-5-ylaminoformylate **3**[1]

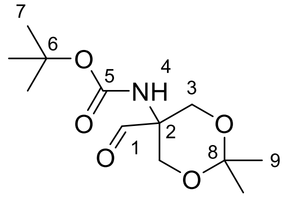

To the solution of oxalyl chloride (3.24 mL, 38.0 mmol) in DCM (50 mL) DMSO (4.04 mL, 57 mmol) was added dropwise at -78 °C and it was stirred for 15 minutes. It was followed by the dropwise addition of the solution of compound **2** (5.00 g, 19.0 mmol) in DCM (50 mL), after 15 minutes stirring triethylamine was added dropwise and the mixture was stirred at -78 °C for 15 minutes and at room temperature for 30 minutes. After the reaction was complete, it was quenched with water (70 mL) and the aqueous phase was extracted with DCM (2 x 50 mL). The combined organic phases was washed with 1% aqueous sulfuric acid (50 mL), brine (2 x 50 mL), dried over $MgSO_4$ and the solvent was removed under reduced pressure. The crude was purified by flash chromatography (eluent: hexane – ethyl acetate) to give the pure product as white solid (3.4 g, 69%). Mp: 112-113 °C; ^{1}H NMR (400 MHz, $CDCl_3$) δ_H 9.65 (1H, s, 1-H), 5.58 (1H, s, 4-H), 4.09 (2H, d, *J*=12.0 Hz, 9a-H), 3.97 (2H, d, *J*=11.6 Hz, 9b-H), 1.48 (15H, m, 7, 9-H); ^{13}C NMR (101 MHz, $CDCl_3$) δ_c 199.3 (1-C), 155.5 (5-C), 98.8 (8-C), 81.0 (6-C), 62.7 (3-C), 59.9 (2-C), 28.2 (7-C), 27.3 (9a-C), 19.6 (9b-C); ν_{max}/cm^{-1} 3323 (N-H). 1734 (C=O), 1695 (C=O), 1518 (N-H), 1165 (C-O); HRMS (Found (M+Na)$^+$ 282.1307. Calcd. for $C_{12}H_{21}O_5N$: (M+Na)$^+$ 282.1312.).

5-[(*E*)-2-(*p*-Iodophenyl)ethenyl]-5-(*tert*-butoxycarbonylamino)-2,2-dimethyl-1,3-dioxane **6**[1]

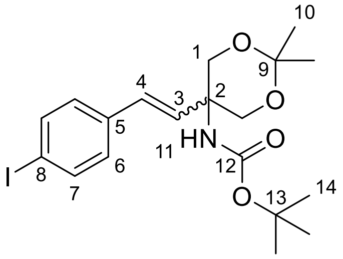

The Wittig reagent **5** was prepared by heating at reflux temperature the solution of triphenylphosphine (13.10 g, 50.0 mmol) and 4-iodobenzylbromide **4** (14.85 g, 50.0 mmol) in dry toluene (150 mL) under inert atmosphere overnight. The precipitate was filtered and washed with toluene to give the phosphonium salt **5** (26.43 g, 95%). Part of this reagent **5** (5.97 g, 10.7 mmol) was used in the synthesis of compound **6** following General method A. Compound **6** with Z isomer impurity (21%) was gained as white crystals (3.58 g, 88%). Mp: 105-107 °C; ^{1}H NMR (400 MHz, $CDCl_3$) δ_H 7.64 (2H, d, *J*=8.4 Hz, 7-H), 7.02 (2H, d, *J*=7.6 Hz,

6-H), 6.60 (1H, d, *J*=12.8 Hz, 3-H), 5.60 (1H, d, *J*=12.4 Hz, 4-H), 5.15 (1H, br-s, 11-H), 3.90 (2H, d, *J*=11.6 Hz, 1a-H), 3.76 (2H, d, *J*=12.0 Hz, 1b-H), 1.40 (3H, s, 10a-H), 1.39 (9H, s, 14-H), 1.37 (3H, s, 10b-H); ^{13}C NMR (101 MHz, $CDCl_3$) δ_c 154.4 (12-C), 137.0 (7-C), 131.3 (3-C), 130.6 (5,6-C), 130.4 (4-C), 98.2 (9-C), 92.6 (8-C), 79.4 (13-C), 65.9 (1-C), 52.5 (2-C), 28.3 (14-C), 28.1 (10a-C), 18.8 (10b-C); v_{max}/cm^{-1} 3412 (N-H), 1705 (C=O), 1504 (N-H), 1161 (C-O). 833 (C=O); HRMS (Found $(M+Na)^+$ 482.0800. Calcd. for $C_{19}H_{26}O_4NI$: $(M+Na)^+$ 482.0799.).

tert-butyl (5-(4-hexylphenethyl)-2,2-dimethyl-1,3-dioxan-5-yl)carbamate **8a**

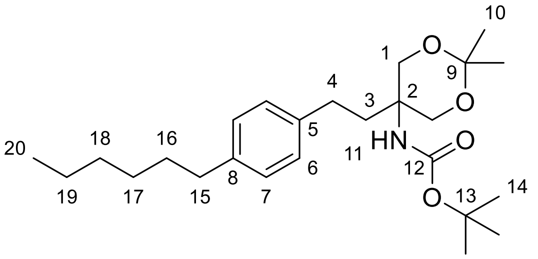

Compound **6** (0.40 g, 0.87 mmol) and hex-1-yne (0.15 mL, 1.31 mmol) were reacted following general method B to give compound **7a** (0.36 g), which was used in the next reaction step without further purification and characterisation following general method C. Compound **8a** was gained as white solid (0.24 g, 66%). Mp: 62-65 °C. ^{1}H NMR (400 MHz, $CDCl_3$) δ_H 7.11 (4H, s, 6, 7-H), 4.99 (1H, br s, 11-H), 3.92 (2H, d, *J*=11.6 Hz, 1a-H), 3.70 (2H, d, *J*=11.6 Hz, 1b-H), 2.57 (4H, m, 4, 15-H), 2.00 (2H, m, 3-H), 1.61 (2H, quint, *J*=6.8 Hz, 16-H), 1.50 (9H, s, 14-H), 1.46 (3H, s, 10a-H), 1.44 (3H, s, 10b-H), 1.32 (6H, m, 17 - 19-H), 0.90 (3H, m, 20-H); ^{13}C NMR (101 MHz, $CDCl_3$, mixture of rotamers – mr: major rotamer) δ_c 156.4^{mr}, 154.9 (12-C), 140.7, 140.5^{mr} (8-C), 139.1, 138.8^{mr} (5-C), 128.5, 128.4^{mr} (7-C), 128.2, 128.1^{mr} (6-C), 98.4 (9-C), 80.1^{mr}, 79.3 (13-C), 66.6, 66.4^{mr} (1-C), 59.4, 51.7^{mr} (2-C), 35.5 (4-C), 33.7 (3-C), 31.7 (18-C), 31.5 (16-C), 29.0 (17-C), 28.7 (15-C), 28.4^{mr}, 28.4 (14-C), 27.4 (10a-C), 22.6 (19-C), 19.8 (10b-C), 14.8 (20-C); v_{max}/cm^{-1} 3350 (N-H), 1697 (C=O), 1521 (N-H), 1170 (C-O); HRMS (Found $(M+Na)^+$ 442.2915. Calcd. for $C_{25}H_{41}O_4N$: $(M+Na)^+$ 442.2928.).

tert-butyl (5-(4-heptylphenethyl)-2,2-dimethyl-1,3-dioxan-5-yl)carbamate **8b**

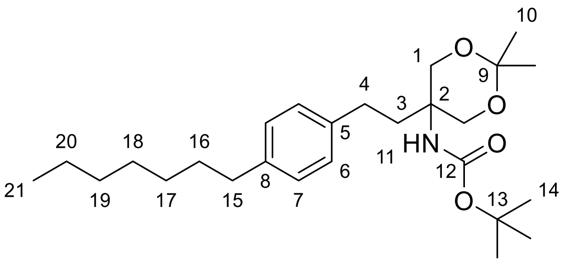

Compound **6** (0.40 g, 0.87 mmol) and hept-1-yne (0.17 mL, 1.31 mmol) were reacted following general method B to give compound **7b** (0.33 g), which was used in the next reaction step without further purification and characterisation following general method C. Compound **8b** was gained as white solid (0.22 g, 58%). Mp: 58-59 °C. ^{1}H NMR (400 MHz, $CDCl_3$) δ_H 7.11 (4H, m, 6, 7-H), 5.00 (1H, br s, 11-H), 3.92 (2H, d, *J*=11.6 Hz, 1a-H), 3.70 (2H, d, *J*=11.6 Hz, 1b-H), 2.56 (4H, m, 4, 15-H), 1.99 (2H, m, 3-H), 1.60 (2H, m, 16-H), 1.50 (9H, s, 14-H), 1.46 (3H, s, 10a-H), 1.44 (3H, s, 10b-H), 1.33 (8H, m, 17 – 20-H), 0.90 (3H, t, *J*=6.8 Hz, 21-H); ^{13}C NMR (101 MHz, $CDCl_3$, mixture of rotamers – mr: major rotamer) δ_c 156.5 (12-C), 140.7, 140.5^{mr} (8-C), 139.1^{mr}, 138.8 (5-C), 128.5, 128.5^{mr} (7-C), 128.2^{mr}, 128.1 (6-C), 98.4 (9-C), 80.2 (13-C), 66.6^{mr}, 66.4 (1-C), 59.4, 51.7^{mr} (2-C), 35.6 (15-C), 35.4^{mr}, 33.7 (3-C), 31.8 (20-C), 31.6 (16-C), 29.3 (18-C), 29.2 (17-C), 29.1, 28.6^{mr} (4-C), 28.5mr, 28.3 (14-C), 27.4 (10a-C), 22.7

(19-C), 19.7 (10b-C), 14.1 (21-C); v_{max}/cm^{-1} 3346 (N-H), 1670 (C=O), 1514 (N-H), 1169 (C-O); HRMS (Found $(M+H)^+$ 434.3261. Calcd. for $C_{26}H_{43}O_4N$: $(M+H)^+$ 434.3265.).

tert-butyl (2,2-dimethyl-5-(4-nonylphenethyl)-1,3-dioxan-5-yl)carbamate **8c**

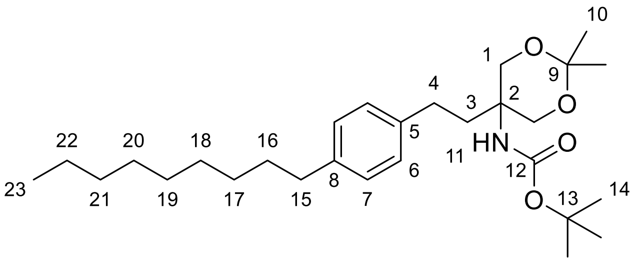

Compound **6** (0.40 g, 0.87 mmol) and non-1-yne (0.21 mL, 1.31 mmol) were reacted following general method B to give compound **7c** (0.41 g), which was used in the next reaction step without further purification and characterisation following general method C. Compound **8c** was gained as white solid (0.25 g, 62%). Mp: 46-48 °C. ^{1}H NMR (400 MHz, $CDCl_3$) δ_H 7.11 (4H, m, 6, 7-H), 5.00 (1H, br s, 11-H), 3.92 (2H, d, *J*=11.6 Hz, 1a-H), 3.70 (2H, d, *J*=11.6 Hz, 1b-H), 2.56 (4H, m, 4, 15-H), 1.99 (2H, m, 3-H), 1.59 (2H, m, 16-H), 1.50 (9H, s, 14-H), 1.46 (3H, s, 10a-H), 1.44 (3H, s, 10b-H), 1.28 (12H, m, 17 – 22-H), 0.90 (3H, t, *J*=6.4 Hz, 23-H); ^{13}C NMR (101 MHz, $CDCl_3$, mixture of rotamers – mr: major rotamer) δ_c 156.5 (12-C), 140.7, 140.5^{mr} (8-C), 139.1^{mr}, 138.8 (5-C), 128.6. 128.5^{mr} (7-C), 98.4 (9-C), 80.1 (13-C), 66.6, 66.4^{mr} (1-C), 59.4, 51.7^{mr} (2-C), 35.6 (15-C), 35.4^{mr}, 33.7 (3-C), 31.9 (22-C), 31.6 (16-C), 29.6 (17-C), 29.5 (18-C), 29.4 (19-C), 29.3 (20-C), 29.1, 28.7^{mr} (4-C), 28.5^{mr}, 28.4 (14-C), 27.5 (10a-C), 22.7 (21-C), 19.7 (10b-C), 14.1 (23-C); v_{max}/cm^{-1} 3350 (N-H), 1697 (C=O), 1520 (N-H), 1169 (C-O); HRMS (Found $(M+H)^+$ 462.3565. Calcd. for $C_{28}H_{47}O_4N$: $(M+H)^+$ 462.3578.).

tert-butyl (5-(4-decylphenethyl)-2,2-dimethyl-1,3-dioxan-5-yl)carbamate **8d**

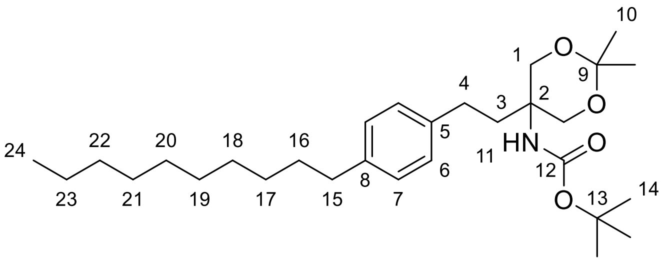

Compound **6** (0.40 g, 0.87 mmol) and dec-1-yne (0.24 mL, 1.31 mmol) were reacted following general method B to give compound **7d** (0.30 g), which was used in the next reaction step without further purification and characterisation following general method C. Compound **8d** was gained as white solid (0.20 g, 48%). Mp: 48-51 °C. ^{1}H NMR (400 MHz, $CDCl_3$) δ_H 7.11 (4H, m, 6, 7-H), 5.00 (1H, br s, 11-H), 3.92 (2H, d, *J*=11.6 Hz, 1a-H), 3.70 (2H, d, *J*=12.0 Hz, 1b-H), 2.56 (4H, m, 4, 15-H), 1.99 (2H, m, 3-H), 1.58 (2H, quint, *J*=7.2 Hz, 16-H), 1.50 (9H, s, 14-H), 1.46 (3H, s, 10a-H), 1.44 (3H, s, 10b-H), 1.28 (14H, m, 17 – 23-H), 0.90 (3H, t, *J*=6.8 Hz, 24-H); ^{13}C NMR (101 MHz, $CDCl_3$, mixture of rotamers – mr: major rotamer) δ_c 156.5 (12-C), 140.7, 140.5^{mr} (8-C), 139.1^{mr}, 138.8 (5-C), 128.6, 128.5^{mr} (7-C), 128.2^{mr}, 128.1 (6-C), 98.4 (9-C), 80.2 (13-C), 66.6^{mr}, 66.4 (1-C), 59.4, 51.7^{mr} (2-C), 35.6 (15-C), 35.4^{mr}, 33.7 (3-C), 31.9 (23-C), 31.6 (16-C), 29.6 (17-C), 29.6 (18-C), 29.5 (19-C), 29.4 (20-C), 29.4 (21-C), 29.1, 28.6mr (4-C), 28.5mr, 28.4 (14-C), 27.5 (10a-C), 22.7 (22-C), 19.7 (10b-C), 14.1 (24-C); v_{max}/cm^{-1} 3347 (N-H), 1699 (C=O), 1516 (N-H), 1171 (C-O); HRMS (Found $(M+Na)^+$ 498.3563. Calcd. for $C_{29}H_{49}O_4N$: $(M+Na)^+$ 498.3554.).

2-amino-2-(4-hexylphenethyl)propane-1,3-diol **9a**[3]

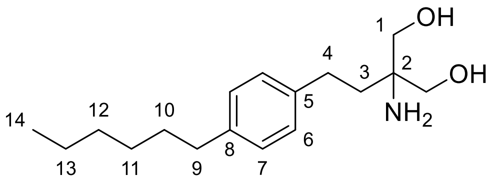

Compound **9a** was synthesized following general method D from compound **8a** (0.22 g, 0.54 mmol). The pure product was obtained as white solid (0.07 g, 46%). Mp: 109-111 °C. ^{1}H NMR (400 MHz, MeOD-d$_4$) δ_H 7.01 (2H, d, *J*=8.0 Hz, 6-H), 6.95 (2H, d, *J*=8.0 Hz 7-H), 3.42 (2H, d, *J*=10.8 Hz, 1a-H), 3.35 (2H, d, *J*=10.8 Hz, 1b-H), 2.50 (2H, m, 4-H), 2.44 (2H, t, *J*=7.6 Hz, 9-H), 1.55 (2H, m, 3-H), 1.47 (2H, m, 10-H), 1.20 (6H, m, 11 – 13-H), 0.79 (3H, t, *J*=6.8 Hz, 14-H); ^{13}C NMR (101 MHz, MeOD-d$_4$) δ_c 139.9 (8-C), 139.7 (5-C), 127.9 (7-C), 127.8 (6-C), 65.1 (1-C), 55.5 (2-C), 36.3 (3-C), 35.1 (9-C), 31.5 (12-C), 31.4 (10-C), 28.6 (4-C), 28.6 (11-C), 22.3 (13-C), 13.0 (14-C); v_{max}/cm^{-1} 3358 (O-H). 3298 (N-H), 1016 (C-O); HRMS (Found (M+H)$^+$ 280.2260. Calcd. for $C_{17}H_{29}O_2N$: (M+H)$^+$ 280.2271.).

2-amino-2-(4-heptylphenethyl)propane-1,3-diol **9b**[4]

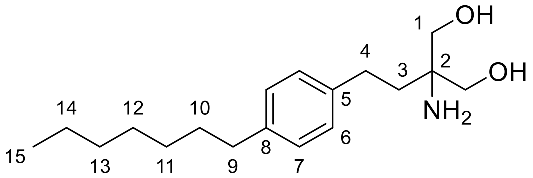

Compound **9b** was synthesized following general method D from compound **8b** (0.20 g, 0.46 mmol). The pure product was obtained as white solid (0.11 g, 85%). Mp: 114-117 °C. ^{1}H NMR (400 MHz, MeOD-d$_4$) δ_H 7.01 (2H, d, *J*=8.0 Hz, 6-H), 6.95 (2H, d, *J*=8.0 Hz 7-H), 3.42 (2H, d, *J*=10.8 Hz, 1a-H), 3.35 (2H, d, *J*=10.8 Hz, 1b-H), 2.51 (2H, m, 4-H), 2.45 (2H, t, *J*=7.6 Hz, 9-H), 1.56 (2H, m, 3-H), 1.47 (2H, m, 10-H), 1.21 (8H, m, 11 – 14-H), 0.79 (3H, t, *J*=6.8 Hz, 15-H); ^{13}C NMR (101 MHz, MeOD-d$_4$) δ_c 139.9 (8-C), 139.8 (5-C), 128.0 (7-C), 127.8 (6-C), 65.0 (1-C), 55.5 (2-C), 36.2 (3-C), 35.1 (9-C), 31.5 (13-C), 31.4 (10-C), 28.9 (11-C), 28.9 (12-C), 28.6 (4-C), 22.3 (14-C), 13.0 (15-C); v_{max}/cm^{-1} 3354 (O-H). 3294 (N-H), 1016 (C-O); HRMS (Found (M+H)$^+$ 294.2418. Calcd. for $C_{18}H_{31}O_2N$: (M+H)$^+$ 294.2428.).

2-amino-2-(4-octylphenethyl)propane-1,3-diol **9c**[4]

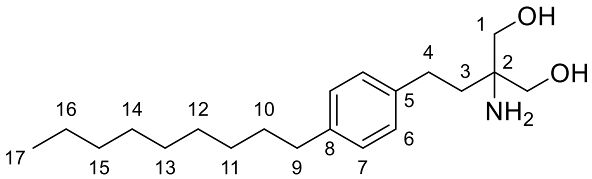

Compound **9c** was synthesized following general method D from compound **8c** (0.23 g, 0.50 mmol). The pure product was obtained as white solid (0.06 g, 36%). Mp: 107-109 °C. ^{1}H NMR (400 MHz, MeOD-d$_4$) δ_H 7.01 (2H, d, *J*=8.0 Hz, 6-H), 6.95 (2H, d, *J*=8.0 Hz 7-H), 3.42 (2H, d, *J*=10.8 Hz, 1a-H), 3.35 (2H, d, *J*=11.2 Hz, 1b-H), 2.51 (2H, m, 4-H), 2.45 (2H, t, *J*=7.6 Hz, 9-H), 1.55 (2H, m, 3-H), 1.47 (2H, m, 10-H), 1.18 (12H, m, 11 – 16-H), 0.79 (3H, t, *J*=6.8 Hz, 17-H); ^{13}C NMR (101 MHz, MeOD-d$_4$) δ_c 139.9 (8-C), 139.8 (5-C), 128.0 (7-C), 127.8 (6-C), 65.0 (1-C), 55.5 (2-C), 36.3 (3-C), 35.1 (9-C), 31.7 (15-C), 31.4 (10-C), 29.3 (12-C), 29.2 (13-C), 29.0 (14-C), 28.9 (11-C), 28.6 (4-C), 22.3 (16-C), 13.1 (17-C); v_{max}/cm^{-1} 3354 (O-H). 3291 (N-H), 1016 (C-O); HRMS (Found (M+H)$^+$ 322.2733. Calcd. for $C_{20}H_{35}O_2N$: (M+H)$^+$ 322.2741.).

2-amino-2-(4-decylphenethyl)propane-1,3-diol **9d**[4]

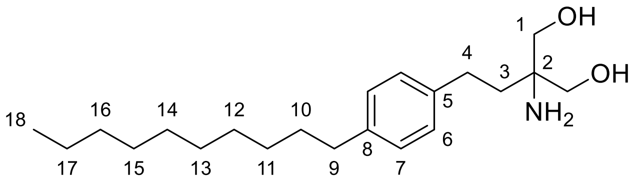

Compound **9d** was synthesized following general method D from compound **8d** (0.18 g, 0.38 mmol). The pure product was obtained as white solid (0.10 g, 81%). Mp: 105-107 °C. ^{1}H NMR (400 MHz, MeOD-d$_4$) δ_H 7.01 (2H, d, *J*=8.0 Hz, 6-H), 6.95 (2H, d, *J*=8.0 Hz 7-H), 3.42 (2H, d, *J*=10.8 Hz, 1a-H), 3.35 (2H, d, *J*=11.2 Hz, 1b-H), 2.51 (2H, m, 4-H), 2.45 (2H, t, *J*=7.6 Hz, 9-H), 1.55 (2H, m, 3-H), 1.47 (2H, m, 10-H), 1.18 (12H, m, 11 – 17-H), 0.80 (3H, t, *J*=6.8 Hz, 18-H); ^{13}C NMR (101 MHz, MeOD-d$_4$) δ_c 139.9 (8-C), 139.8 (5-C), 128.0 (7-C), 127.8 (6-C), 65.0 (1-C), 55.5 (2-C), 36.3 (3-C), 35.1 (9-C), 31.7 (16-C), 31.4 (10-C), 29.3 (12-C), 29.3 (13-C), 29.2 (14-C), 29.1 (15-C), 28.9 (11-C), 28.6 (4-C), 22.3 (17-C), 13.1 (18-C); v_{max}/cm^{-1} 3316 (N-H), 1016 (C-O); HRMS (Found (M+H)$^+$ 336.2886. Calcd. for $C_{21}H_{37}O_2N$: (M+H)$^+$ 336.2897.).

tert-butyl (2,2-dimethyl-5-(4-nitrostyryl)-1,3-dioxan-5-yl)carbamate **11**

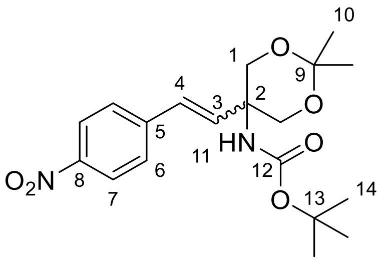

Phosphonium salt **10** (6.64 g, 13.9 mmol) was used in the synthesis of compound **11** following General method A. Compound **6** with Z isomer impurity (19%) was gained as salmon coloured solid (4.29 g, 98%). Mp: 112-115 °C; ^{1}H NMR (400 MHz, CDCl$_3$) δ_H 8.20 (2H, d, *J*=8.8 Hz, 7-C), 7.51 (2H, d, *J*=8.8 Hz, 6-C), 6.63 (1H, d, *J*=16.4 Hz, 4-H), 6.44 (1H, d, *J*=16.4 Hz, 3-H), 5.27 (1H, br s, 11-H), 3.97 (4H, m, 1-H), 1.52 (3H, s, 10a-H), 1.50 (3H, s, 10b-H), 1.47 (9H, s, 14-H); ^{13}C NMR (101 MHz, CDCl$_3$) δ_c 154.8 (12-C), 147.1 (8-C), 143.0 (5-C), 133.7 (3-C), 128.5 (4-C), 127.0 (6-C), 124.0 (7-C), 98.5 (9-C), 80.8 (13-C), 66.0 (1-C), 53.2 (2-C), 28.4 (14-C), 27.5 (10a-C), 19.6 (10b-C); v_{max}/cm^{-1} 3319 (N-H), 1707 (C=O), 1514 (N-H), 1166 (C-O); HRMS (Found (M+Na)$^+$ 401.1691. Calcd. for $C_{19}H_{26}O_6N_2$: (M+Na)$^+$ 401.1683.).

tert-butyl (5-(4-aminophenethyl)-2,2-dimethyl-1,3-dioxan-5-yl)carbamate **12**

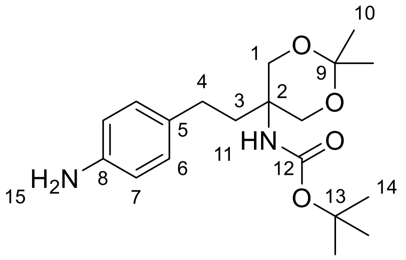

To the solution of compound **11** (2.80 g, 8.00 mmol) in methanol (50 mL) 10% palladium on carbon (0.28 g) was added portion wise, and the mixture was stirred under 30 psi hydrogen atmosphere overnight at room temperature. After the reaction was complete, it was filtered through celite and the filtrate was evaporated under reduced pressure to give the pure product as white solid (2.16 g, 83%). Mp: 158-160 °C; ^{1}H NMR (400 MHz, CDCl$_3$) δ_H 6.98 (2H, d, *J*=8.0 Hz, 6-H), 6.63 (2H, d, *J*=8.4 Hz, 7-H), 4.99 (1H, br s, 11-H), 3.90 (2H, d, *J*=11.6 Hz, 1a-H),

3.67 (2H, d, *J*=12.0 Hz, 1b-H), 3.58 (2H, br s, 15-H), 2.48 (2H, m, 4-H), 1.94 (2H, m, 3-H), 1.49 (9H, s, 14-H), 1.45 (10a-H), 1.43 (10b-H); ^{13}C NMR (101 MHz, $CDCl_3$) δ_c 154.9 (12-C), 144.3 (8-C), 132.0 (5-C), 129.1 (6-C), 115.3 (7-C), 98.3 (9-C), 79.3 (13-C), 66.4 (1-C), 51.7 (2-C), 33.9 (3-C), 28.5 (14-C), 28.2 (4-C), 27.5 (10a-C), 19.7 (10b-C); ν_{max}/cm^{-1} 3364 (N-H), 3319 (N-H), 1699 (C=O), 1518 (N-H), 1153 (C-O); HRMS (Found $(M+H)^+$ 351.2271. Calcd. for $C_{19}H_{30}O_4N_2$: $(M+H)^+$ 351.2278.).

tert-butyl (2,2-dimethyl-5-(4-(oct-1-yn-1-yl)phenethyl)-1,3-dioxan-5-yl)carbamate **14**[5]

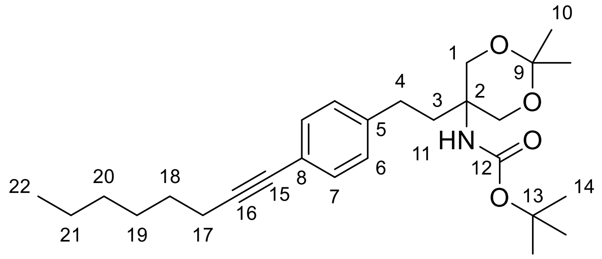

To the solution of compound **12** (2.80, 8.00 mmol) in toluene iodine (1.21 g, 4.80 mmol) and then tertbutylnitrite (0.91 g, 8.80 mmol) was added at 0 °C. The reaction was stirred overnight at room temperature, and after the reaction was complete it was quenched with water (150 mL). The aqueous layer was extracted with ethyl acetate (2 x 150 mL), the combined organic phases were dried over $MgSO_4$ and the solvent was removed under reduced pressure. The crude was purified by flash chromatography (eluent: hexane – ethyl acetate) to give the product as a salmon coloured solid (1.68 g, 46%). Part of this compound **13** (1.00 g, 2.17 mmol) was used in the next reaction step without further purification and characterisation following general method B to give the product as light brown solid (0.66 g, 69%). Mp: 57 -59 °C; ^{1}H NMR (400 MHz, $CDCl_3$) δ_H 7.31 (2H, d, *J*=8.4 Hz, 7-H), 7.11 (2H, d, *J*=8.4 Hz, 8-H), 5.00 (1H, br s, 11-H), 3.90 (2H, d, *J*=11.6 Hz, 1a-H), 3.68 (2H, d, *J*=12.0 Hz, 1b-H), 2.57 (2H, m, 4-H), 2.41 (2H, t, *J*=7.2 Hz, 17-H), 1.97 (2H, m, 3-H), 1.61 (2H, quint, *J*=7.2 Hz, 18-H), 1.51 (2H, m, 19-H), 1.50 (9H, s, 14-H), 1.45 (3H, s, 10a-H), 1.43 (3H, s, 10b-H), 1.34 (4H, m, 20, 21-H), 0.92 (3H, t, *J*=6.8 Hz, 22-H); ^{13}C NMR (101 MHz, $CDCl_3$, mixture of rotamers – mr: major rotamer) δ_c 156.4^{mr}, 154.9 (12-C), 141.5^{mr}, 141.2 (5-C), 131.7, 131.6^{mr} (7-C), 128.3^{mr}, 128.2 (6-C), 121.8, 121.7^{mr} (8-C), 98.4 (9-C), 90.0, 89.9^{mr} (15-C), 80.5^{mr}, 80.2 (16-C), 66.5, 66.3^{mr} (1-C), 59.2, 51.7mr (2-C), 34.9, 33.4^{mr} (3-C), 31.4 (20-C), 29.5, 29.0^{mr} (4-C), 28.8 (18-C), 28.6 (19-C), 28.4mr, 28.3 (14-C), 27.4 (10a-C), 22.6 (21-C), 19.7 (10b-C), 19.4 (17-C), 14.1 (22-C); ν_{max}/cm^{-1} 3343 (N-H), 1697 (C=O), 1522 (N-H), 1167 (C-O); HRMS (Found $(M+H)^+$ 444.3114. Calcd. for $C_{27}H_{41}O_4N$: $(M+H)^+$ 444.3108.).

tert-butyl (2,2-dimethyl-5-(4-(Z-oct-1-ene-1-yl)phenethyl)-1,3-dioxan-5-yl)carbamate **15**[6]

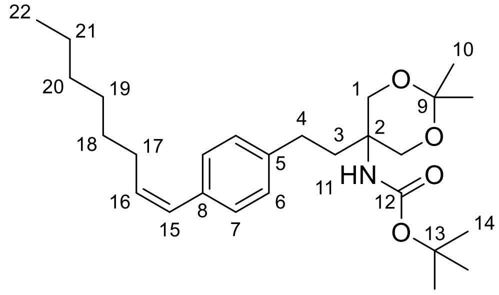

To the solution of compound **14** (0.53 g, 1.19 mmol) in ethyl acetate (12 mL) Lindlar catalyst (0.44 g) and quinoline (0.12 mL). This mixture was stirred under atmospheric H_2 atmosphere overnight. After the reaction was complete, it was filtered through celite, and it was evaporated under reduced pressure. The crude was purified by column chromatography (eluent: hexane

– ethyl acetate) to give the product as white solid (0.35 g, 66%, Z isomer: 89%). Mp: 43 - 46 °C; ^{1}H NMR (400 MHz, $CDCl_3$) δ_H 7.21 (2H, d, *J*=8.4 Hz, 7-H), 7.16 (2H, d, *J*=8.0 Hz, 6-H), 6.38 (1H, d, *J*=11.6 Hz, 15-H), 5.64 (1H, dt, *J*=11.6 Hz, *J*=7.2 Hz, 16-H), 5.02 (1H, br s, 11-H), 3.93 (2H, d, *J*=12.0 Hz, 1a-H), 3.71 (2H, d, *J*=11.6 Hz, 1b-H), 2.58 (2H, m, 4-H), 2.33 (2H, quartd, *J*=7.2 Hz, *J*=2.0 Hz, 17-H), 2.01 (2H, m, 3-H), 1.50 (9H, s, 14-H), 1.46 (3H, s, 10a-H), 1.48 (2H, m, 18-H), 1.44 (3H, s, 10b-H), 1.30 (6H, m, 19 – 21-H), 0.90 (3H, t, J=7.2 Hz, 22-H); ^{13}C NMR (101 MHz, $CDCl_3$) δ_c 154.9 (12-C), 140.2 (5-C), 135.6 (8-C), 132.8 (16-C), 128.9 (7-C), 128.4 (15-C), 128.1 (6-C), 98.4 (9-C), 79.3 (13-C), 66.4 (1-C), 51.7 (2-C), 33.6 (3-C), 31.8 (20-C), 30.0 (18-C), 29.1 (19-C), 28.8 (4-C), 28.7 (17-C), 28.5 (14-C), 27.5 (10a-C), 22.7 (21-C), 19.7 (10b-C), 14.1 (22-C); v_{max}/cm^{-1} 3347 (N-H), 1697 (C=O), 1521 (N-H), 1168 (C-O); HRMS (Found $(M+H)^+$ 446.3251. Calcd. for $C_{27}H_{43}O_4N$: $(M+H)^+$ 446.3265.).

2-amino-2-(4-(Z-oct-1-en-1-yl)phenethyl)propane-1,3-diol **16**[6]

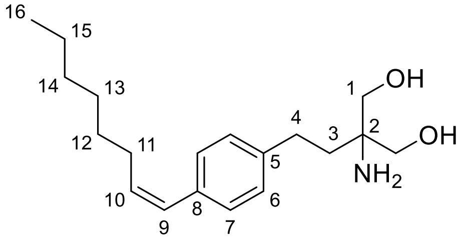

Compound **16** was synthesized following general method D from compound **15** (0.32 g, 0.72 mmol). The pure product was obtained as white solid (0.12 g, 54%, Z isomer: 90%). Mp: 96 - 98 °C. ^{1}H NMR (400 MHz, MeOD-d_4) δ_H 7.08 (2H, d, *J*=8.8 Hz, 6-H), 7.05 (2H, d, *J*=8.4 Hz, 7-H), 6.26 (1H, d, *J*=11.6 Hz, 9-H), 5.50 (1H, dt, *J*=11.6 Hz, *J*=7.2 Hz, 10-H), 3.42 (2H, d, *J*=10.8 Hz, 1a-H), 3.36 (2H, d, *J*=10.8 Hz, 1b-H), 2.54 (2H, m, 4-H), 2.21 (2H, quartd, *J*=8.0 Hz, *J*=2.0 Hz, 11-H), 1.57 (2H, m, 3-H), 1.36 (2H, quint, *J*=7.2 Hz, 12-H), 1.18 (6H, m, 13-15-H), 0.78 (3H, t, *J*=6.8 Hz, 16-H); ^{13}C NMR (101 MHz, MeOD-d_4) δ_c 141.0 (5-C), 135.2 (8-C), 131.8 (10-C), 128.5 (9-C), 128.4 (7-C), 127.7 (6-C), 65.0 (1-C), 55.5 (2-C), 36.1 (3-C), 31.5 (14-C), 29.6 (12-C), 28.7 (4, 13-C), 28.2 (11-C), 22.3 (15-C), 13.0 (16-C); v_{max}/cm^{-1} 3323 (O-H, N-H), 1030 (C-O); HRMS (Found $(M+H)^+$ 306.2419. Calcd. for $C_{19}H_{31}O_2N$: $(M+H)^+$ 306.2428.).

Safety Statement

No unexpected or unusually high safety hazards were encountered.

NMR, IR and HRMS Spectra of Compounds

tert-Butyl-5-(hydroxymethyl)-2,2-dimethyl-1,3-dioxan-5-ylaminoformylate **2**

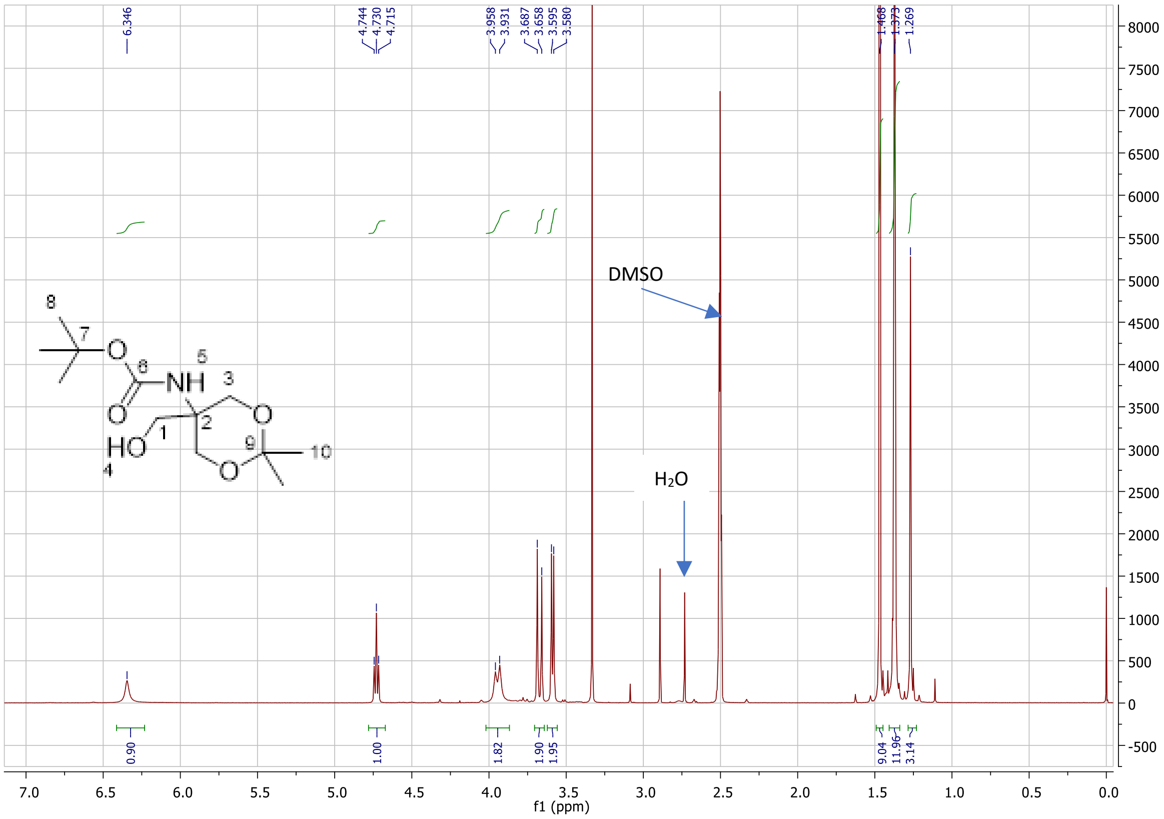

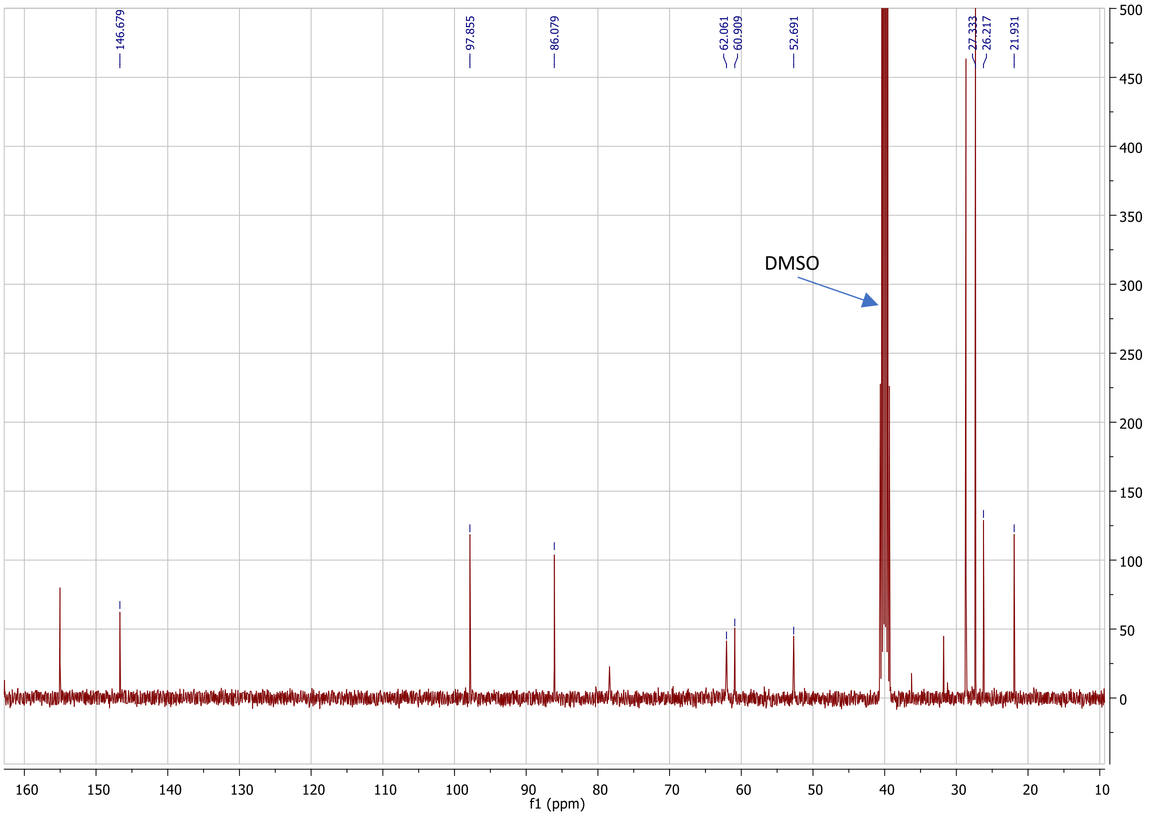

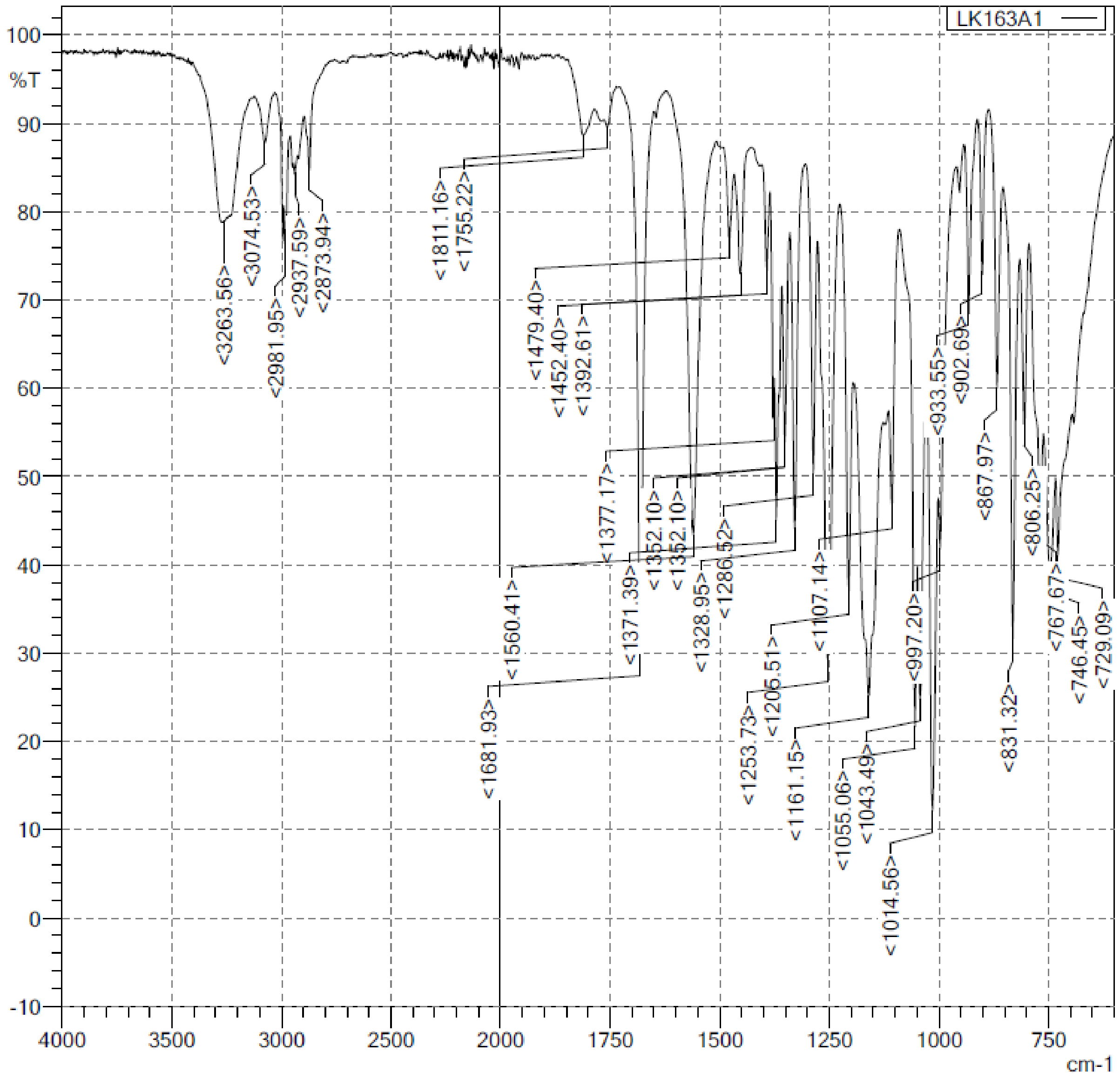

LK163A1
%T
cm-1
<3263.56>
<3074.53>
<2981.95>
<2937.59>
<2873.94>
<1811.16>
<1755.22>
<1681.93>
<1560.41>
<1479.40>
<1452.40>
<1392.61>
<1377.17>
<1371.39>
<1352.10>
<1352.10>
<1328.95>
<1286.52>
<1253.73>
<1205.51>
<1161.15>
<1107.14>
<1055.06>
<1043.49>
<1014.56>
<997.20>
<933.55>
<902.69>
<867.97>
<831.32>
<806.25>
<767.67>
<746.45>
<729.09>

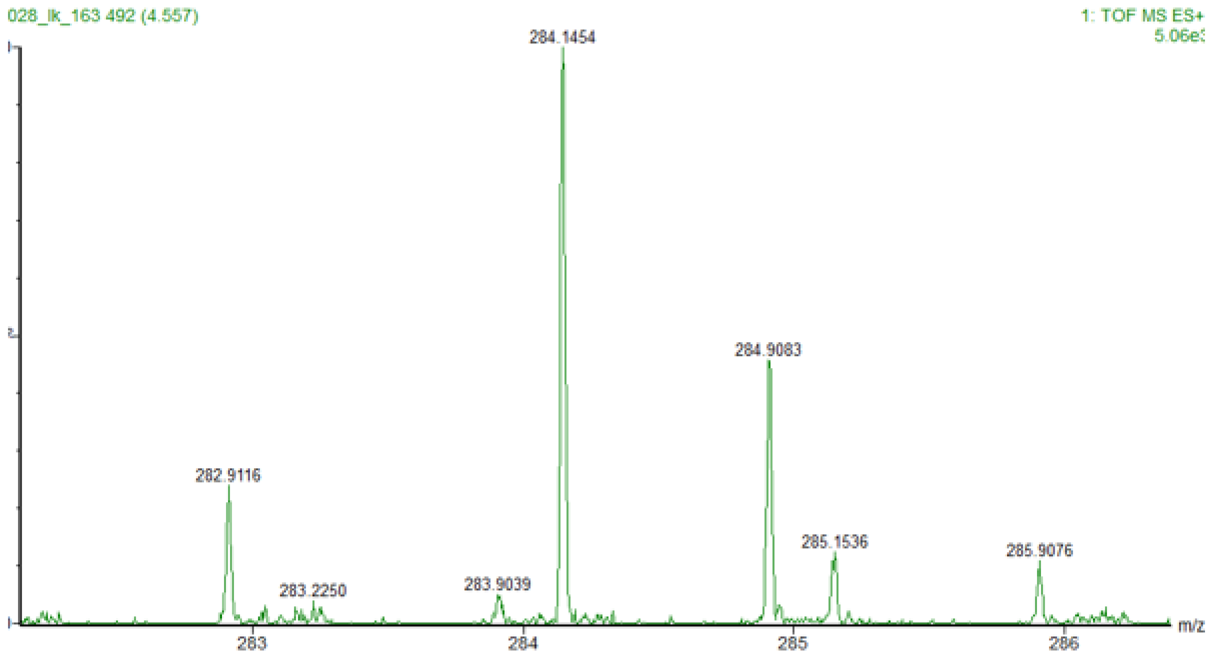

028_lk_163 492 (4.557)
1: TOF MS ES+
5.06e3
284.1454
284.9083
282.9116
283.2250
283.9039
285.1536
285.9076
m/z

tert-Butyl-5-formyl-2,2-dimethyl-1,3-dioxan-5-ylaminoformylate **3**

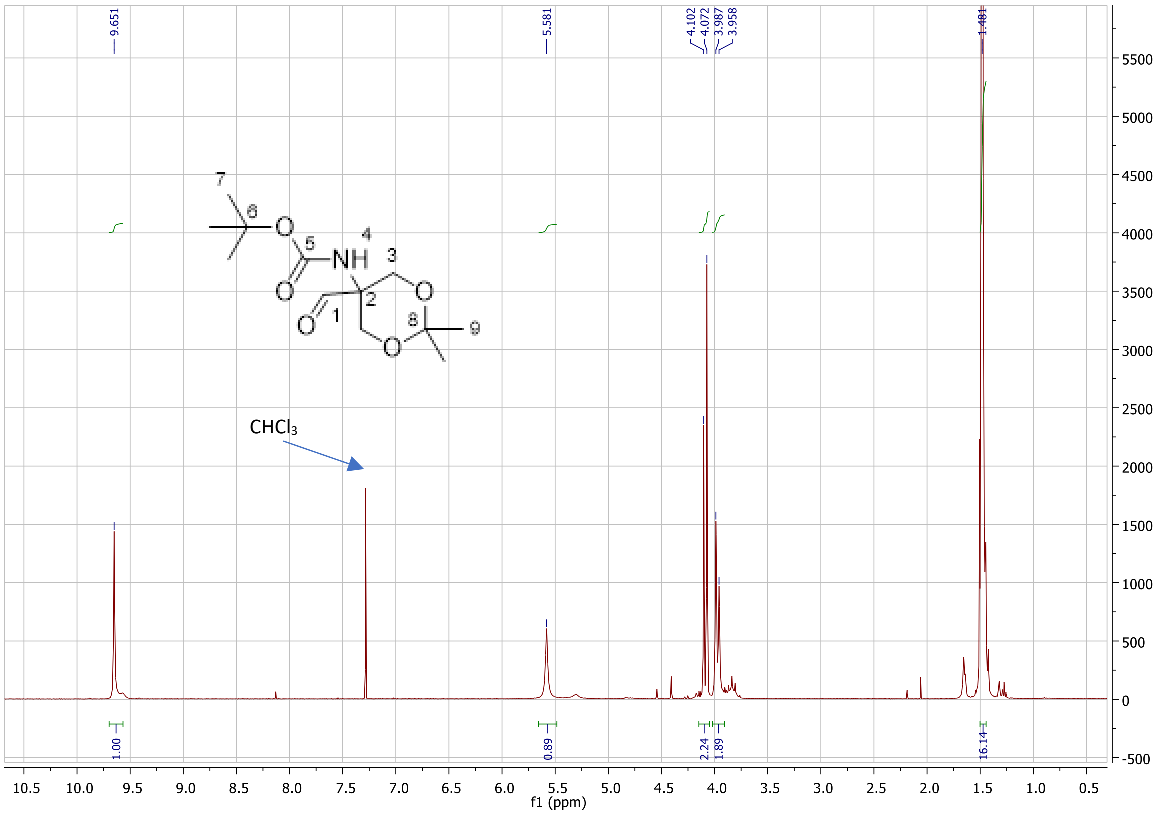

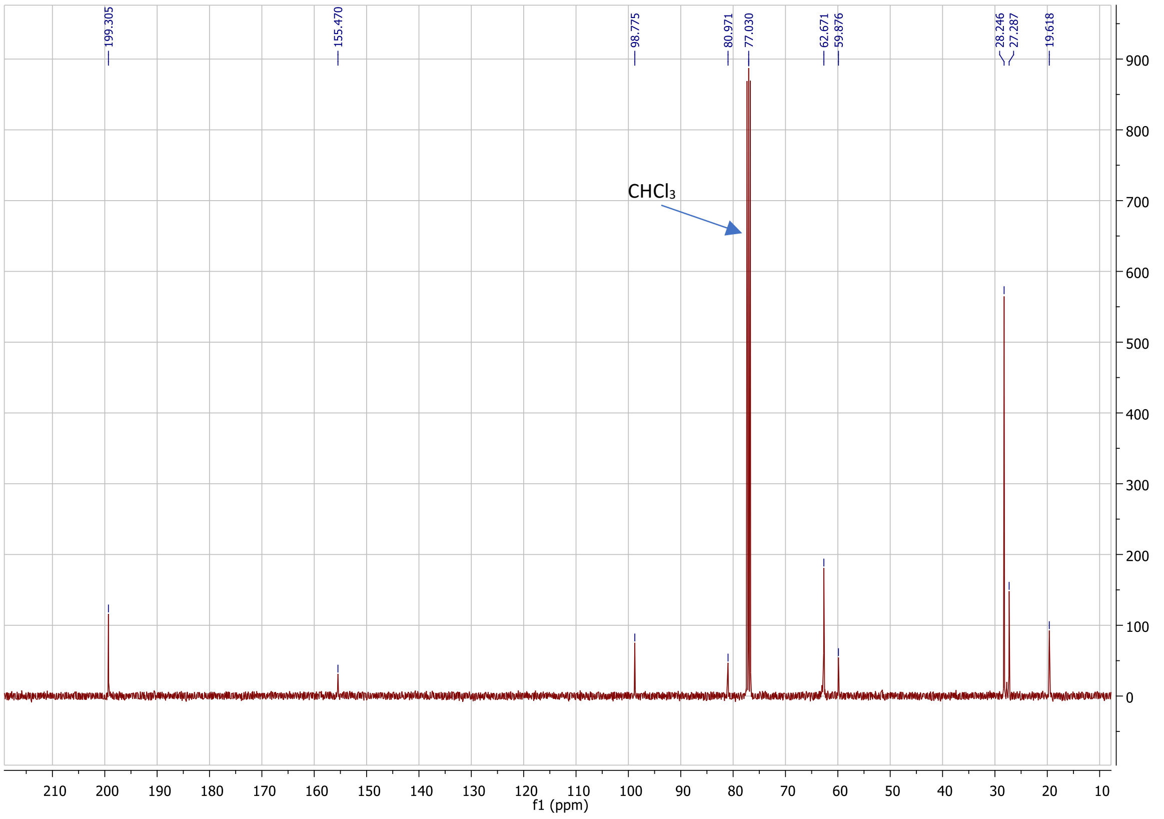

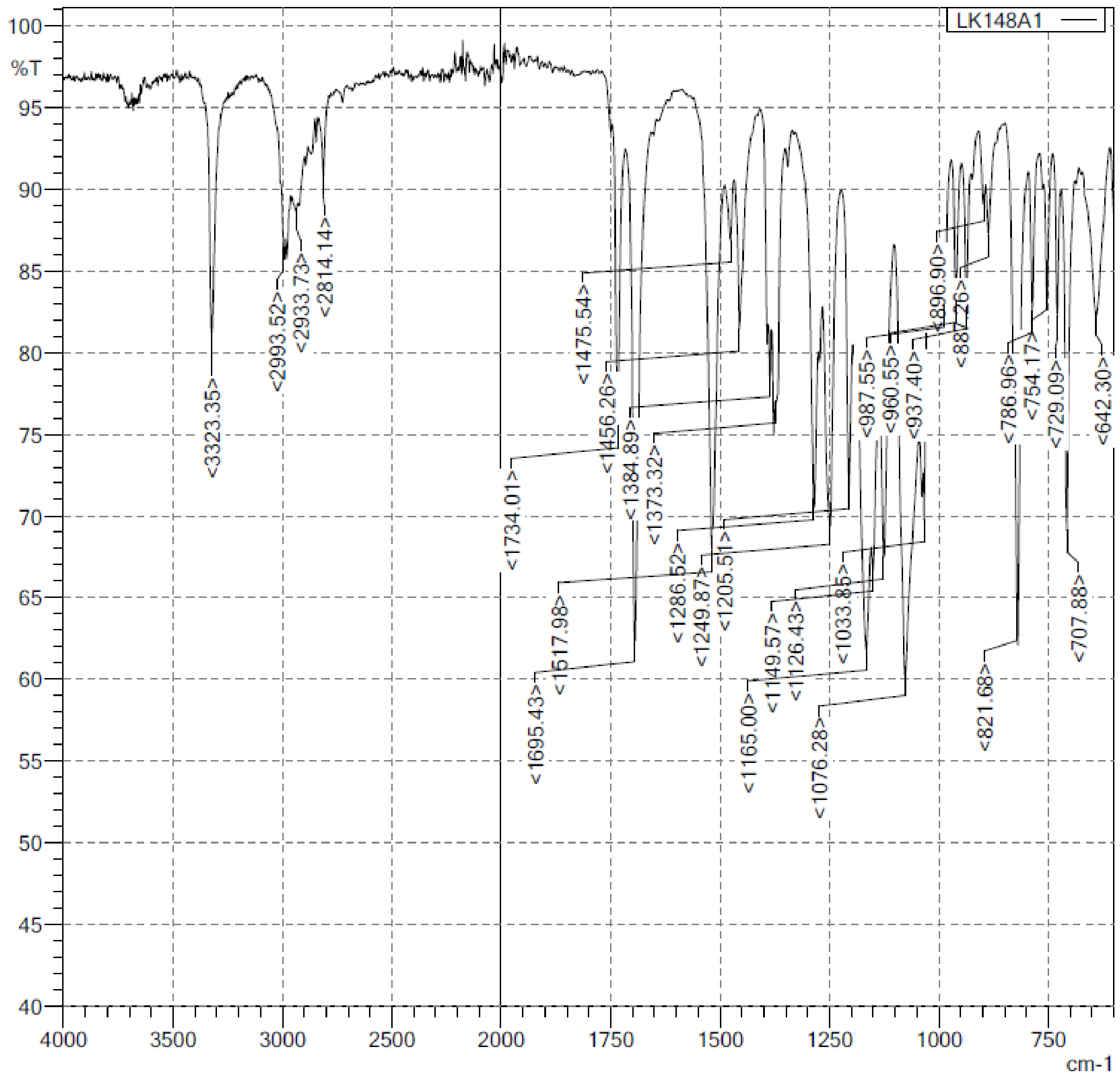
LK148A1
%T
cm-1
<3323.35>
<2993.52>
<2933.73>
<2814.14>
<1734.01>
<1695.43>
<1517.98>
<1475.54>
<1456.26>
<1384.89>
<1373.32>
<1286.52>
<1249.87>
<1205.51>
<1165.00>
<1149.57>
<1126.43>
<1076.28>
<1033.85>
<987.55>
<960.55>
<937.40>
<896.90>
<821.68>
<786.96>
<754.17>
<729.09>
<707.88>
<642.30>

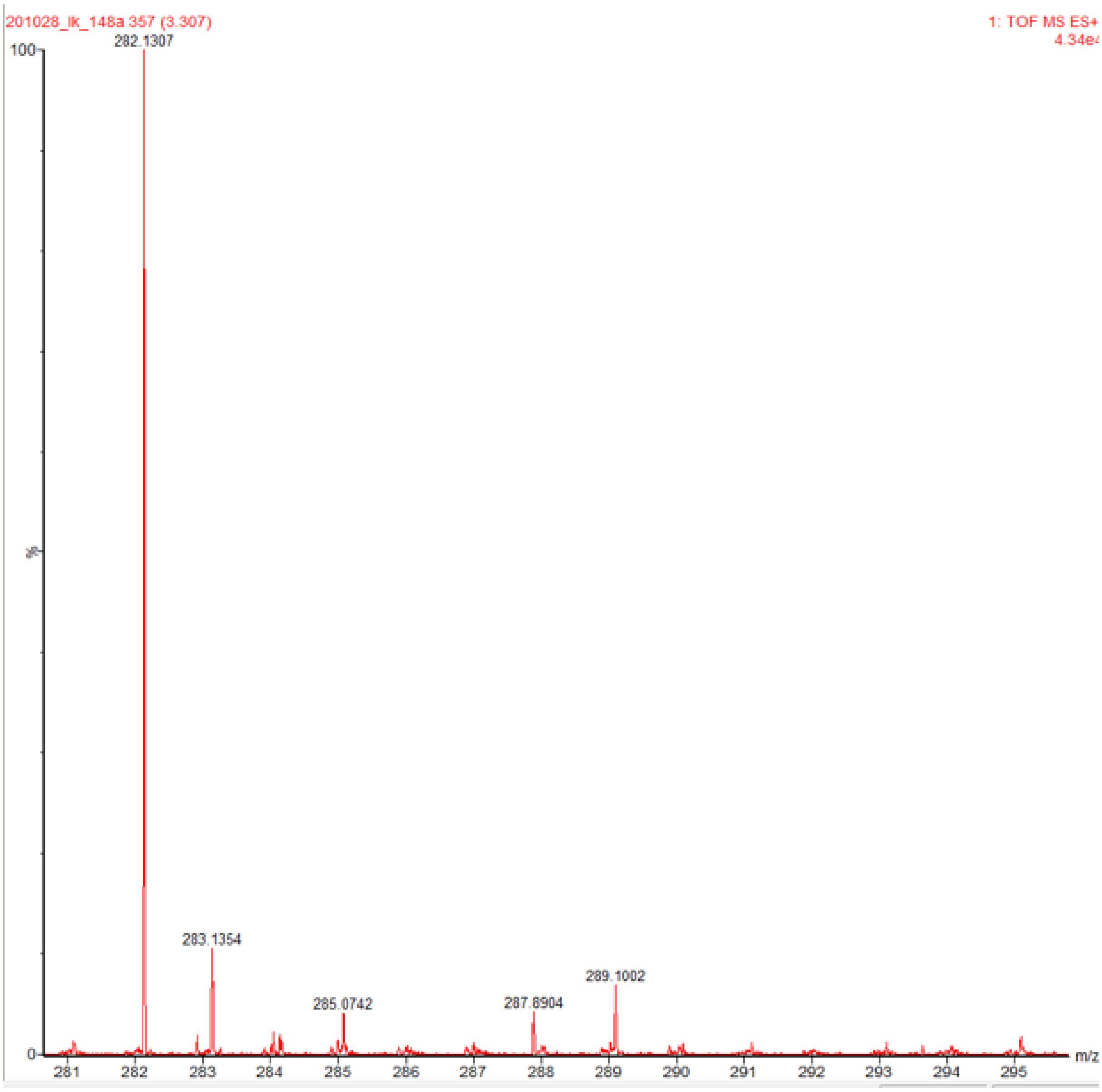

201028_lk_148a 357 (3.307)
1: TOF MS ES+
4.34e4
282.1307
283.1354
285.0742
287.8904
289.1002
100
%
0
281
282
283
284
285
286
287
288
289
290
291
292
293
294
295
m/z

5-[(*E*)-2-(*p*-Iodophenyl)ethenyl]-5-(*tert*-butoxycarbonylamino)-2,2-dimethyl-1,3-dioxane **6**

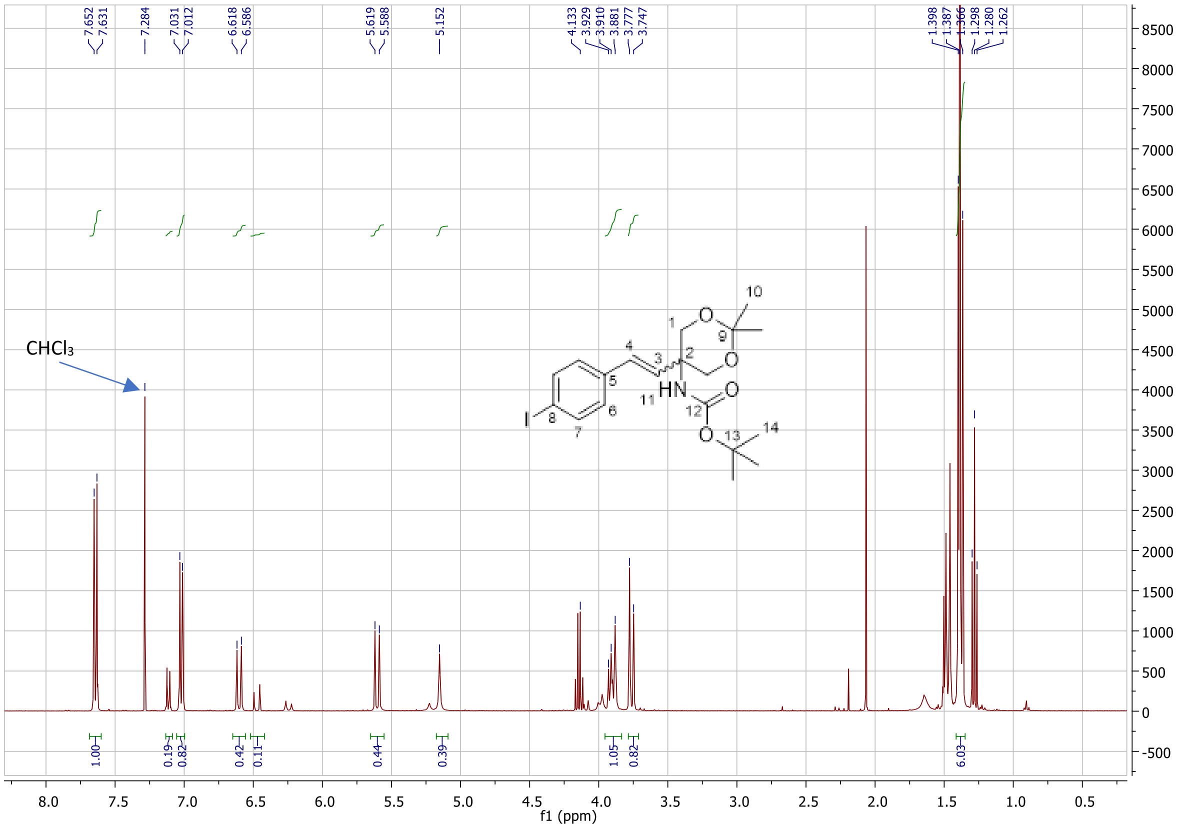

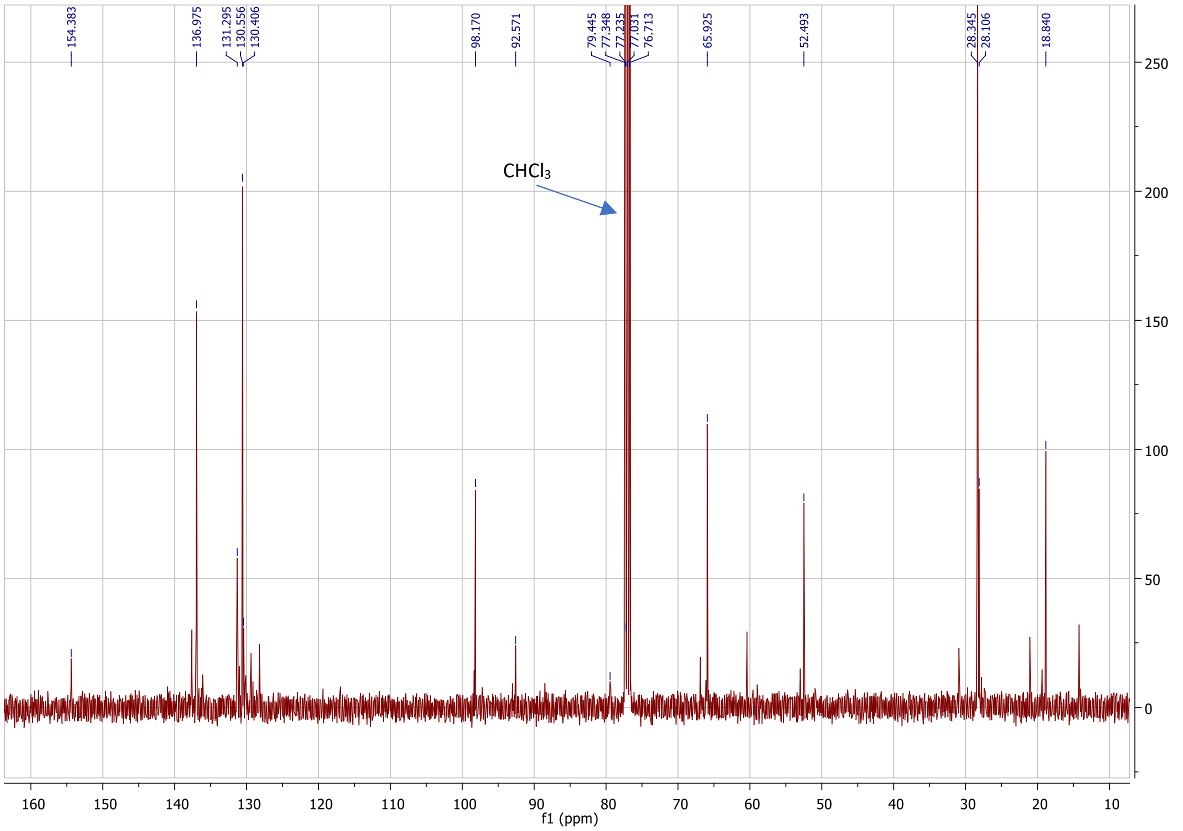

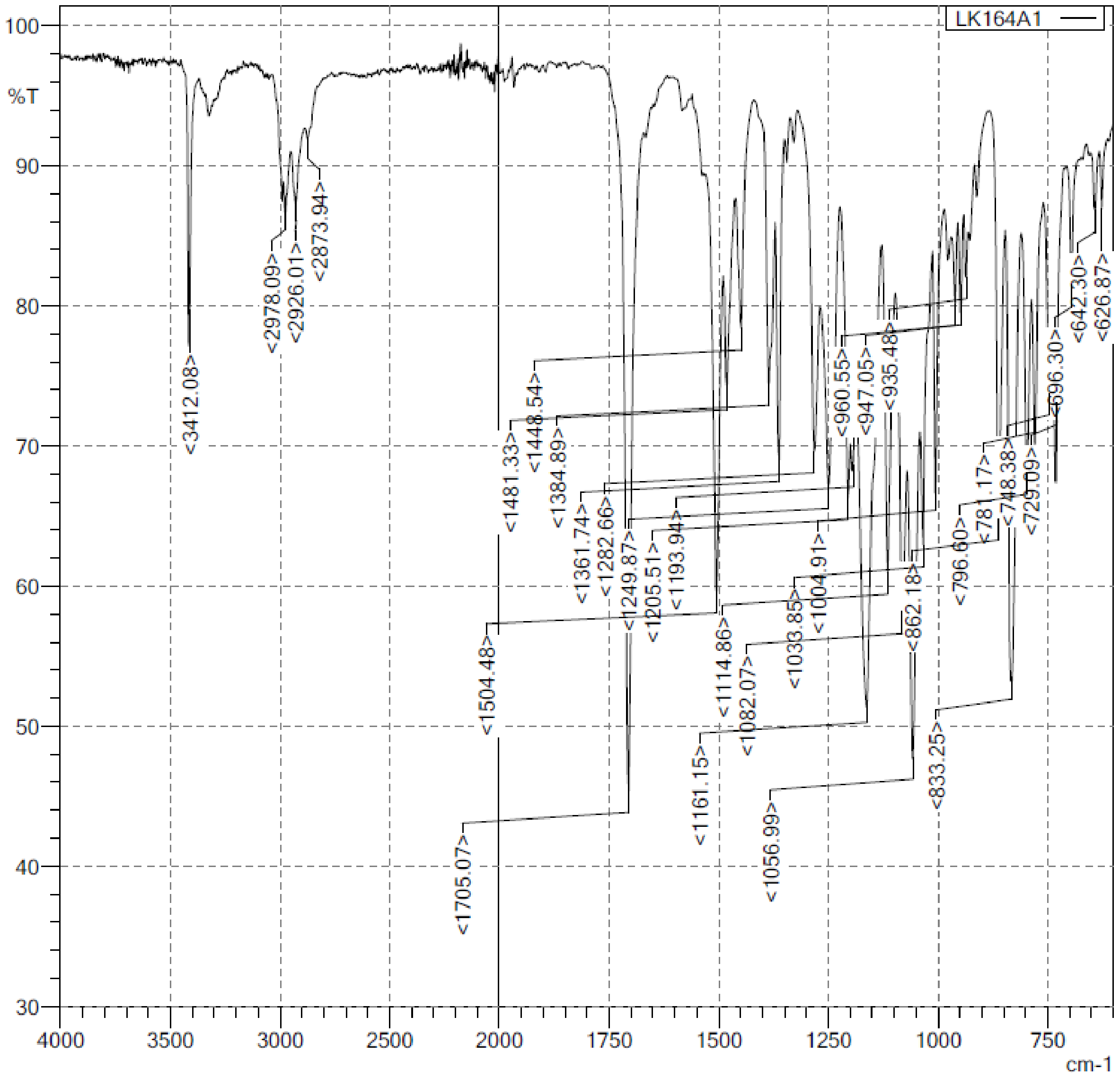
LK164A1
100
90
80
70
60
50
40
30
%T
4000
3500
3000
2500
2000
1750
1500
1250
1000
750
cm-1
<3412.08>
<2978.09>
<2926.01>
<2873.94>
<1705.07>
<1504.48>
<1481.33>
<1448.54>
<1384.89>
<1361.74>
<1282.66>
<1249.87>
<1205.51>
<1193.94>
<1161.15>
<1114.86>
<1082.07>
<1056.99>
<1033.85>
<1004.91>
<960.55>
<947.05>
<935.48>
<862.18>
<833.25>
<796.60>
<781.17>
<748.38>
<729.09>
<696.30>
<642.30>
<626.87>

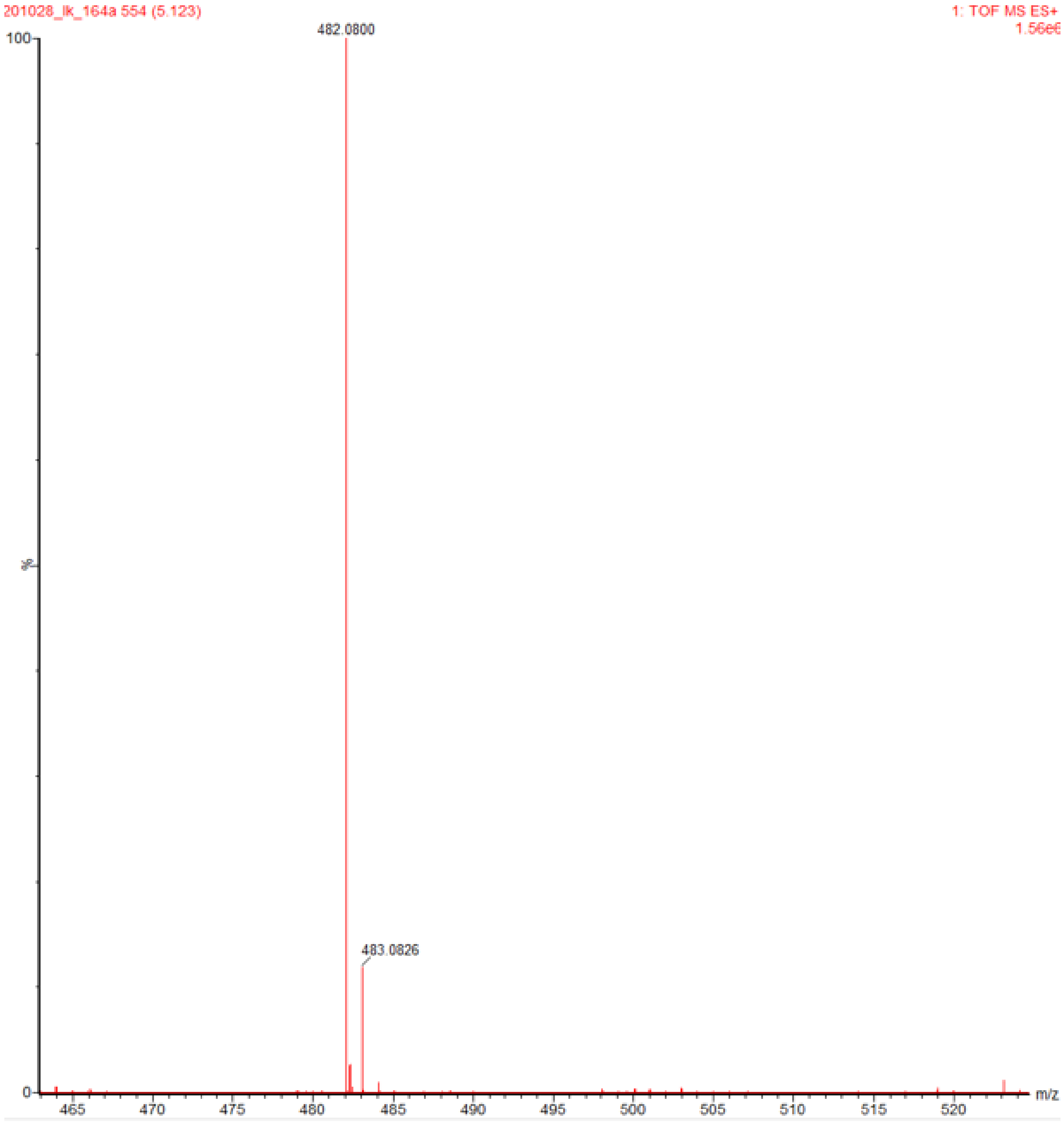
201028_lk_164a 554 (5.123)
1: TOF MS ES+
1.56e6
482.0800
483.0826
100
%
0
m/z
465
470
475
480
485
490
495
500
505
510
515
520

5-(*tert*-Butoxycarbonylamino)-5-[2-(*p*-hexylphenyl)ethyl]-2,2-dimethyl-1,3-dioxane **8a**

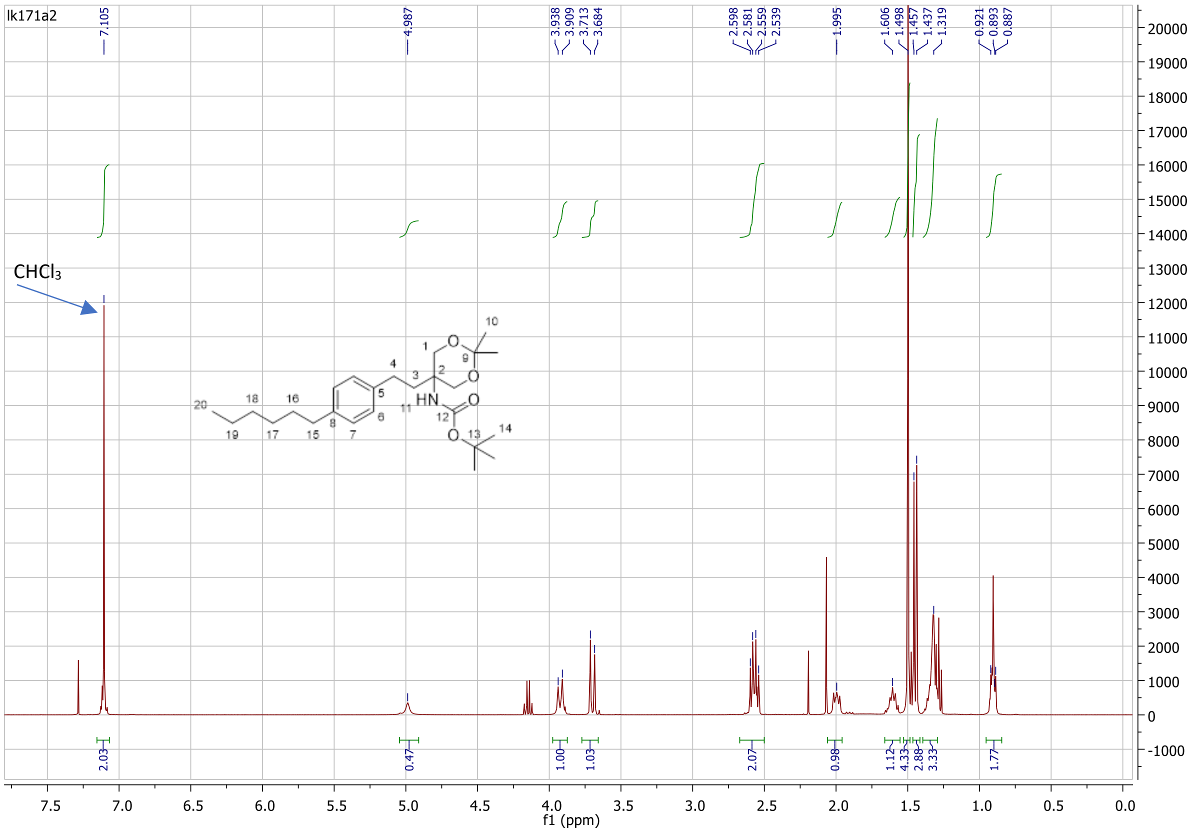

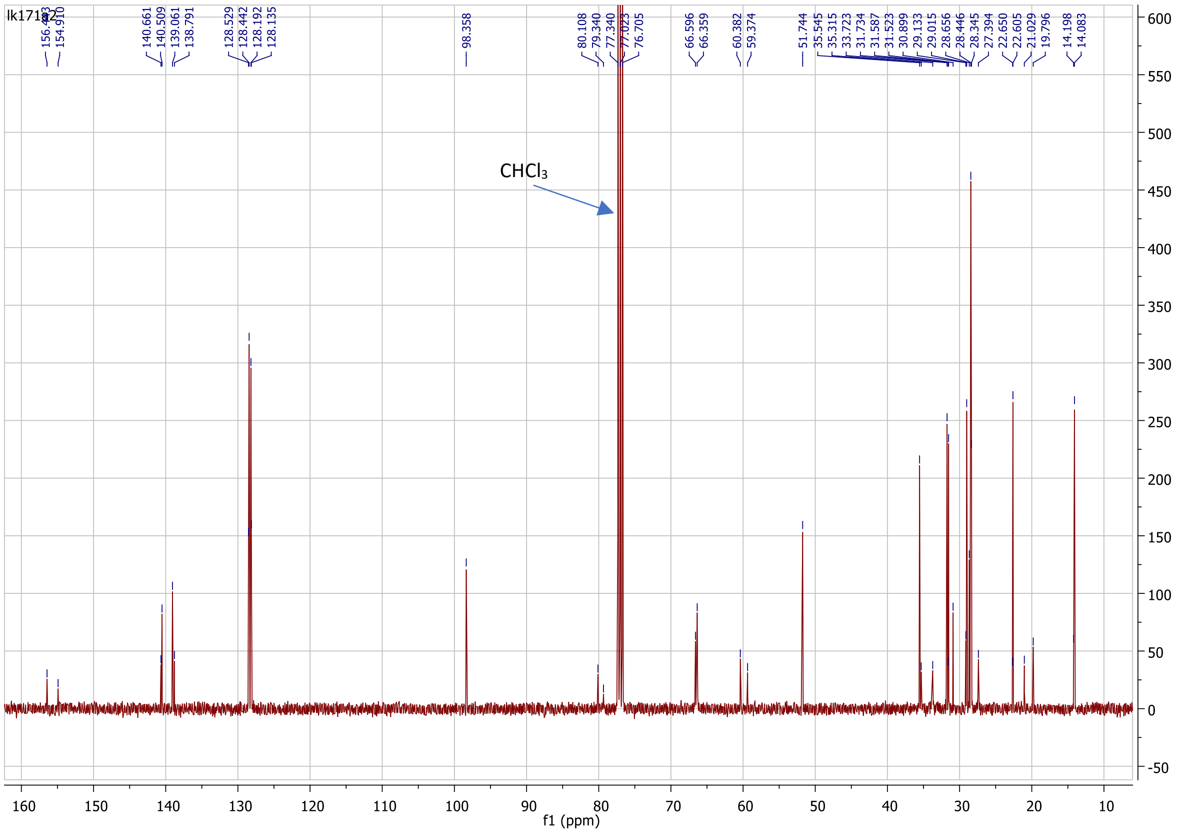

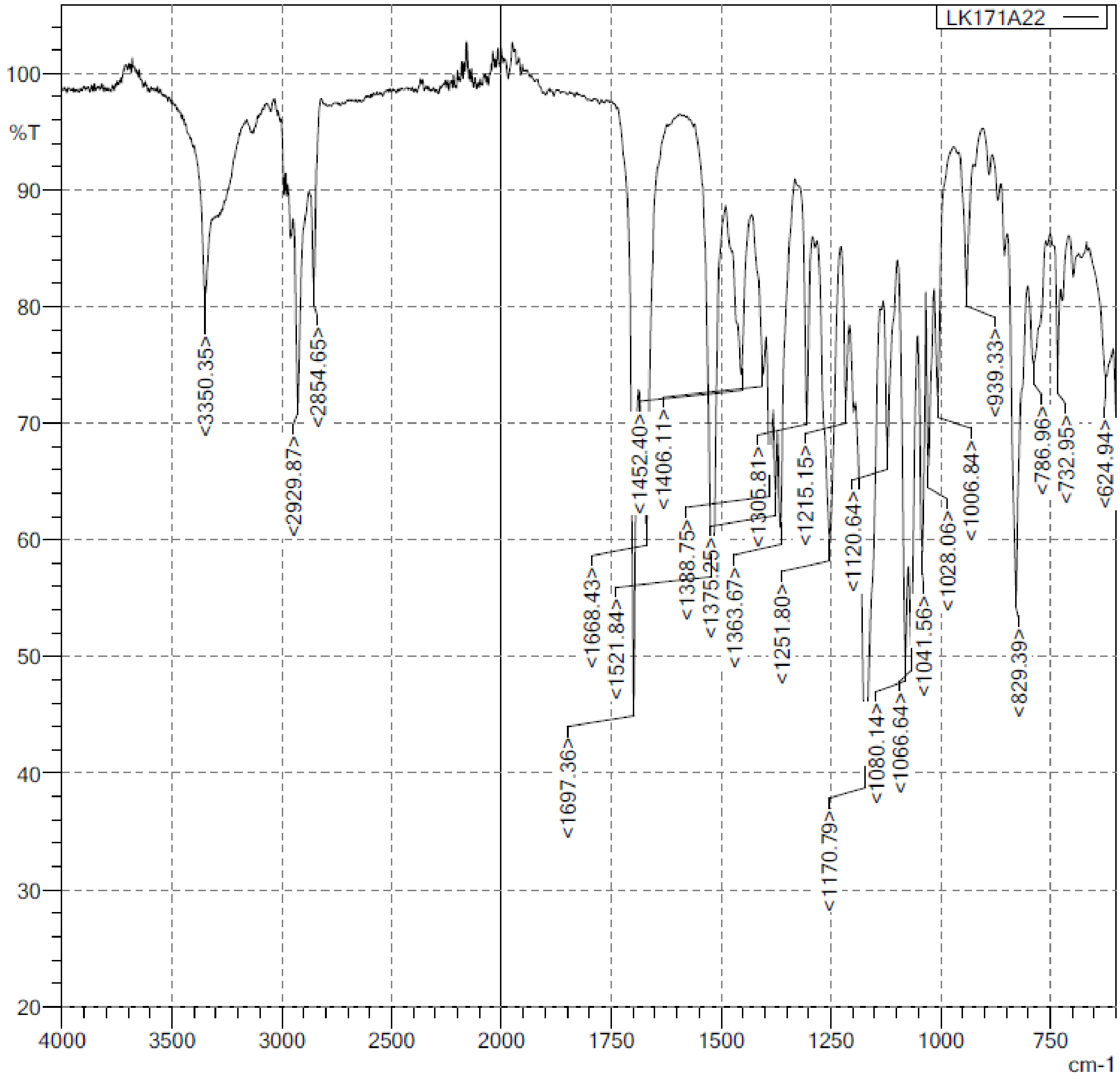

LK171A22
%T
100
90
80
70
60
50
40
30
20
4000
3500
3000
2500
2000
1750
1500
1250
1000
750
cm-1
<3350.35>
<2929.87>
<2854.65>
<1697.36>
<1668.43>
<1521.84>
<1452.40>
<1406.11>
<1388.75>
<1375.25>
<1363.67>
<1305.81>
<1251.80>
<1215.15>
<1170.79>
<1120.64>
<1080.14>
<1066.64>
<1041.56>
<1028.06>
<1006.84>
<939.33>
<829.39>
<786.96>
<732.95>
<624.94>

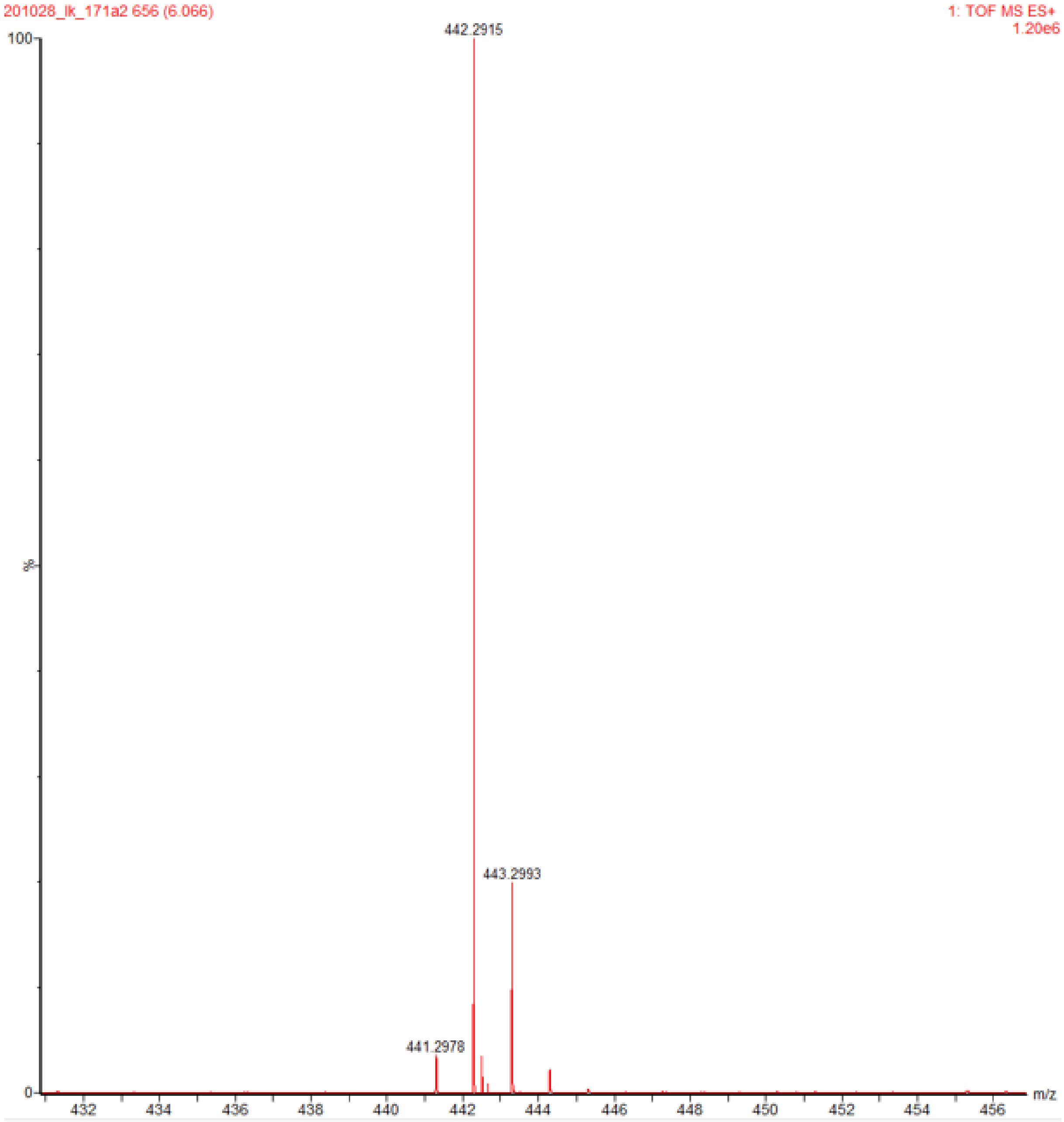
201028_lk_171a2 656 (6.066)
1: TOF MS ES+
1.20e6
100
%
0
442.2915
443.2993
441.2978
432
434
436
438
440
442
444
446
448
450
452
454
456
m/z

tert-butyl (5-(4-heptylphenethyl)-2,2-dimethyl-1,3-dioxan-5-yl)carbamate **8b**

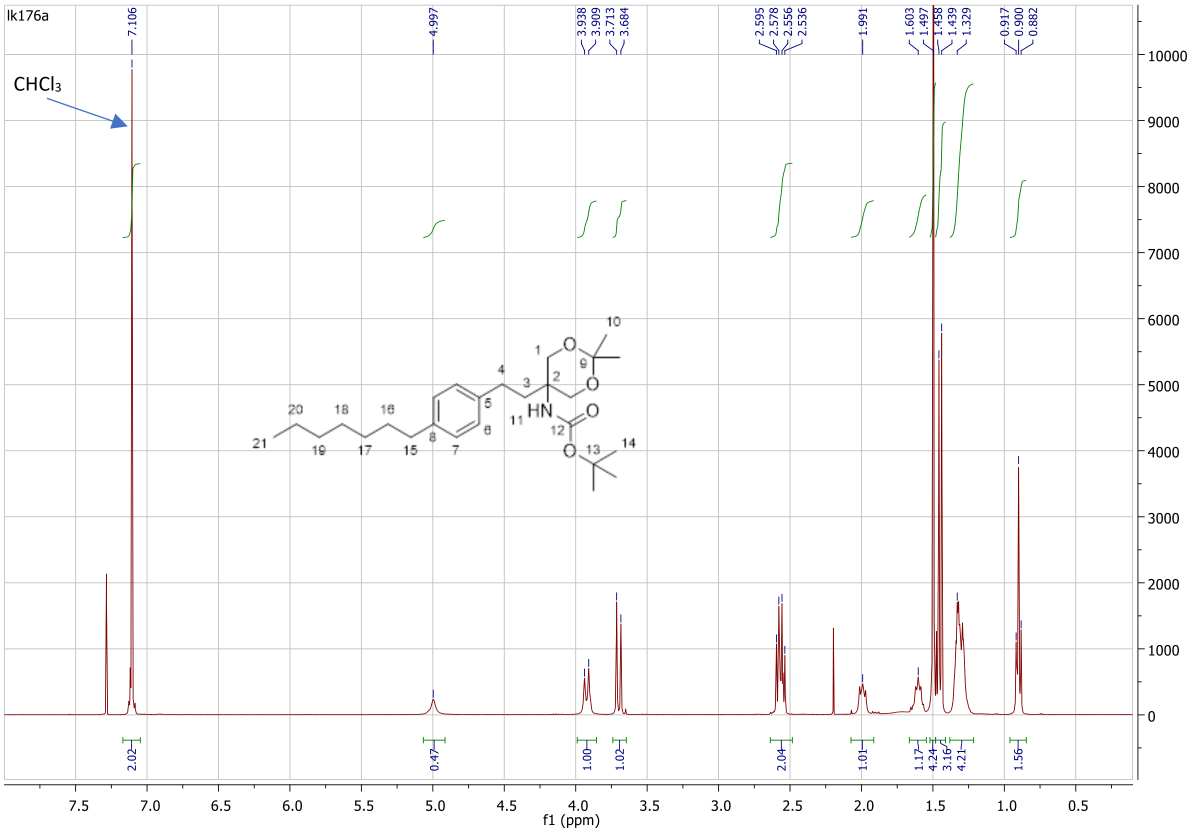

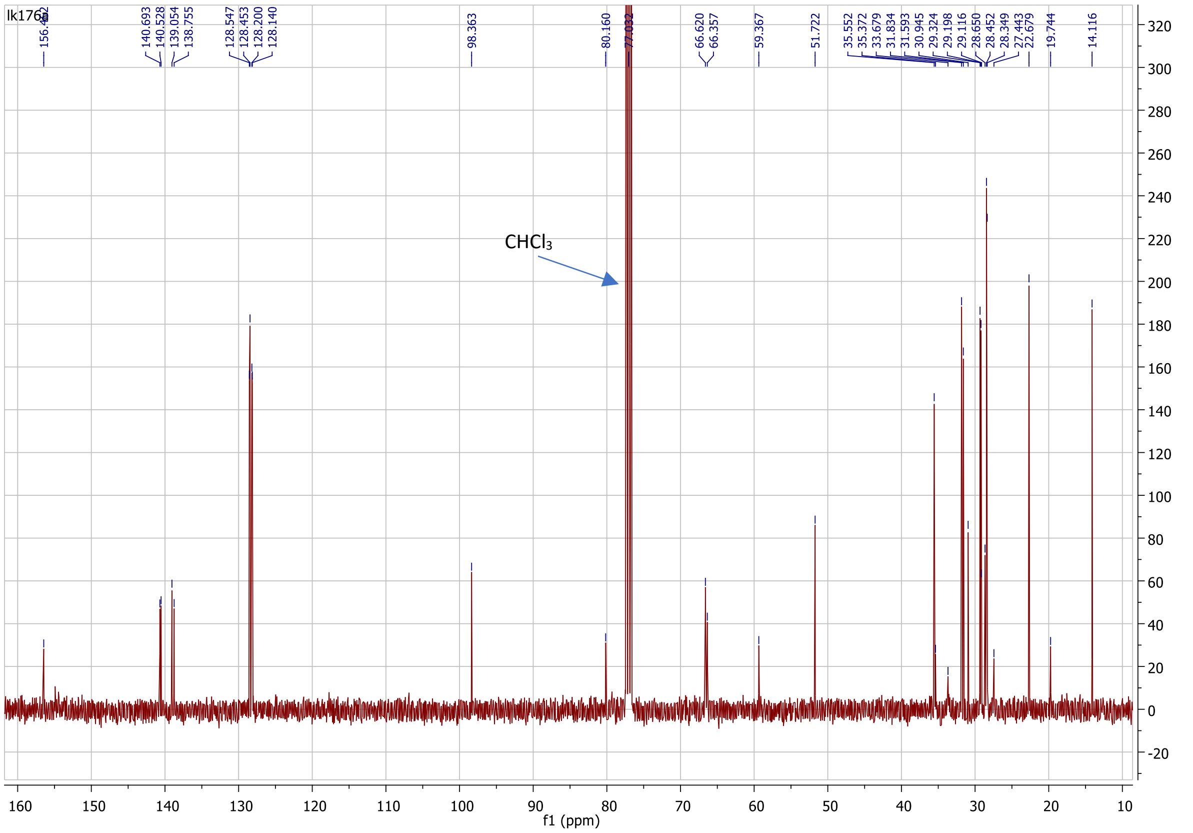

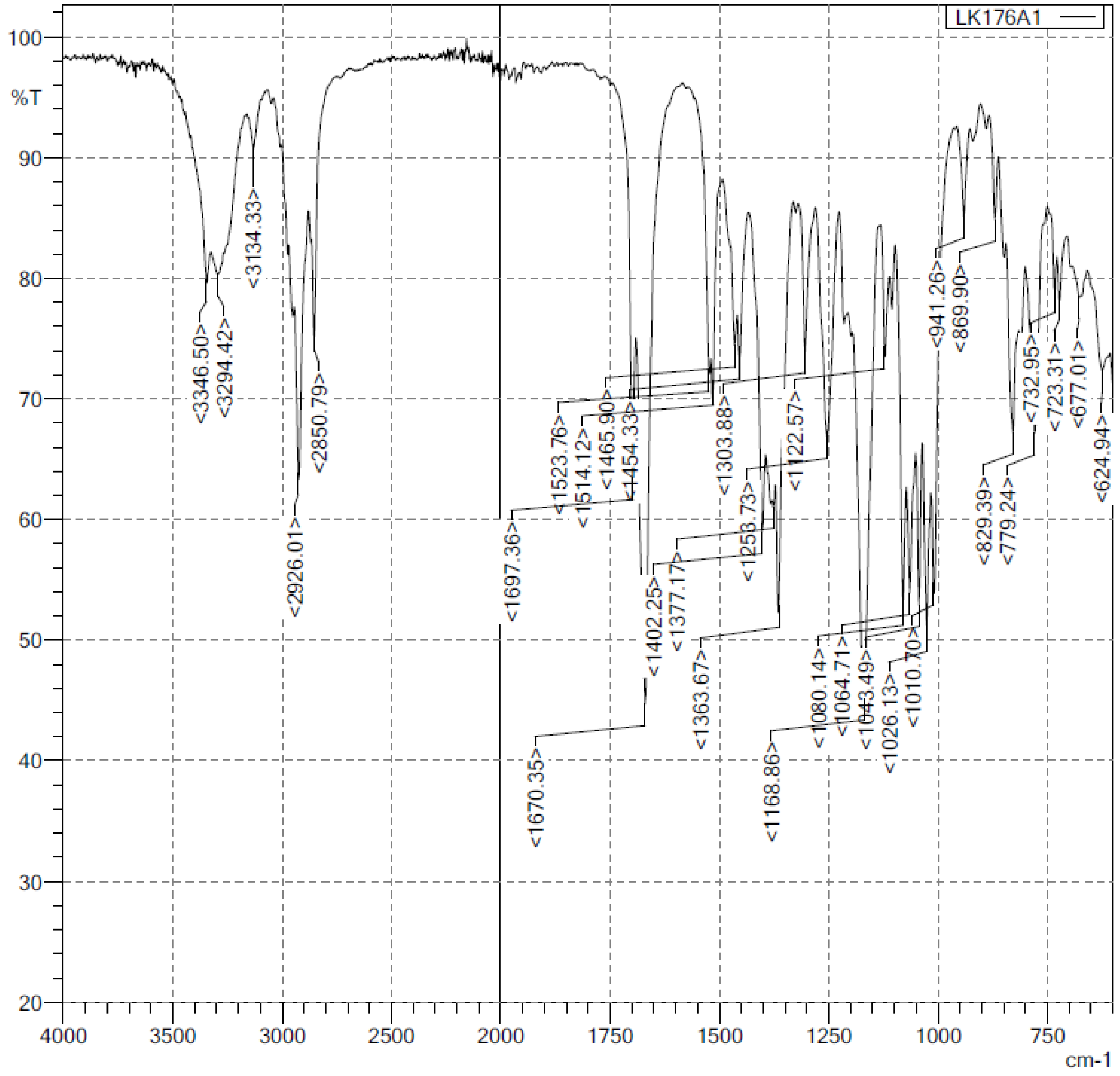
LK176A1
%T
100
90
80
70
60
50
40
30
20
4000
3500
3000
2500
2000
1750
1500
1250
1000
750
cm-1
<3346.50>
<3294.42>
<3134.33>
<2926.01>
<2850.79>
<1697.36>
<1670.35>
<1523.76>
<1514.12>
<1465.90>
<1454.33>
<1402.25>
<1377.17>
<1363.67>
<1303.88>
<1253.73>
<1168.86>
<1122.57>
<1080.14>
<1064.71>
<1043.49>
<1026.13>
<1010.70>
<941.26>
<869.90>
<829.39>
<779.24>
<732.95>
<723.31>
<677.01>
<624.94>

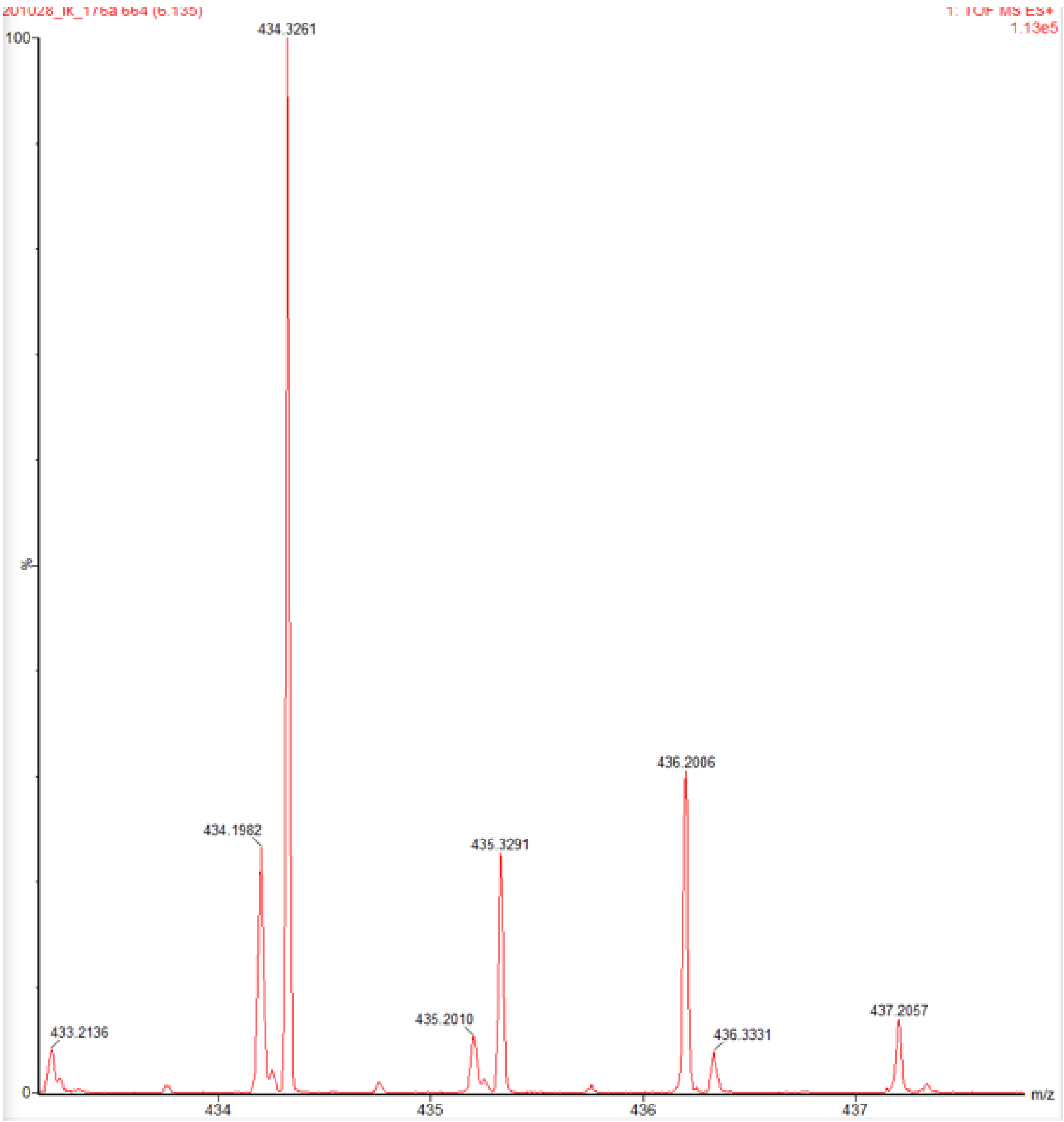
201028_IK_176a 664 (6.135)
1: TOF MS ES+
1.13e5
100
%
0
434.3261
434.1982
435.3291
436.2006
433.2136
435.2010
436.3331
437.2057
434
435
436
437
m/z

tert-butyl (2,2-dimethyl-5-(4-nonylphenethyl)-1,3-dioxan-5-yl)carbamate **8c**

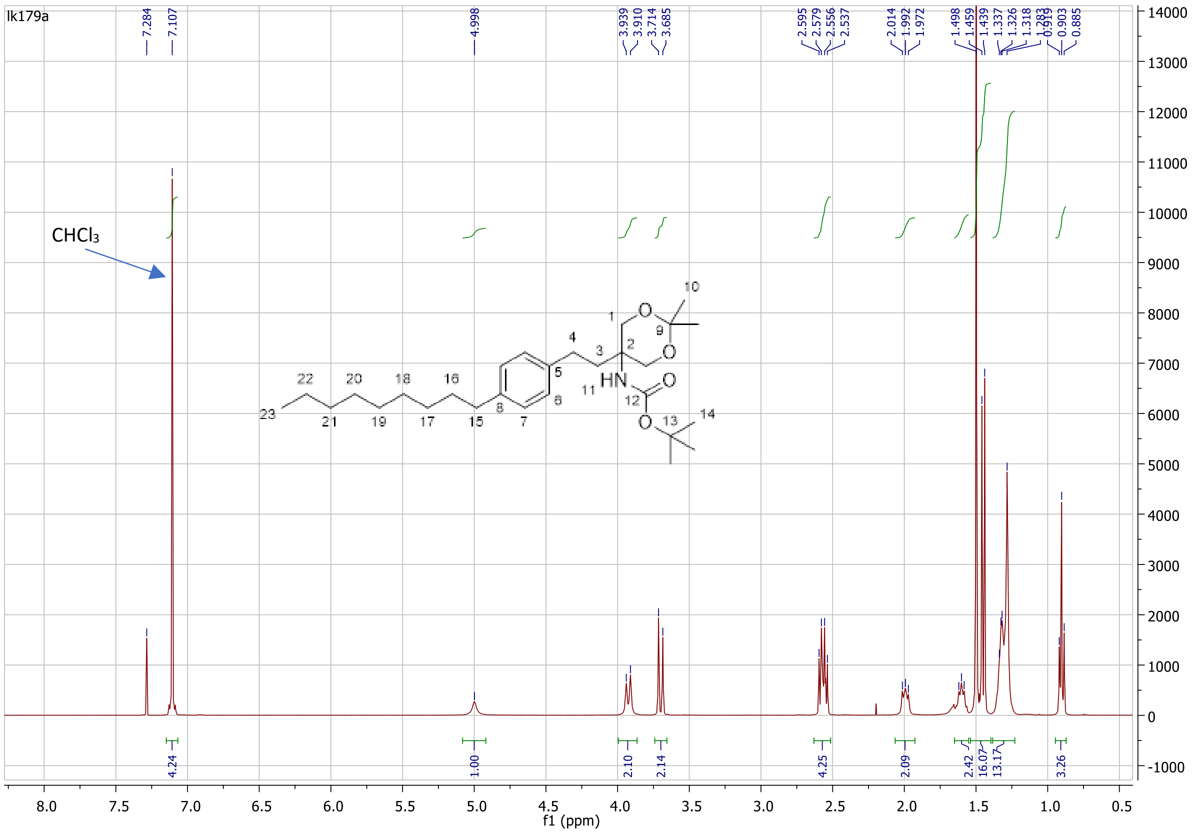

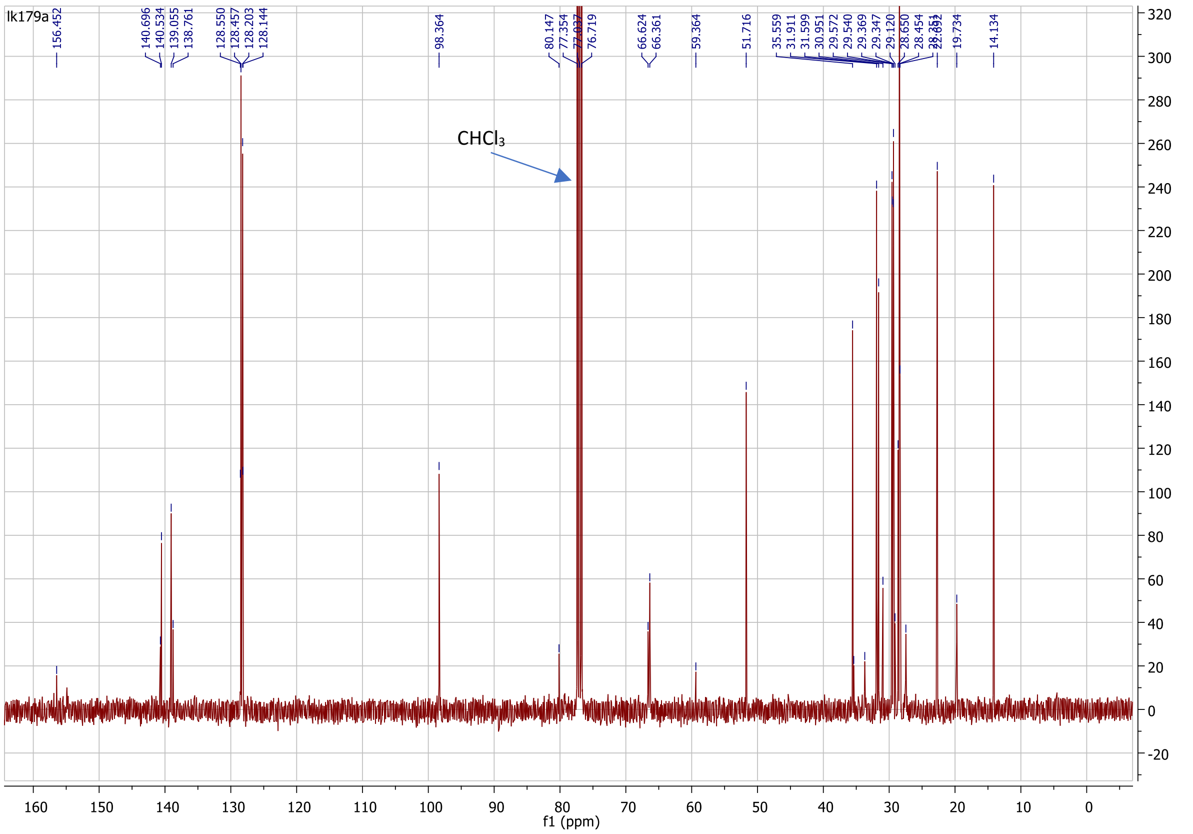

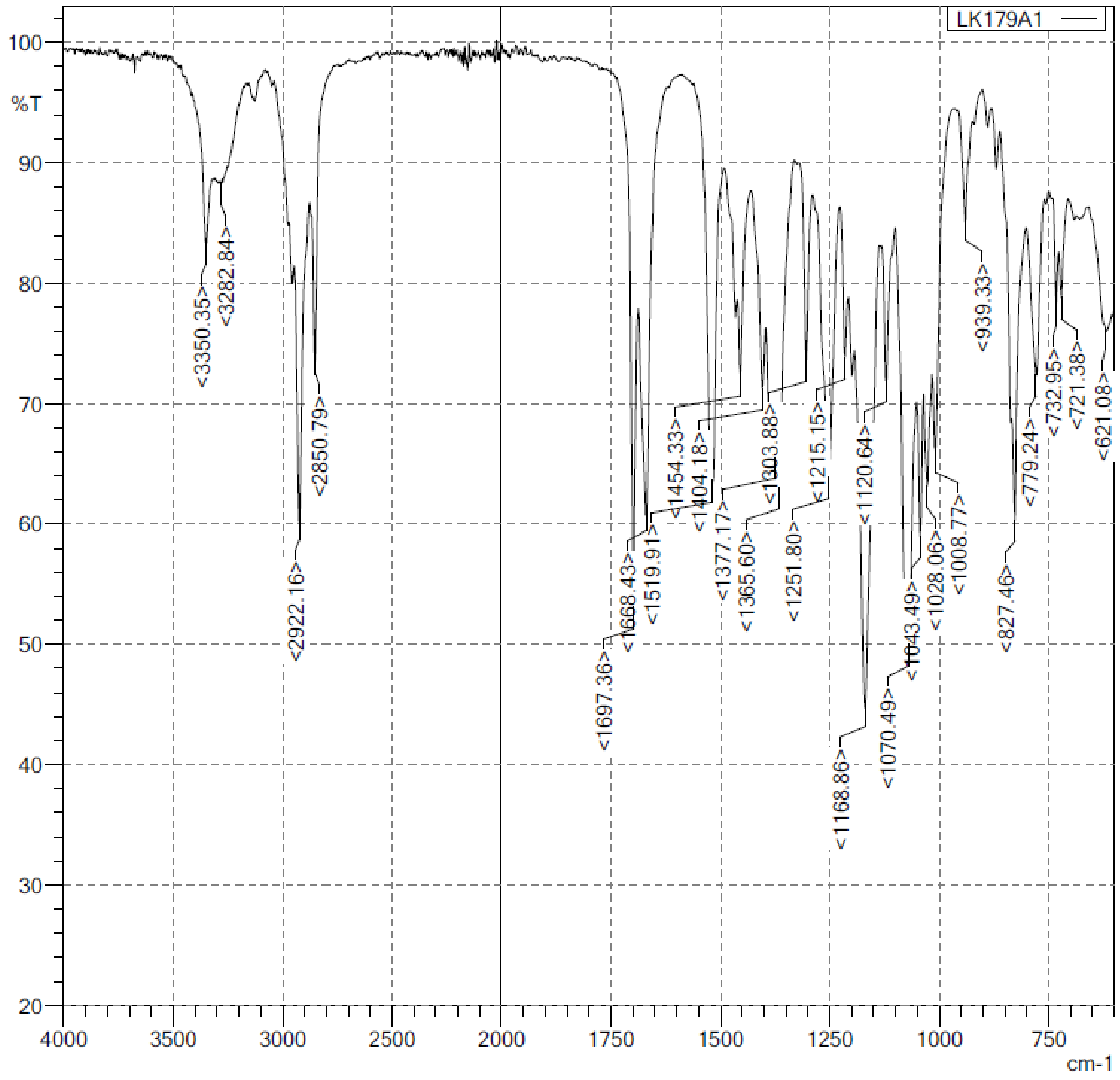
LK179A1
%T
100
90
80
70
60
50
40
30
20
4000
3500
3000
2500
2000
1750
1500
1250
1000
750
cm-1
<3350.35>
<3282.84>
<2922.16>
<2850.79>
<1697.36>
<1668.43>
<1519.91>
<1454.33>
<1404.18>
<1377.17>
<1365.60>
<1303.88>
<1251.80>
<1215.15>
<1168.86>
<1120.64>
<1070.49>
<1043.49>
<1028.06>
<1008.77>
<939.33>
<827.46>
<779.24>
<732.95>
<721.38>
<621.08>

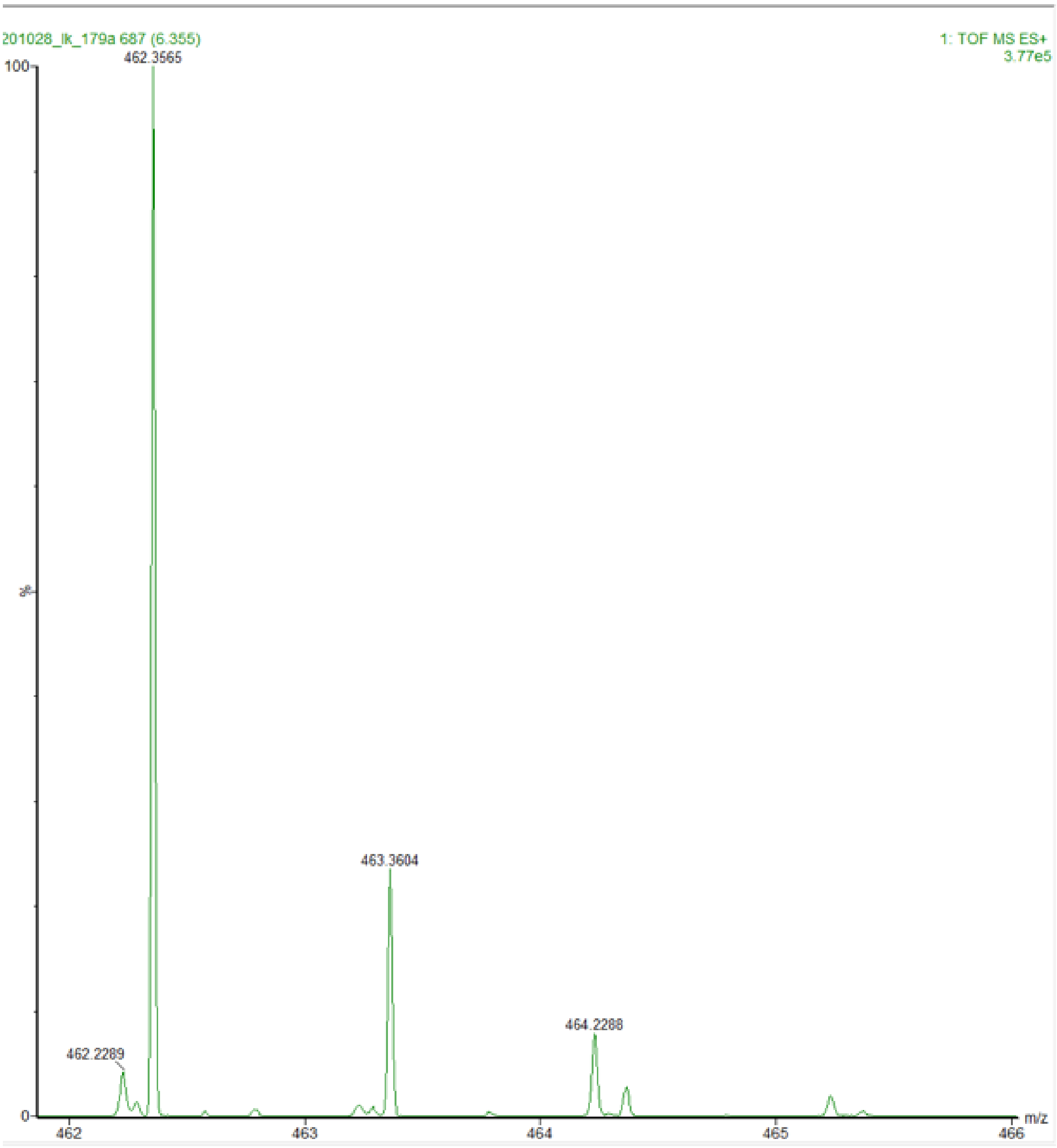
201028_lk_179a 687 (6.355)
1: TOF MS ES+
3.77e5
462.3565
463.3604
464.2288
462.2289
100
%
0
462
463
464
465
466
m/z

tert-butyl (5-(4-decylphenethyl)-2,2-dimethyl-1,3-dioxan-5-yl)carbamate **8d**

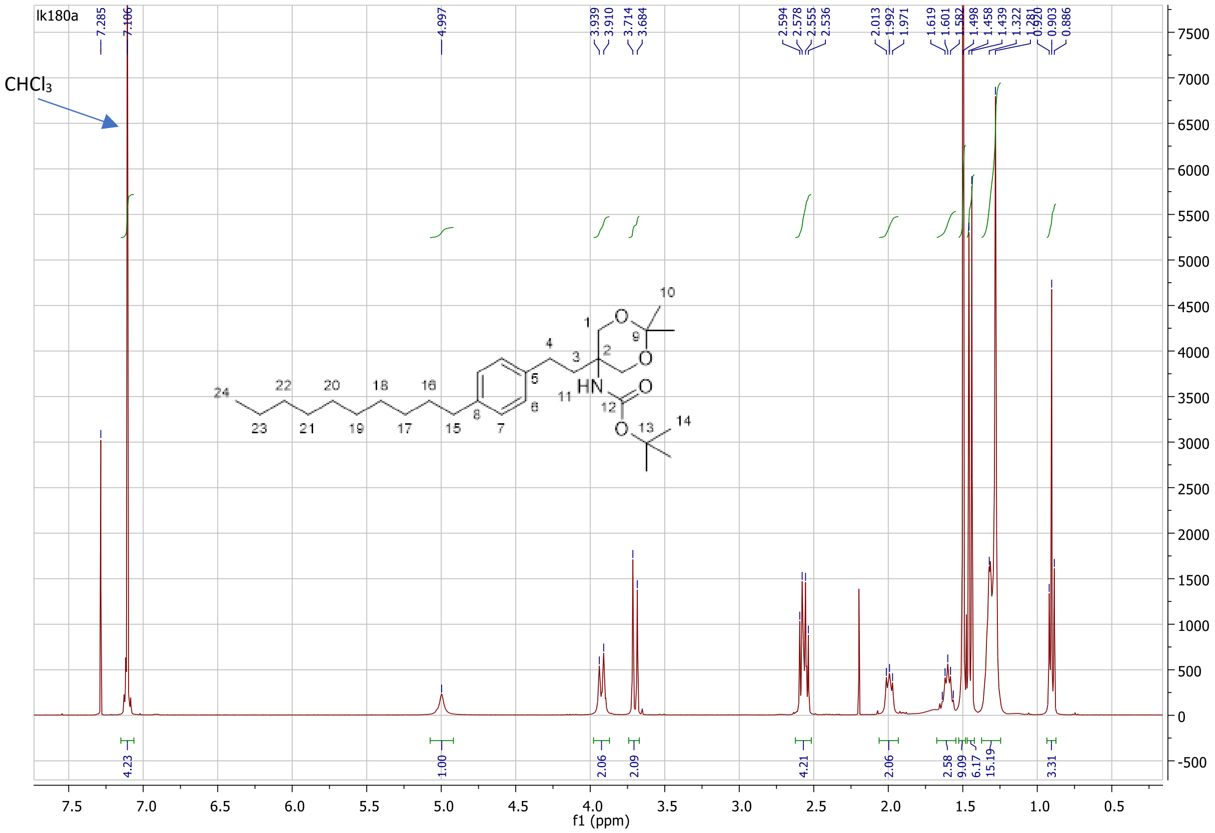

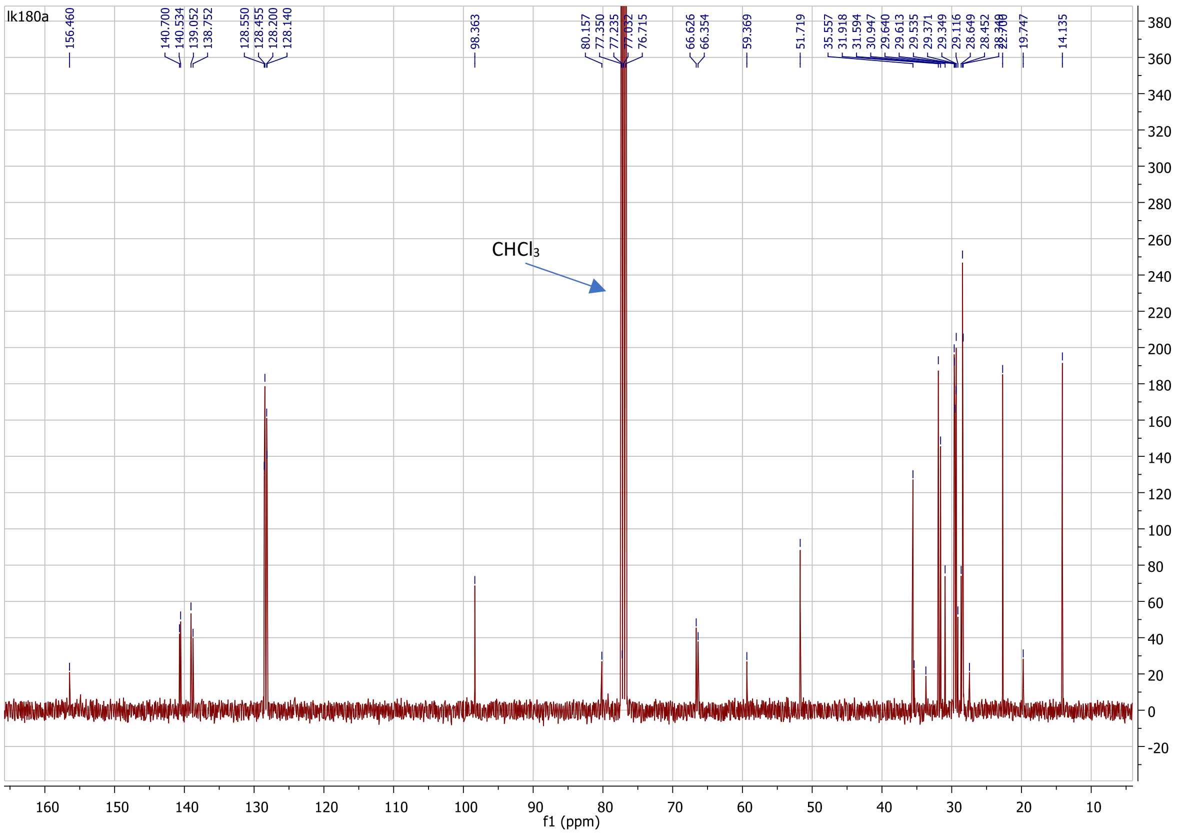

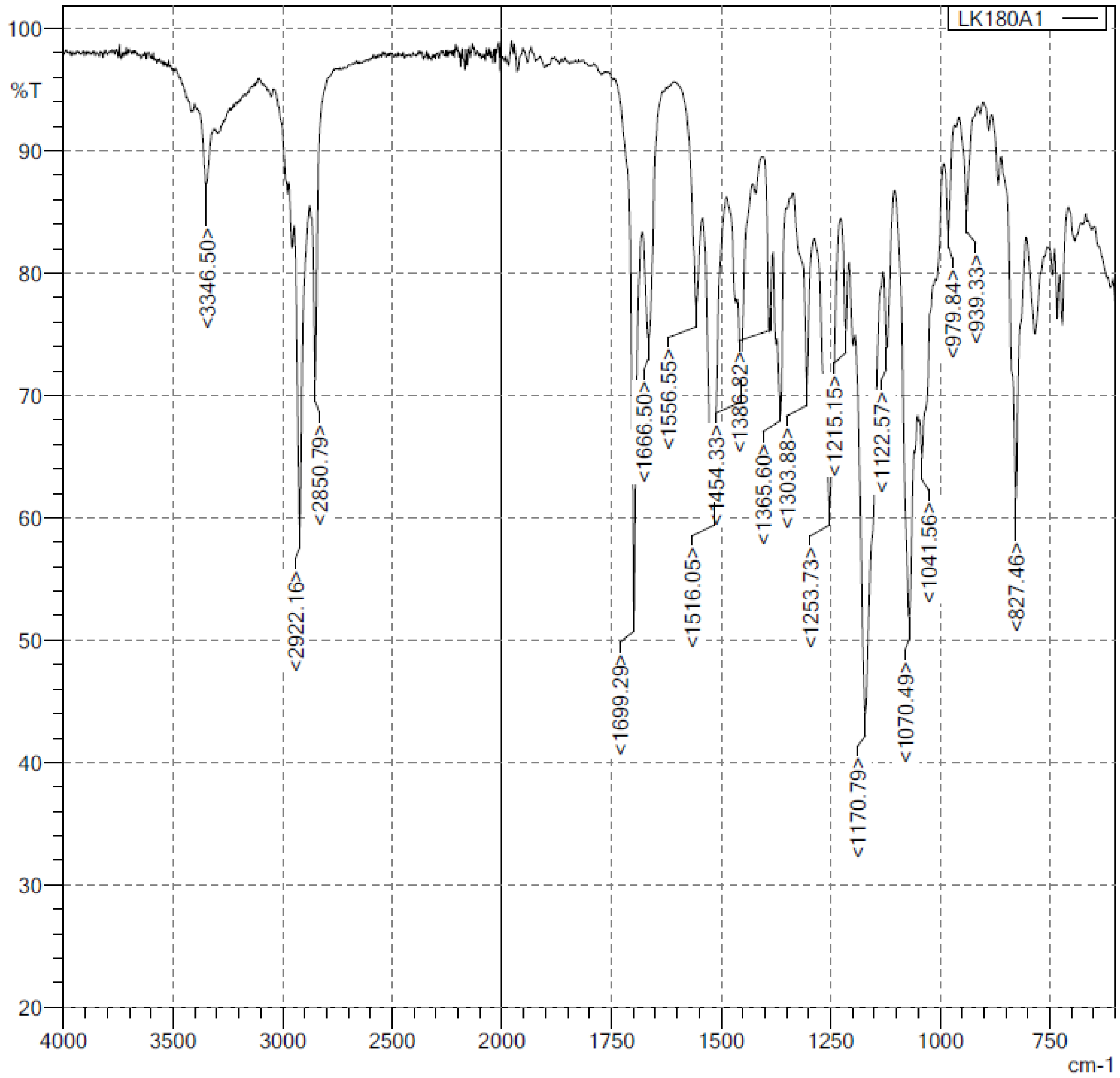
LK180A1
100
%T
90
80
70
60
50
40
30
20
4000
3500
3000
2500
2000
1750
1500
1250
1000
750
cm-1
<3346.50>
<2922.16>
<2850.79>
<1699.29>
<1666.50>
<1556.55>
<1516.05>
<1454.33>
<1386.82>
<1365.60>
<1303.88>
<1253.73>
<1215.15>
<1170.79>
<1122.57>
<1070.49>
<1041.56>
<979.84>
<939.33>
<827.46>

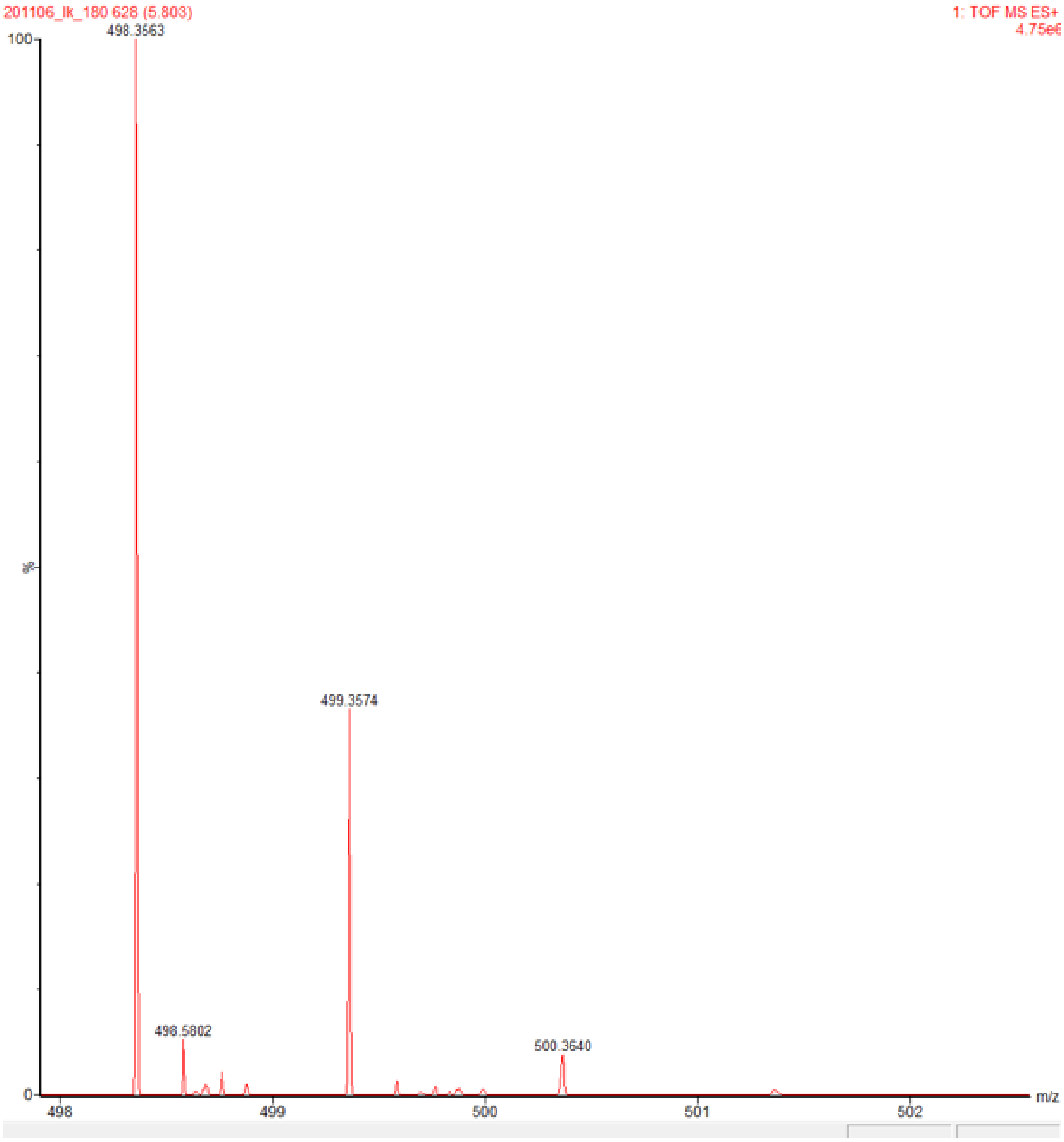

201106_lk_180 628 (5.803)
1: TOF MS ES+
4.75e6
100
%
0
498.3563
499.3574
498.5802
500.3640
498
499
500
501
502
m/z

2-amino-2-(4-hexylphenethyl)propane-1,3-diol **9a**

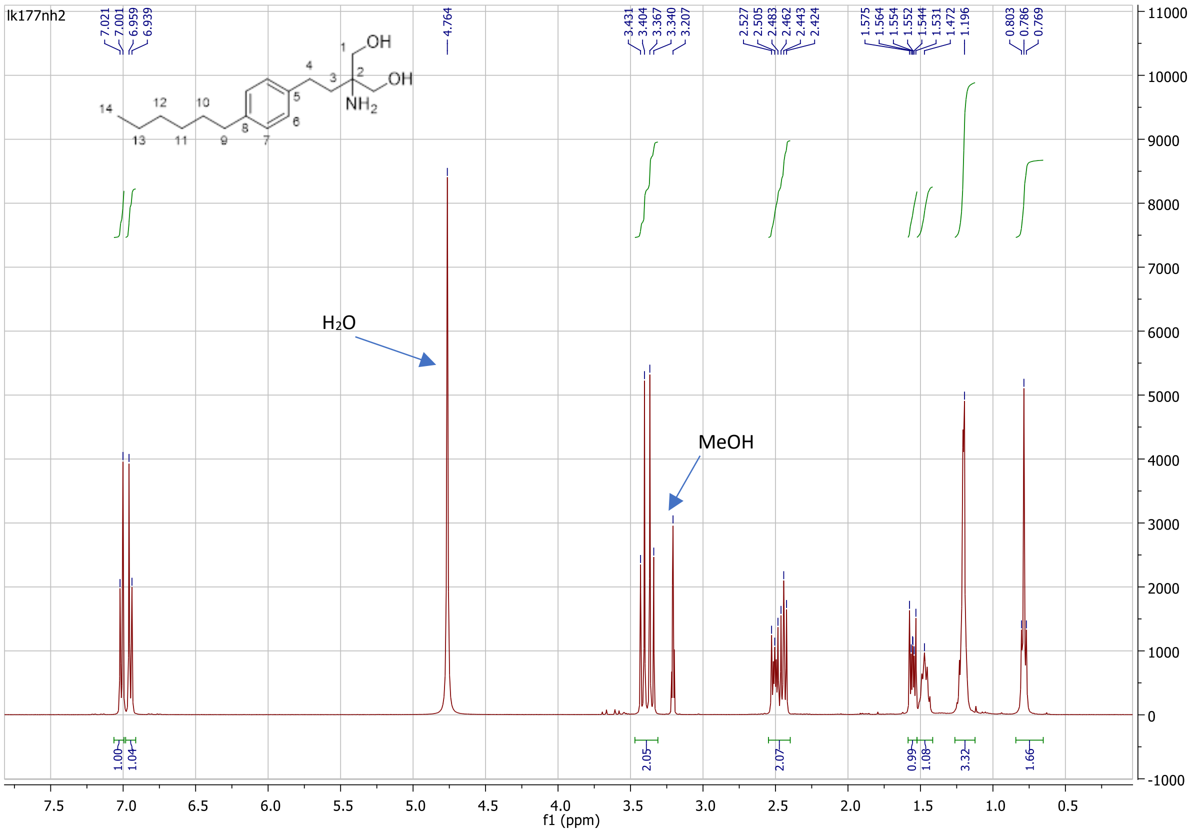

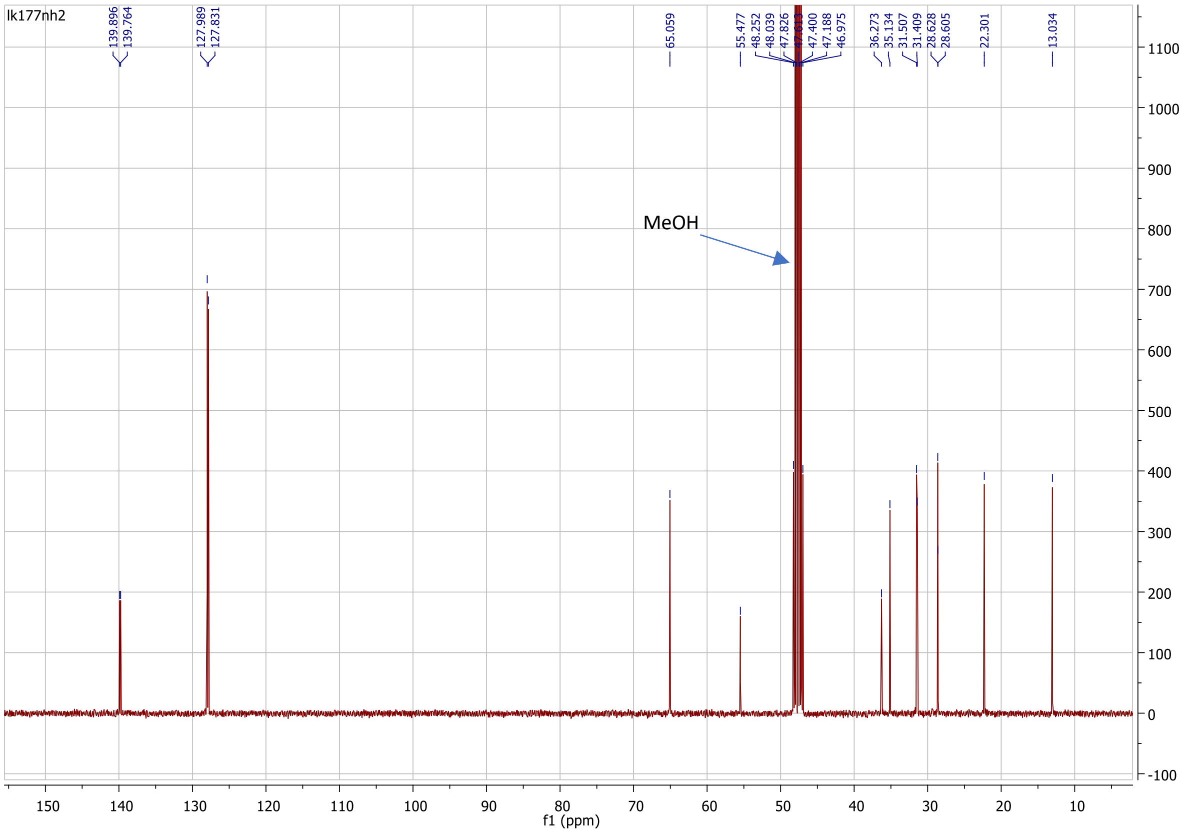

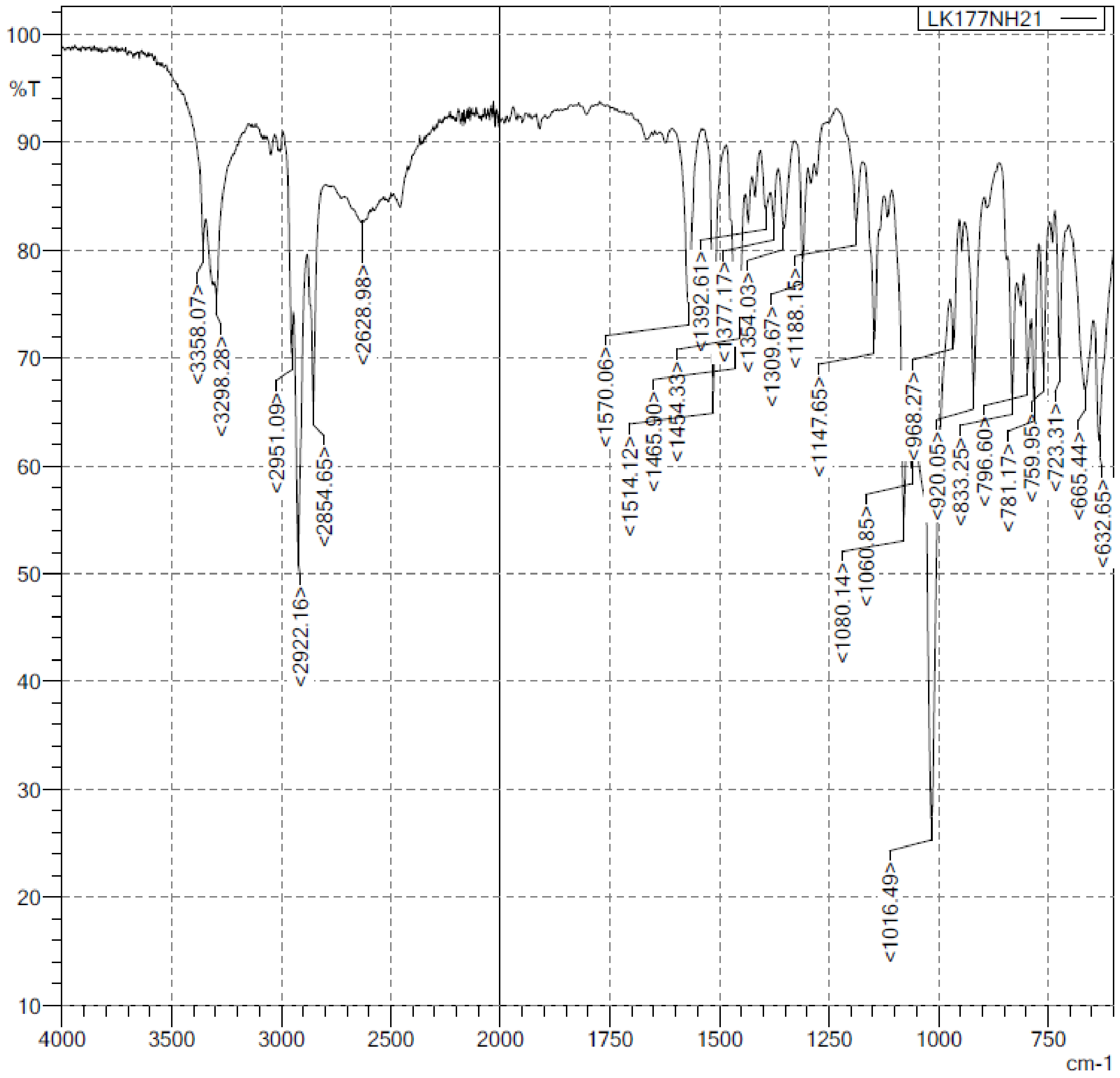
LK177NH21
%T
cm-1
3358.07
3298.28
2951.09
2922.16
2854.65
2628.98
1570.06
1514.12
1465.90
1454.33
1392.61
1377.17
1354.03
1309.67
1188.15
1147.65
1080.14
1060.85
1016.49
968.27
920.05
833.25
796.60
781.17
759.95
723.31
665.44
632.65

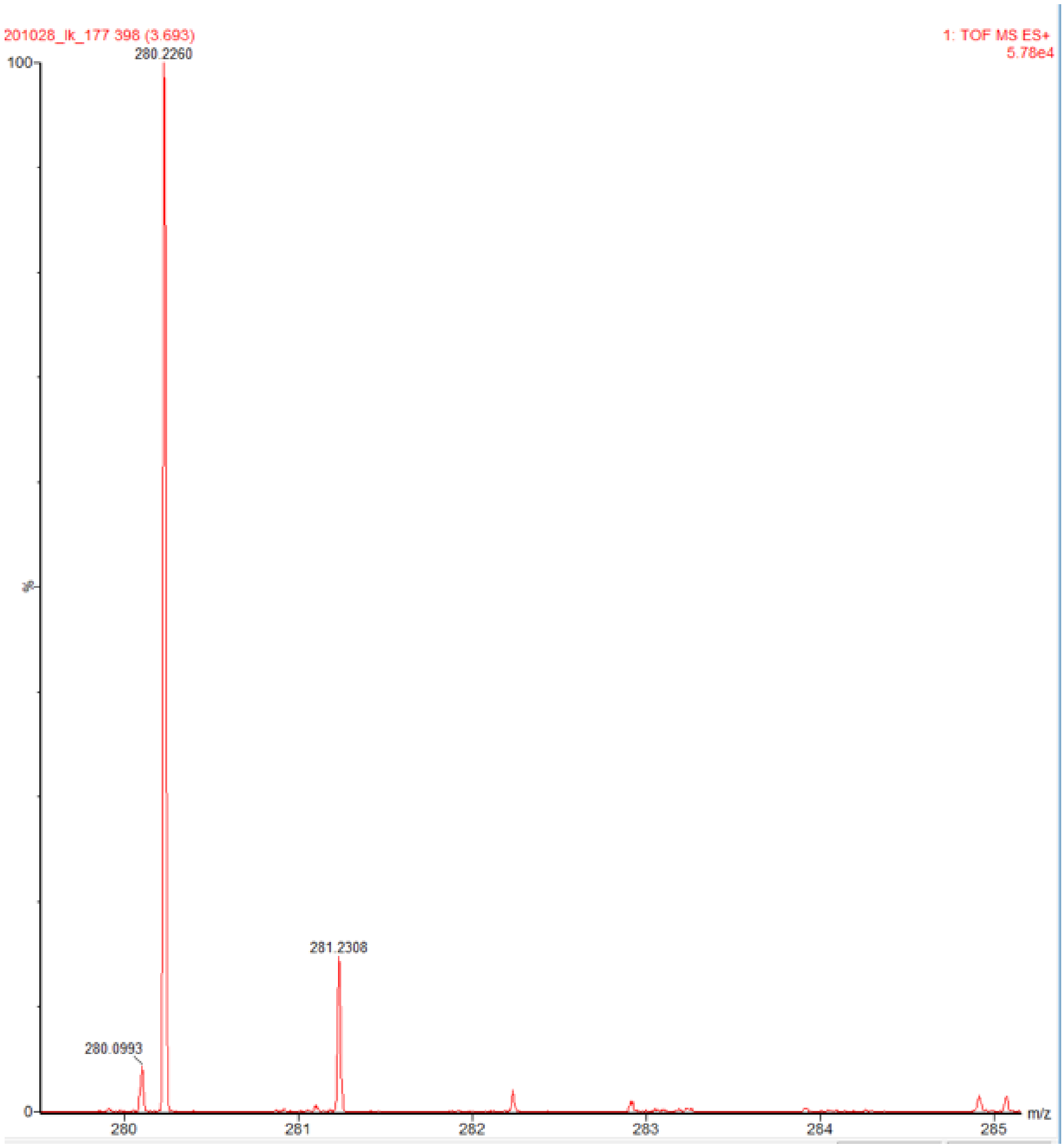

201028_lk_177 398 (3.693)
1: TOF MS ES+
5.78e4
100
280.2260
%
281.2308
280.0993
0
280
281
282
283
284
285
m/z

2-amino-2-(4-heptylphenethyl)propane-1,3-diol **9b**

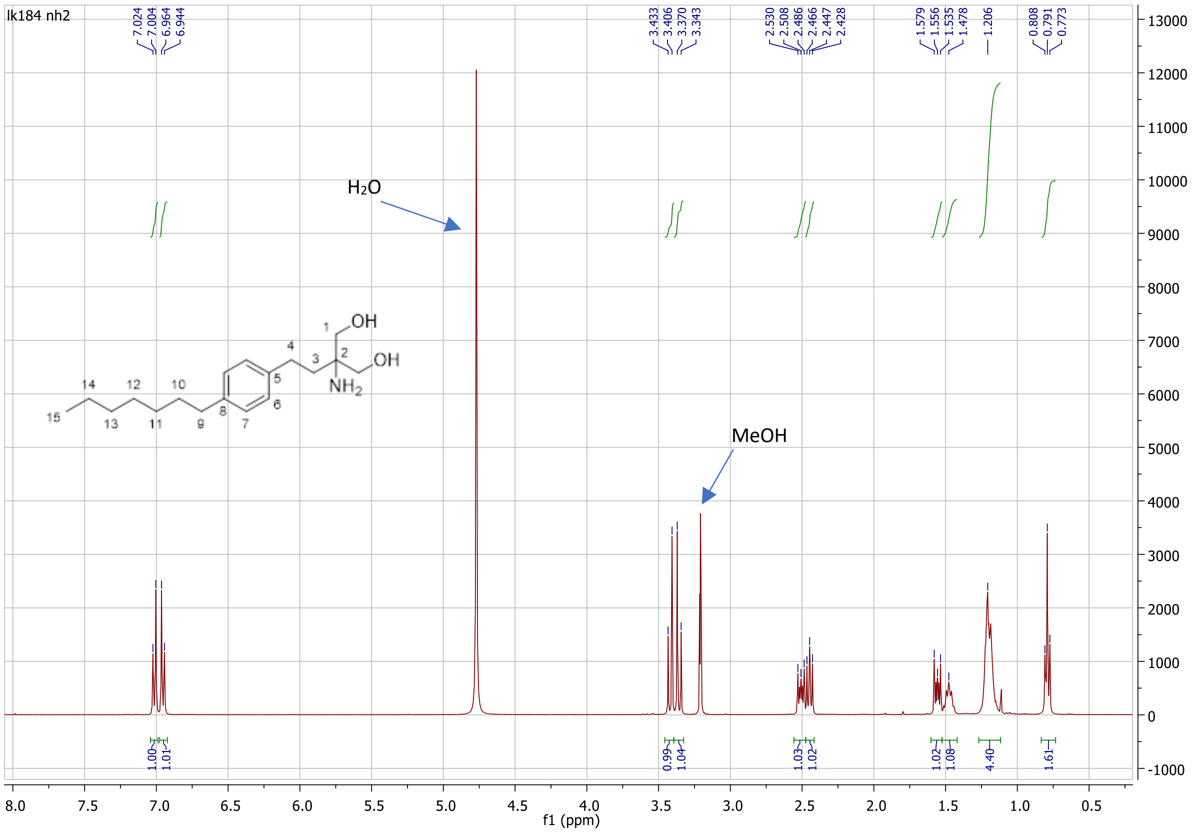

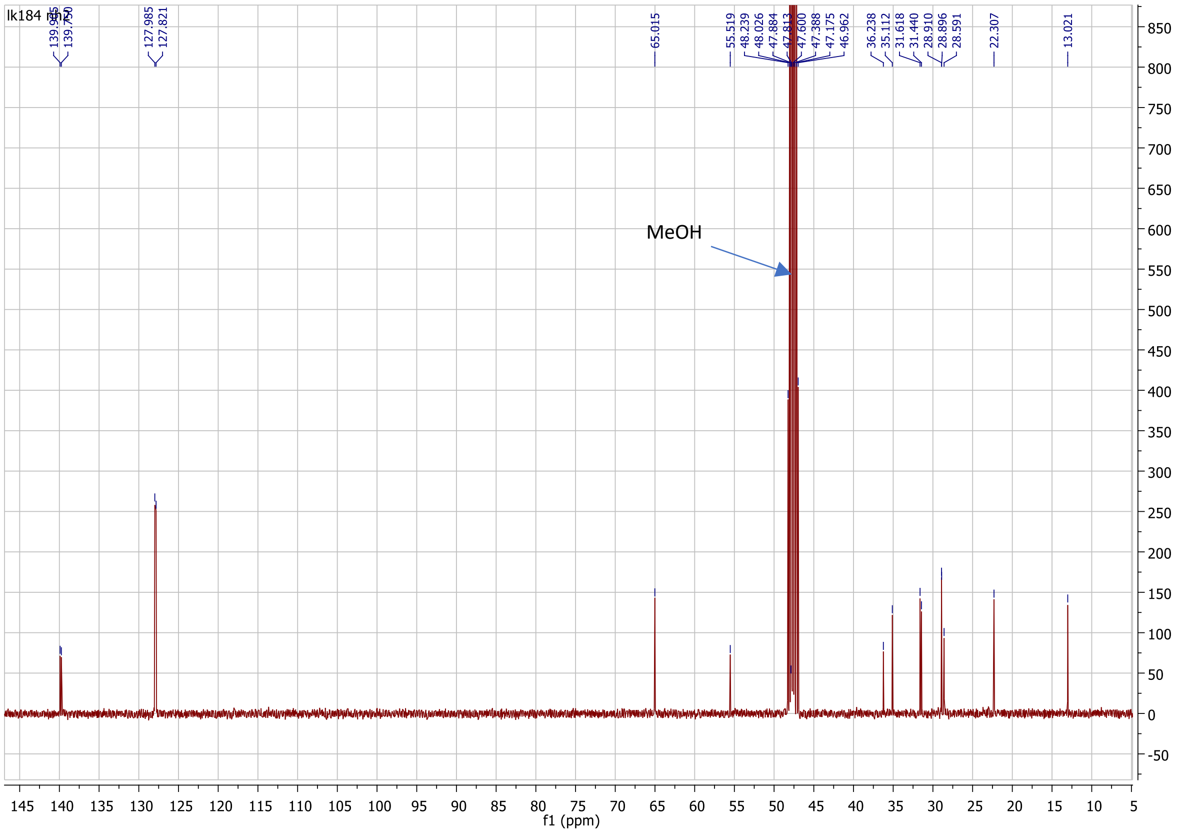

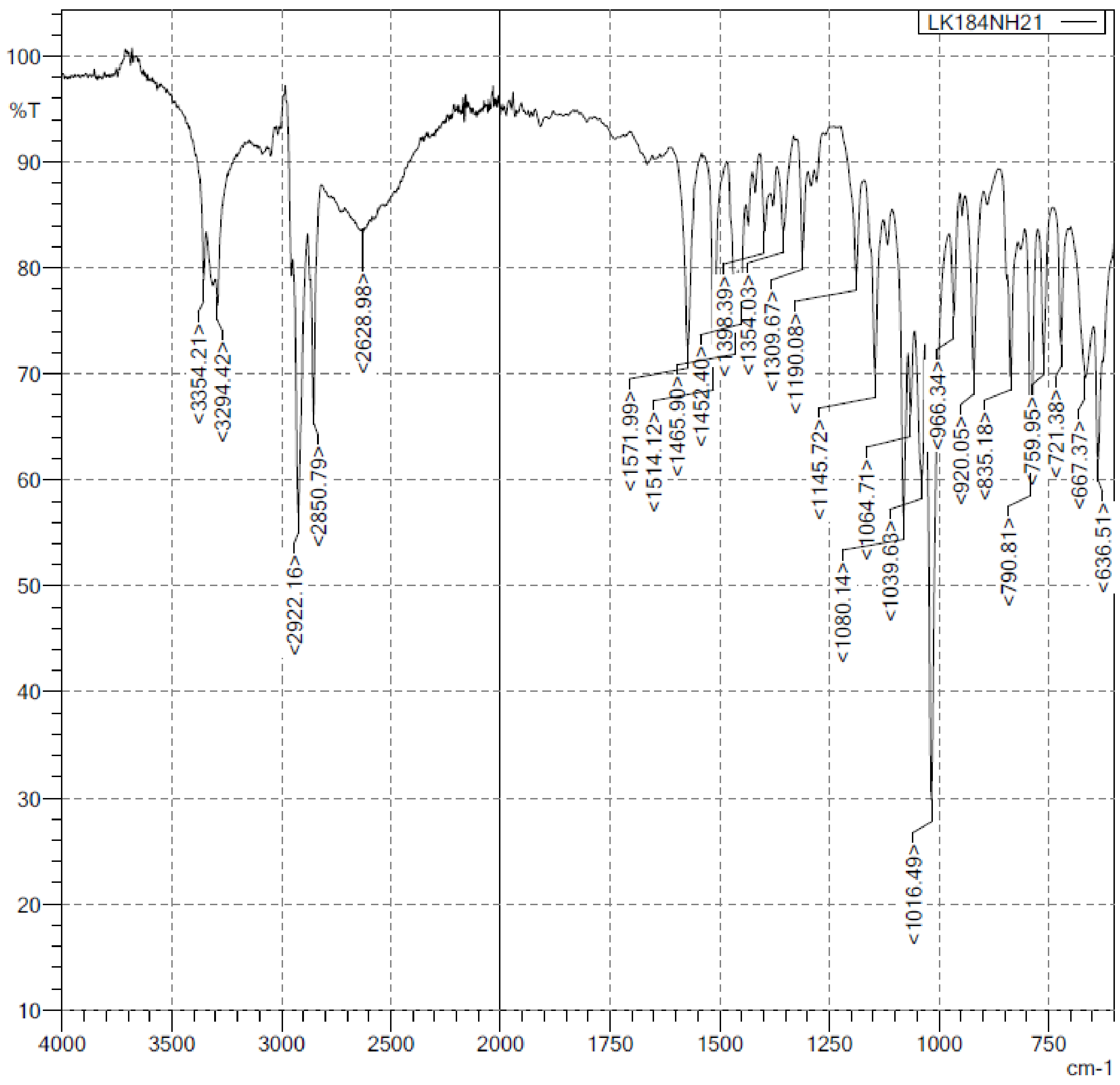
LK184NH21
100
%T
90
80
70
60
50
40
30
20
10
4000
3500
3000
2500
2000
1750
1500
1250
1000
750
cm-1
<3354.21>
<3294.42>
<2922.16>
<2850.79>
<2628.98>
<1571.99>
<1514.12>
<1465.90>
<1452.40>
<1398.39>
<1354.03>
<1309.67>
<1190.08>
<1145.72>
<1080.14>
<1064.71>
<1039.63>
<1016.49>
<966.34>
<920.05>
<835.18>
<790.81>
<759.95>
<721.38>
<667.37>
<636.51>

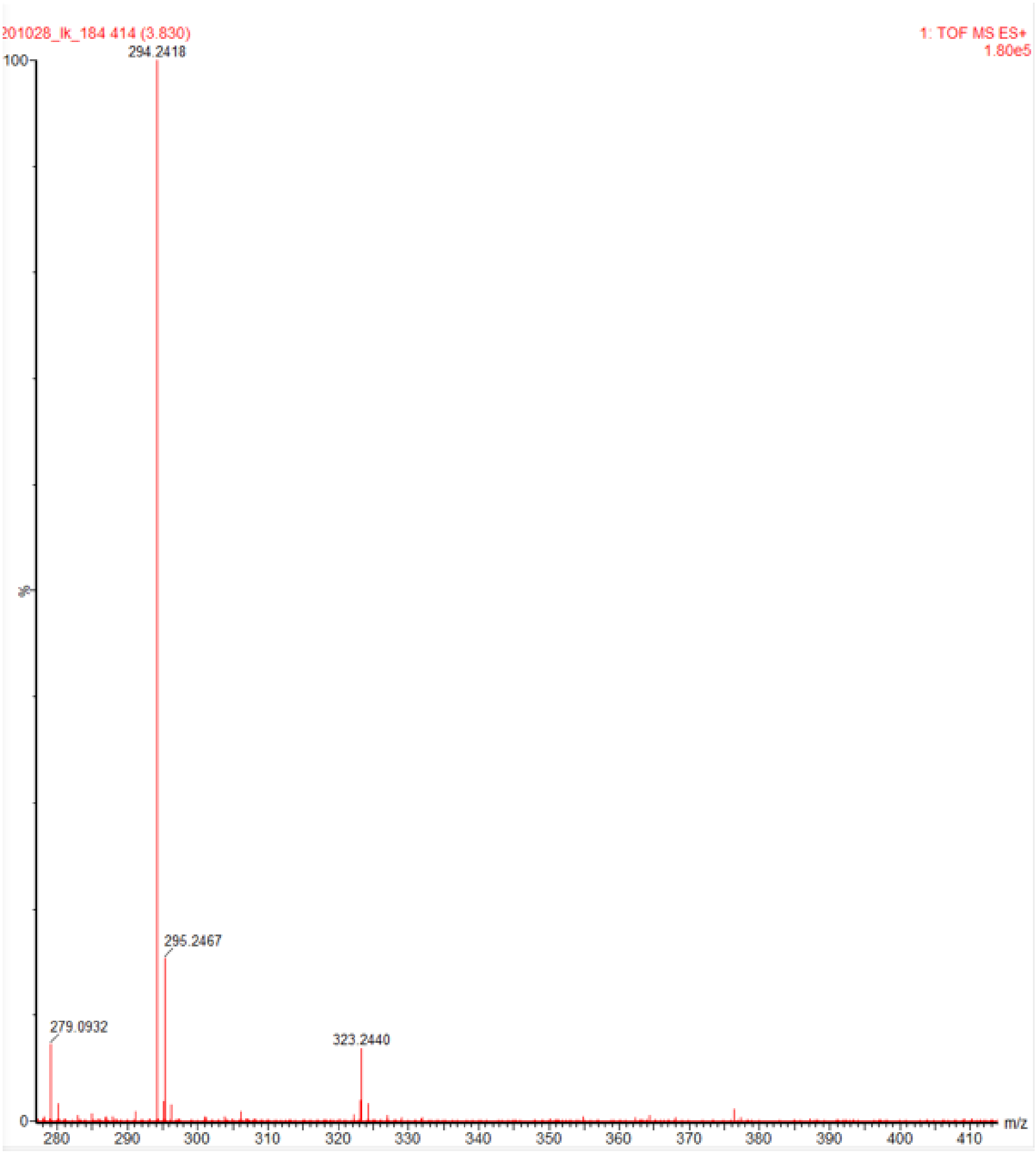

201028_lk_184 414 (3.830)
1: TOF MS ES+
1.80e5
294.2418
295.2467
279.0932
323.2440
100
%
0
280
290
300
310
320
330
340
350
360
370
380
390
400
410
m/z

2-amino-2-(4-octylphenethyl)propane-1,3-diol **9c**

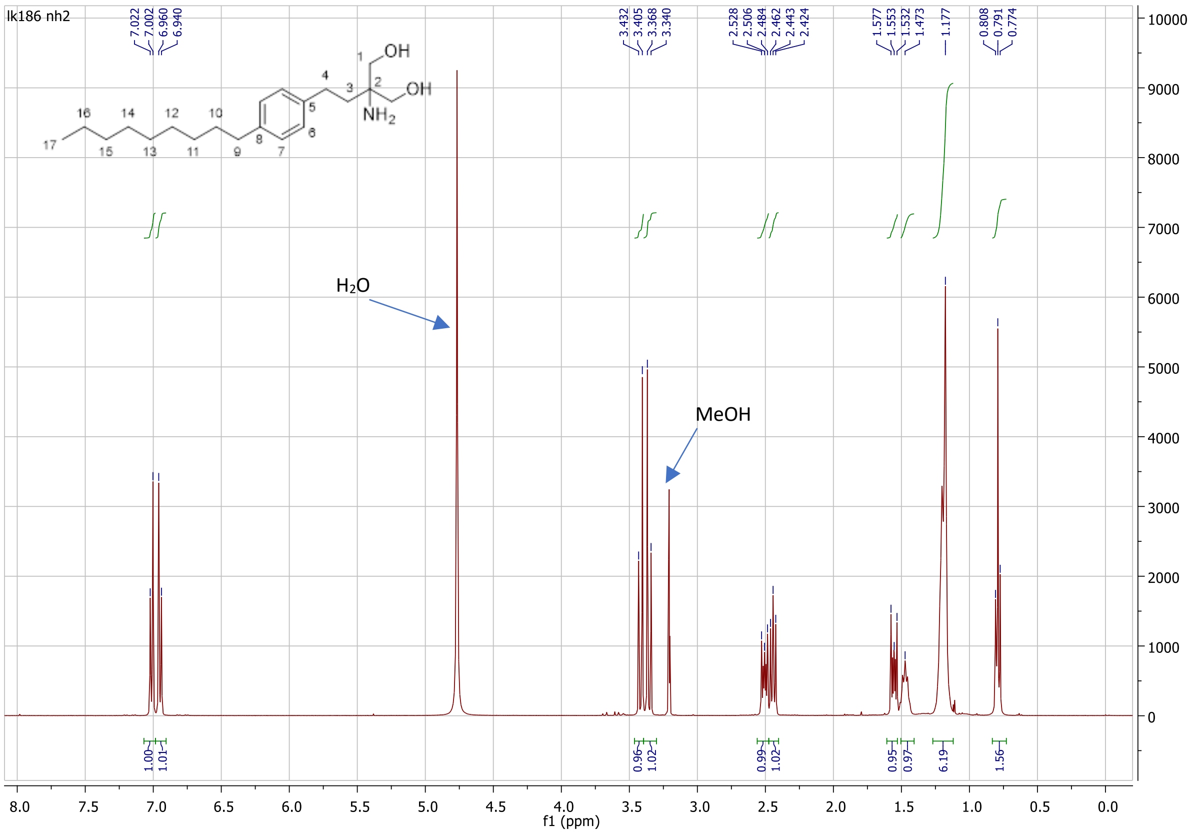

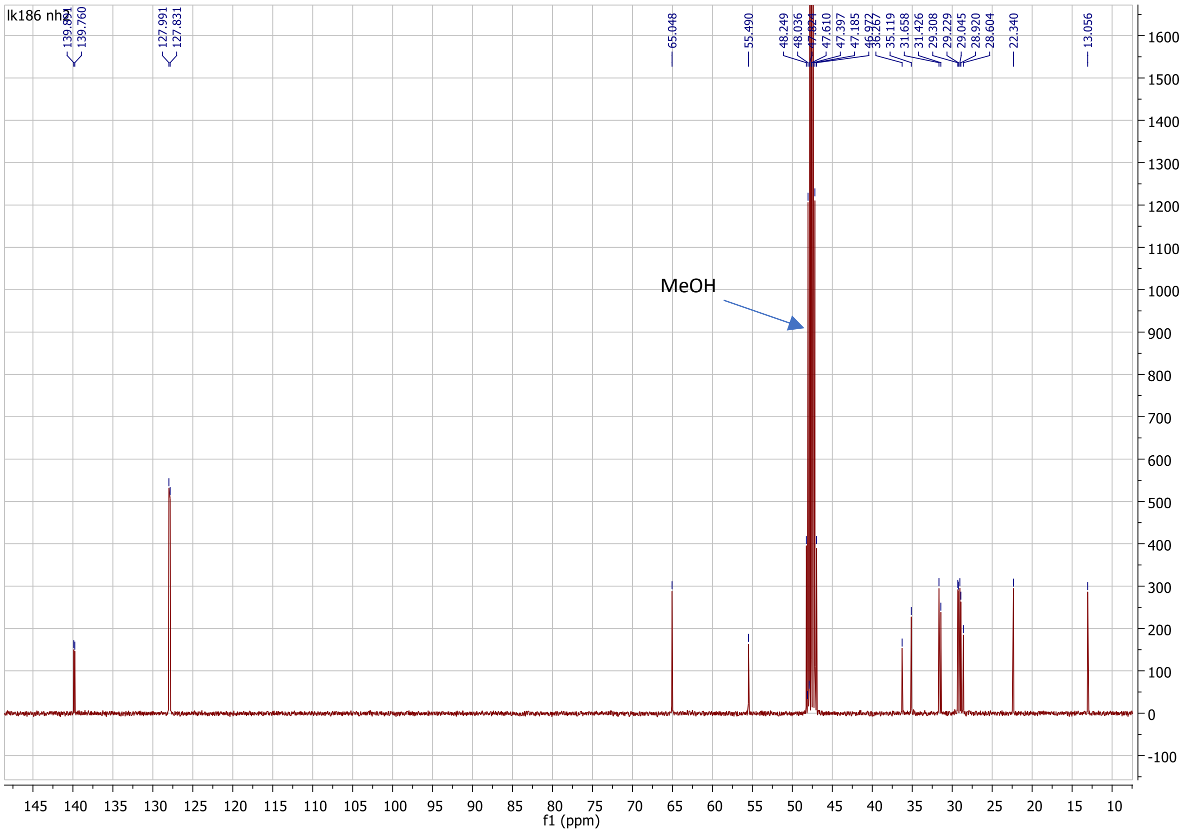

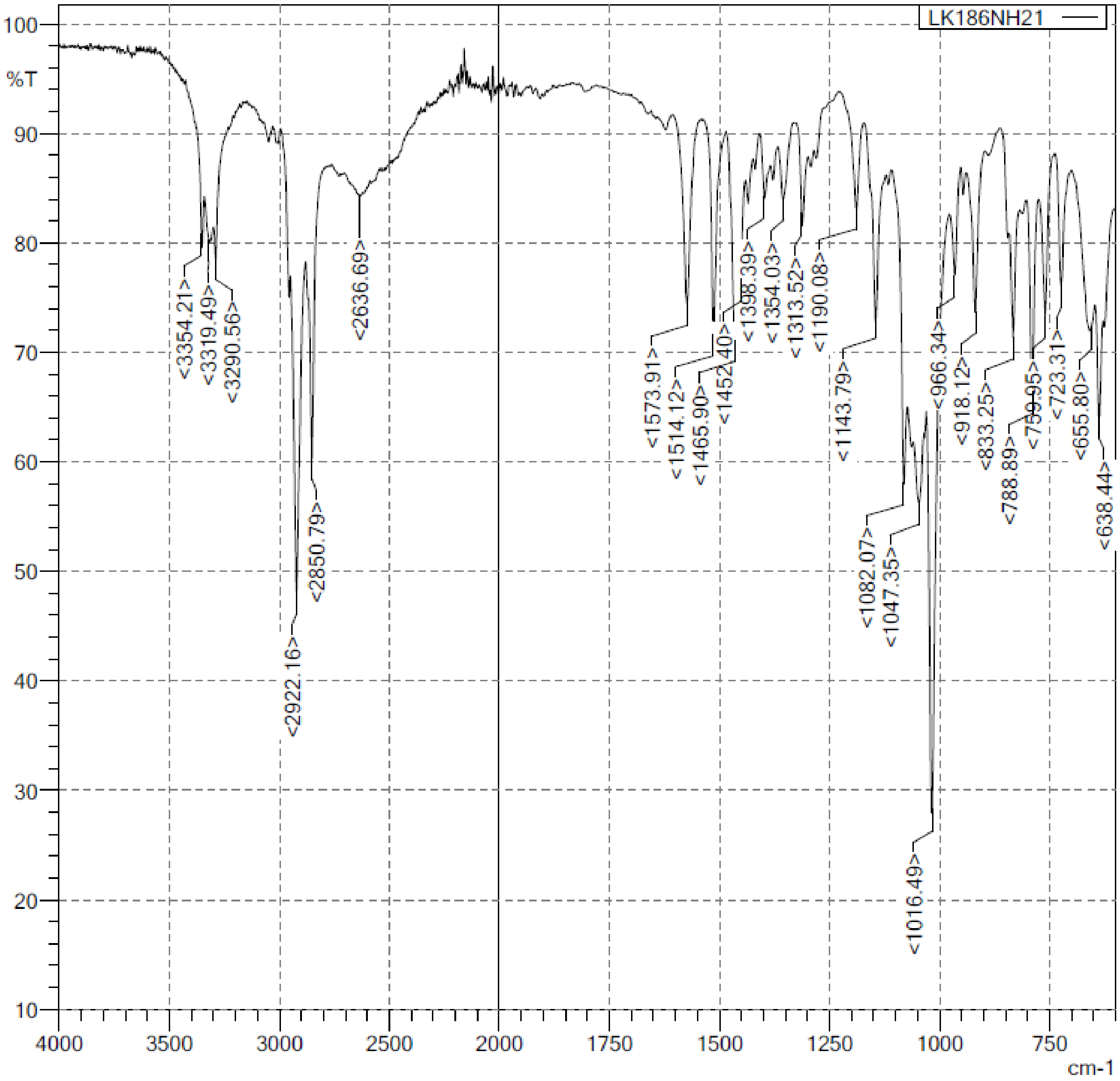
LK186NH21
%T
cm-1
<3354.21>
<3319.49>
<3290.56>
<2922.16>
<2850.79>
<2636.69>
<1573.91>
<1514.12>
<1465.90>
<1452.40>
<1398.39>
<1354.03>
<1313.52>
<1190.08>
<1143.79>
<1082.07>
<1047.35>
<1016.49>
<966.34>
<918.12>
<833.25>
<788.89>
<759.95>
<723.31>
<655.80>
<638.44>

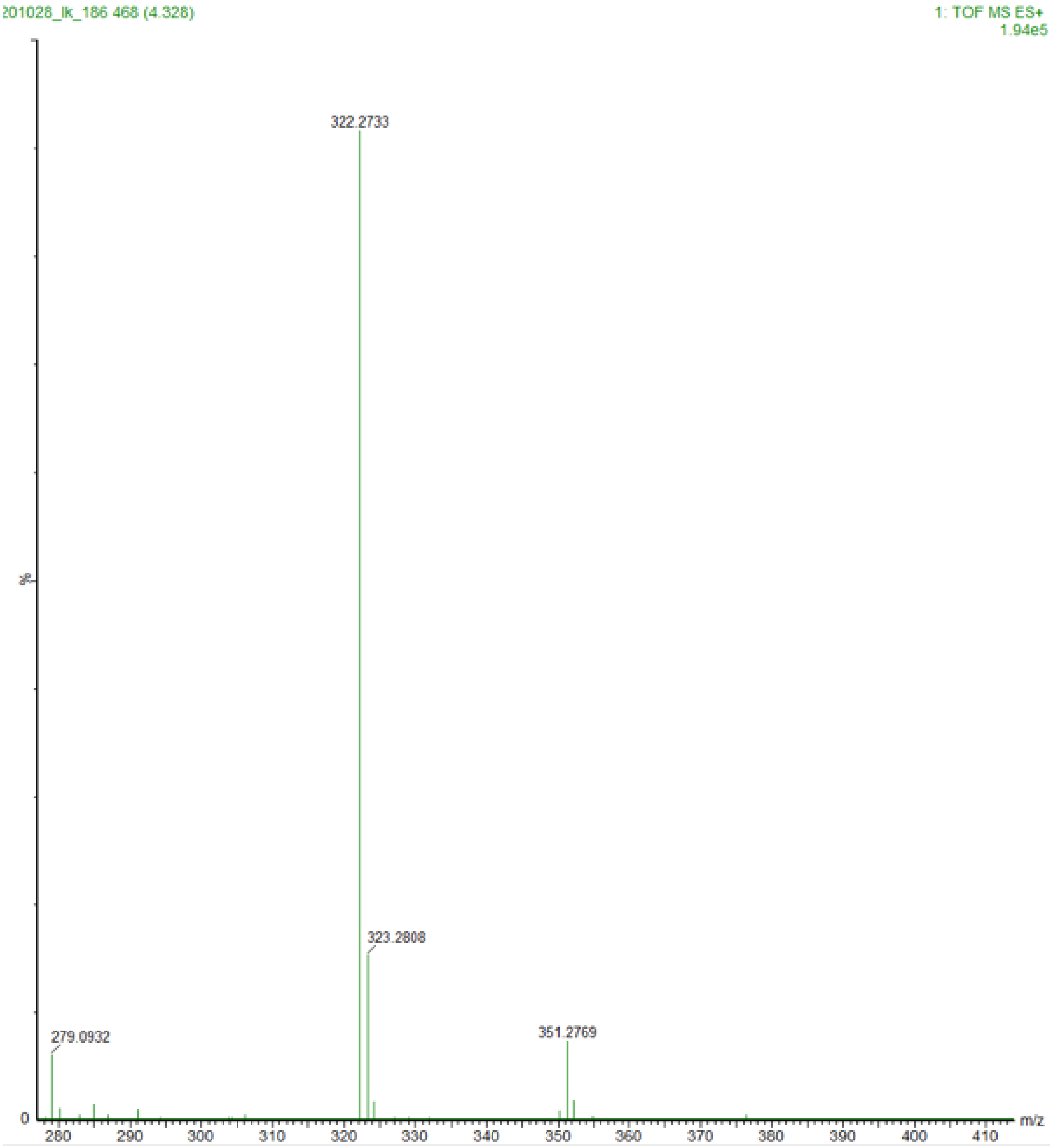
201028_lk_186 468 (4.328)
1: TOF MS ES+
1.94e5
322.2733
323.2808
279.0932
351.2769
%
0
280
290
300
310
320
330
340
350
360
370
380
390
400
410
m/z

2-amino-2-(4-decylphenethyl)propane-1,3-diol **9d**

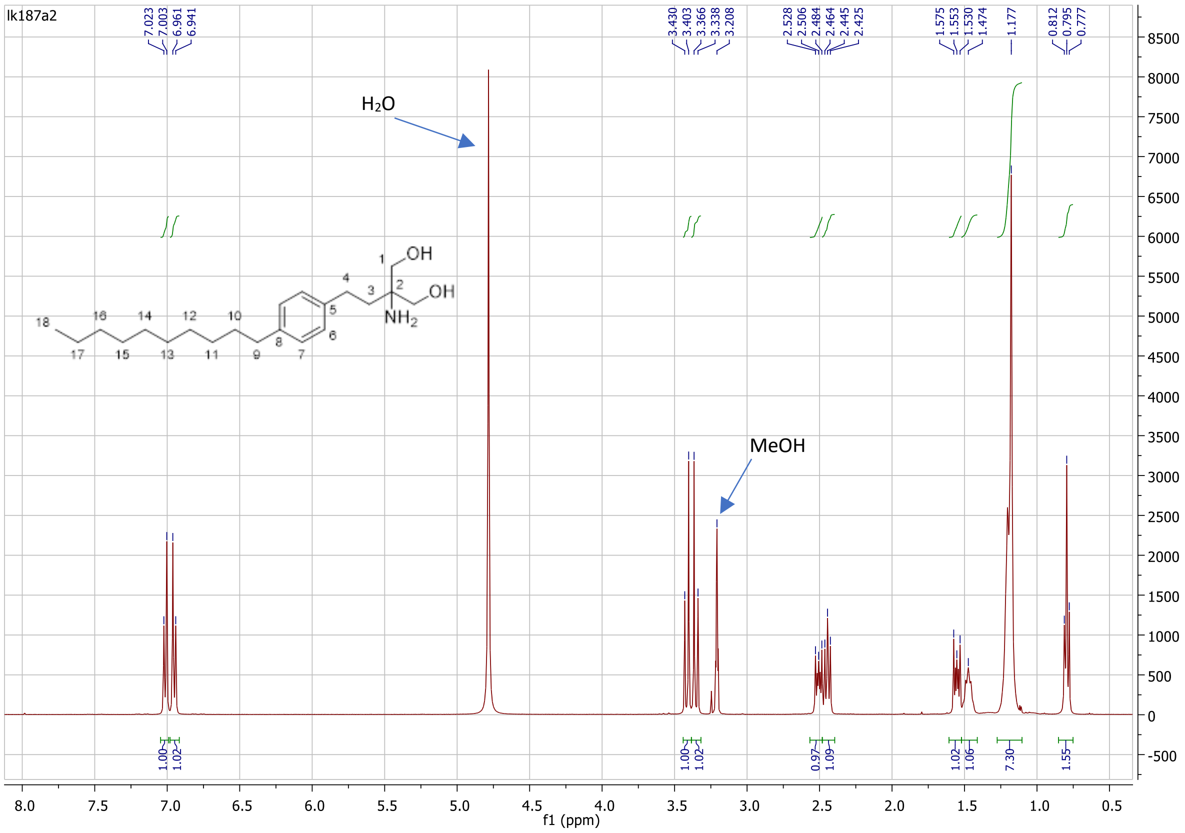

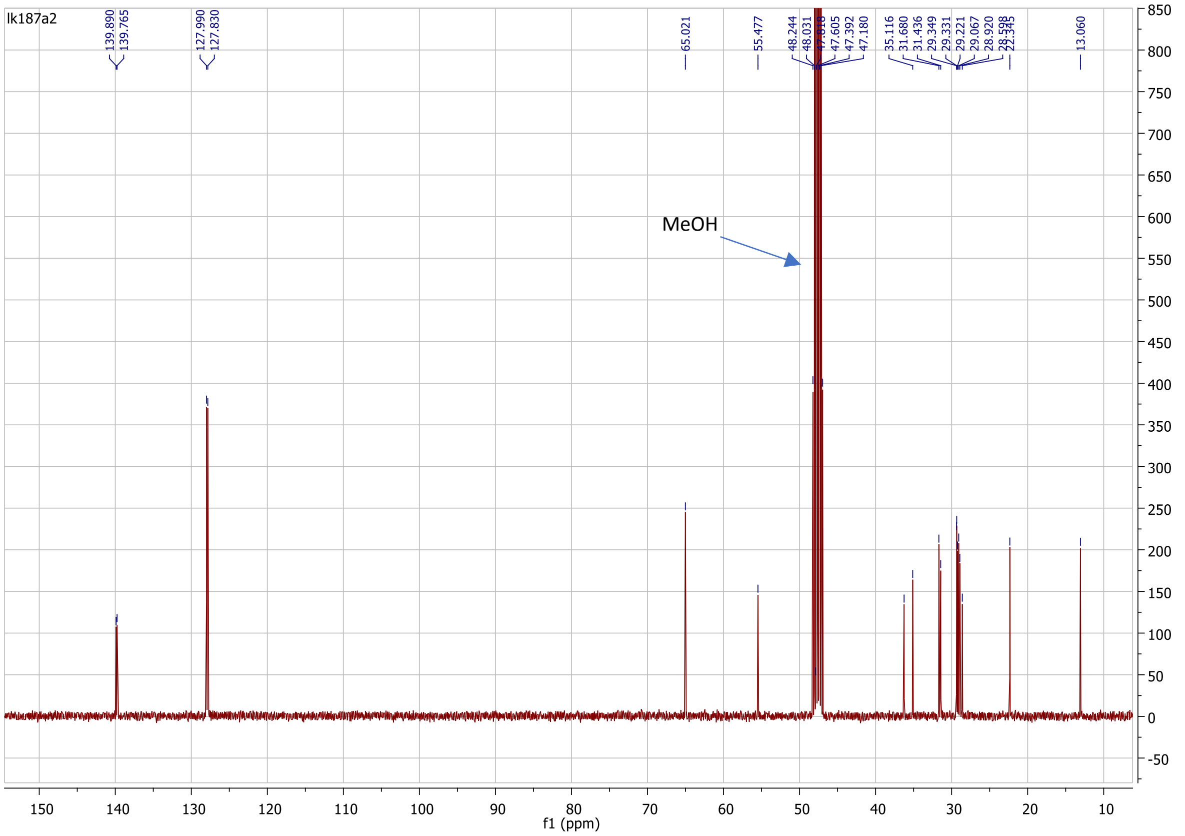

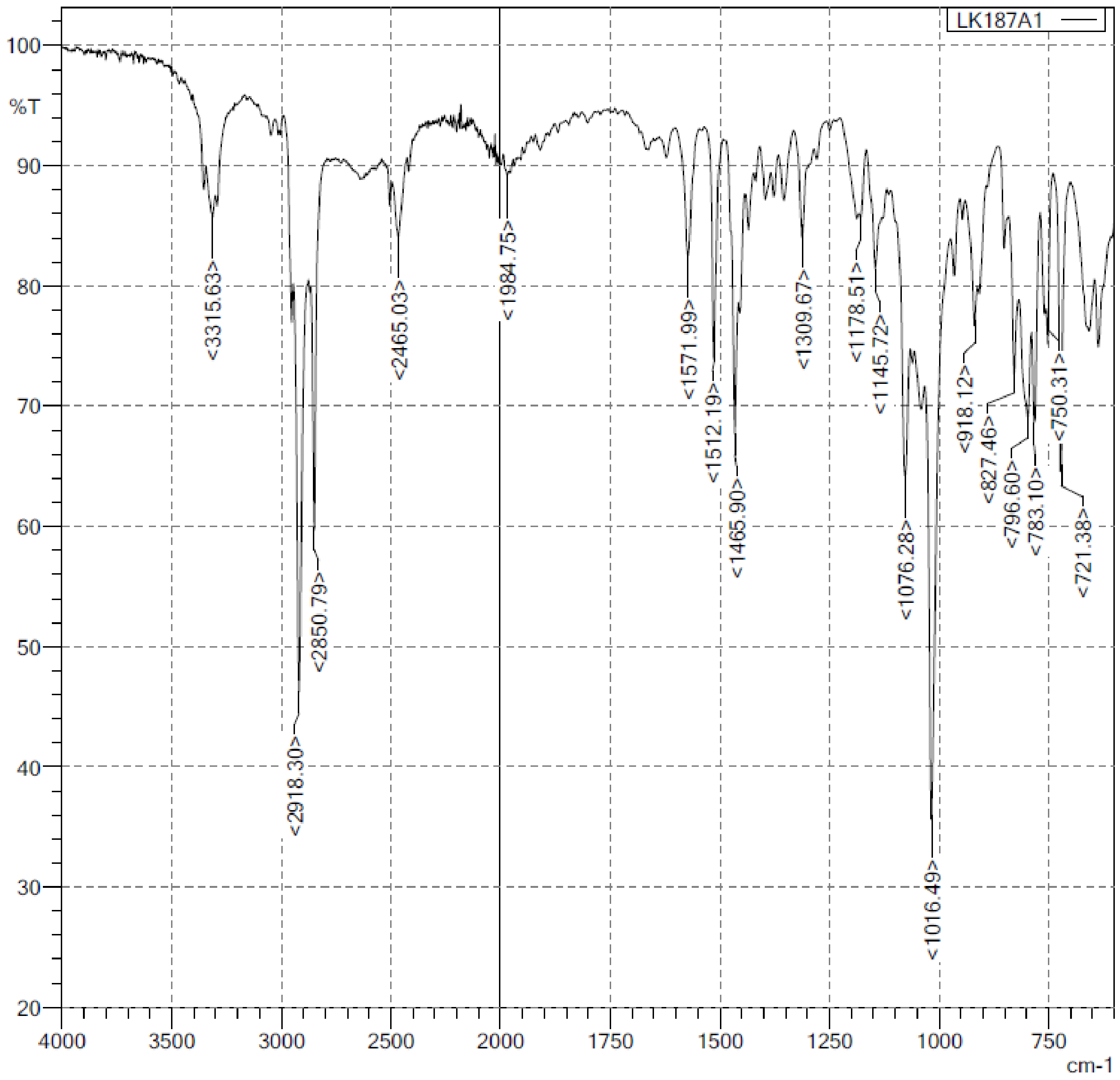
LK187A1
%T
100
90
80
70
60
50
40
30
20
4000
3500
3000
2500
2000
1750
1500
1250
1000
750
cm-1
<3315.63>
<2918.30>
<2850.79>
<2465.03>
<1984.75>
<1571.99>
<1512.19>
<1465.90>
<1309.67>
<1178.51>
<1145.72>
<1076.28>
<1016.49>
<918.12>
<827.46>
<796.60>
<783.10>
<750.31>
<721.38>

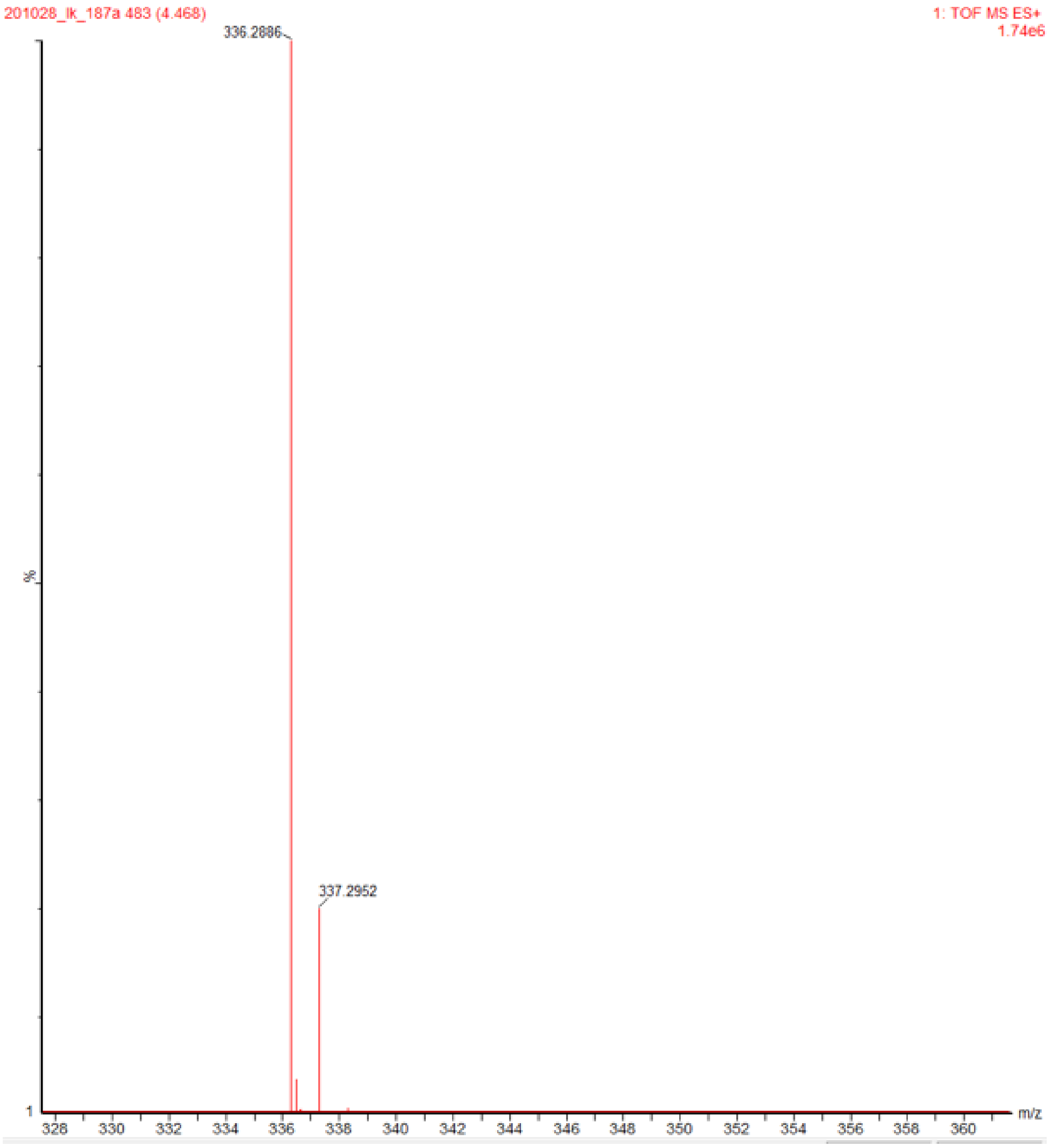
201028_lk_187a 483 (4.468)
1: TOF MS ES+
1.74e6
336.2886
337.2952
%
1
m/z
328
330
332
334
336
338
340
342
344
346
348
350
352
354
356
358
360

tert-butyl (2,2-dimethyl-5-(4-nitrostyryl)-1,3-dioxan-5-yl)carbamate **11**

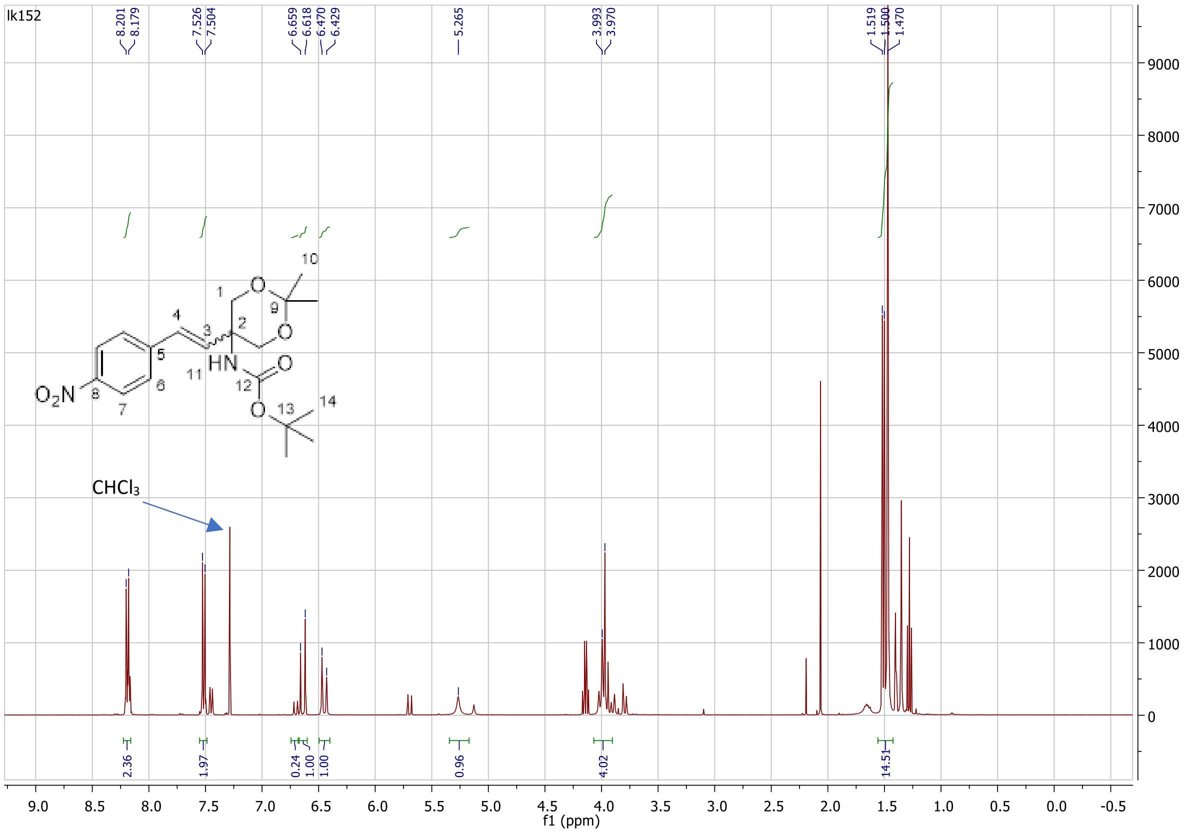

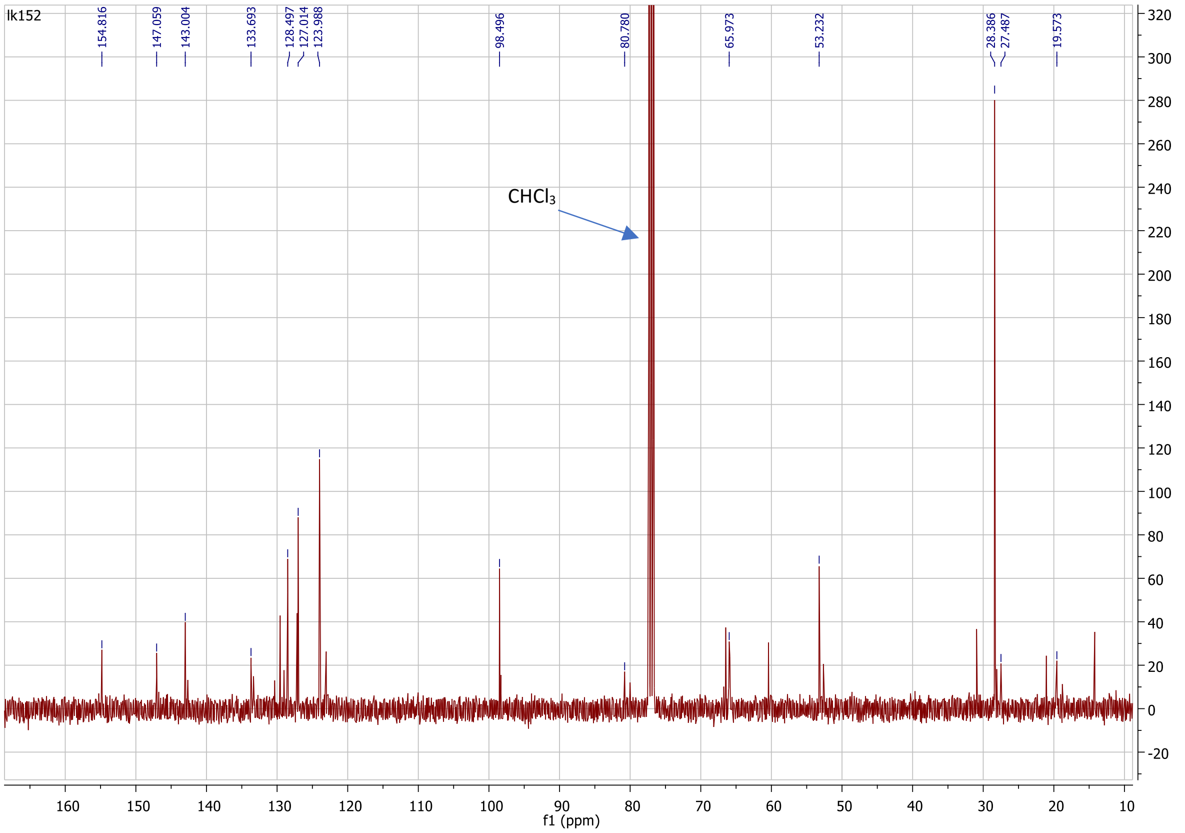

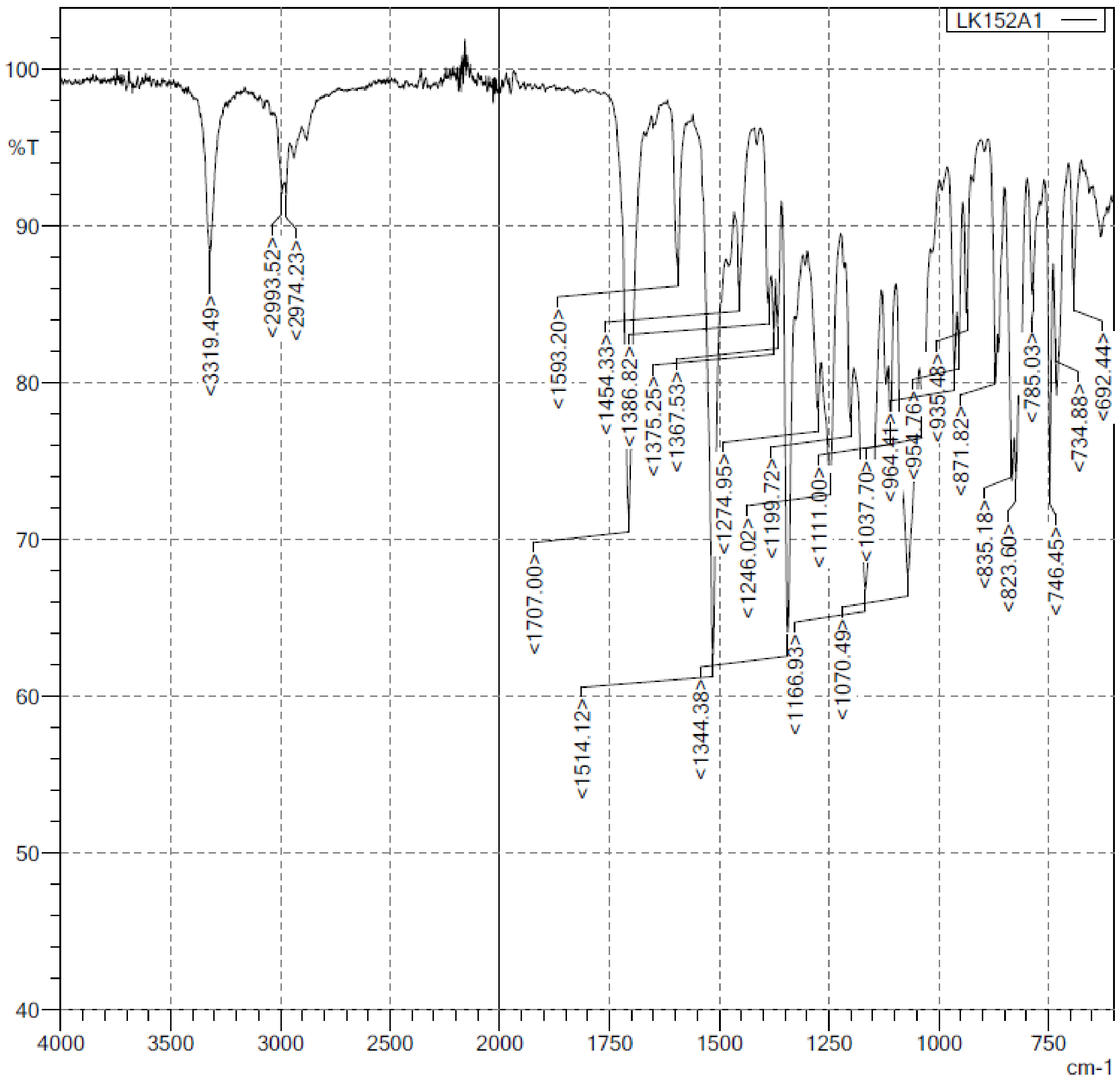
LK152A1
100
%T
90
80
70
60
50
40
4000
3500
3000
2500
2000
1750
1500
1250
1000
750
cm-1
3319.49
2993.52
2974.23
1707.00
1593.20
1514.12
1454.33
1386.82
1375.25
1367.53
1344.38
1274.95
1246.02
1199.72
1166.93
1111.00
1070.49
1037.70
964.41
954.76
935.48
871.82
835.18
823.60
785.03
746.45
734.88
692.44

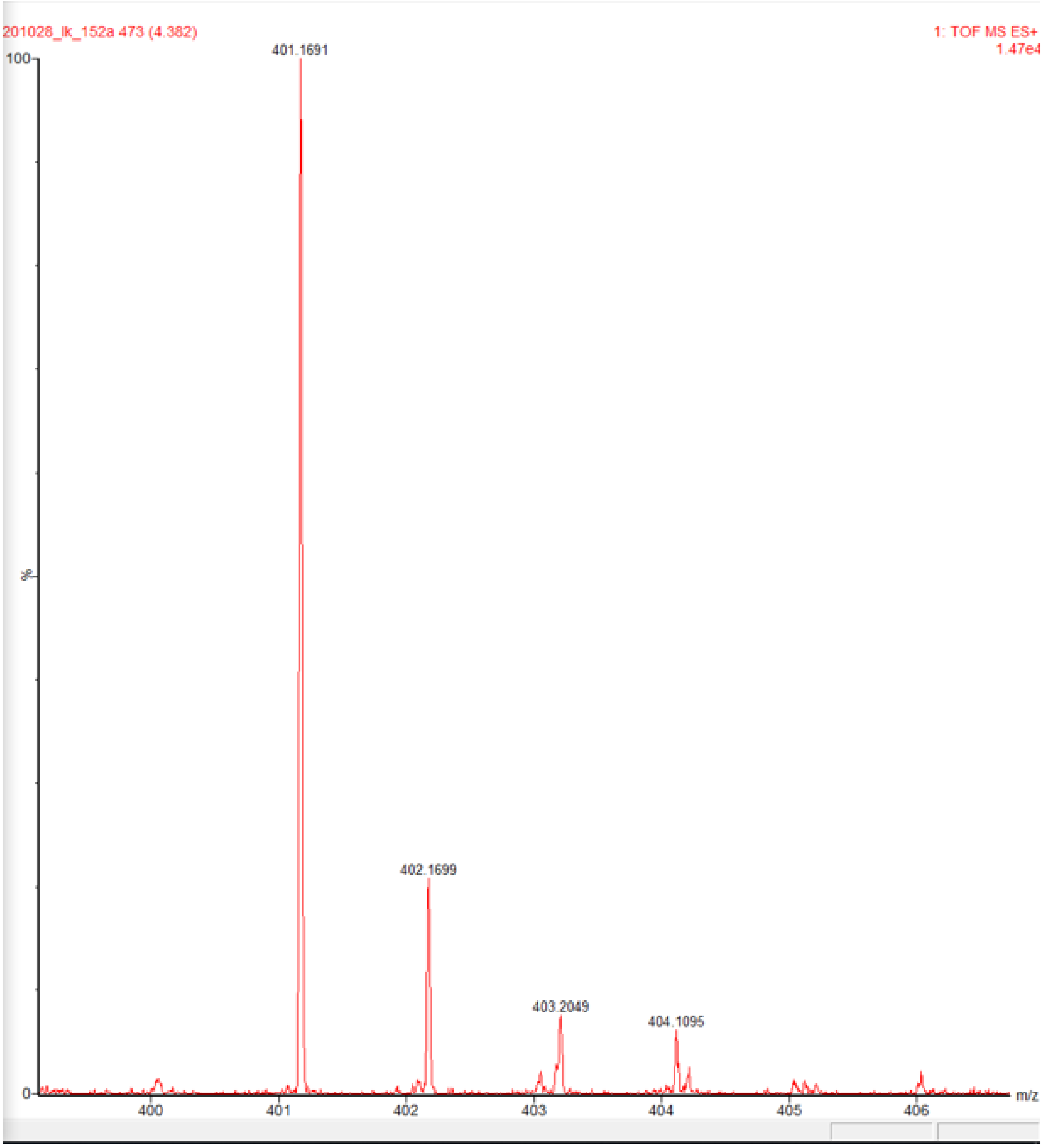

201028_lk_152a 473 (4.382)
1: TOF MS ES+
1.47e4
100
%
0
401.1691
402.1699
403.2049
404.1095
400
401
402
403
404
405
406
m/z

tert-butyl (5-(4-aminophenethyl)-2,2-dimethyl-1,3-dioxan-5-yl)carbamate **12**

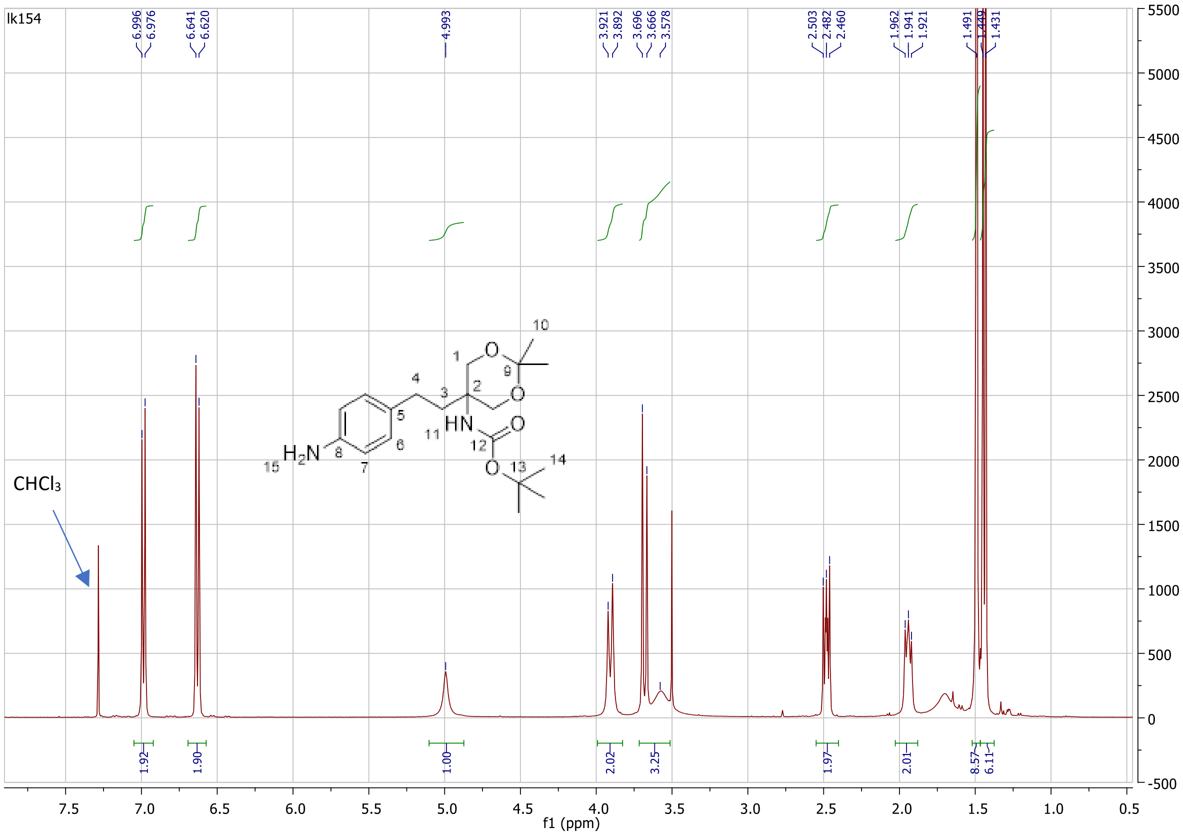

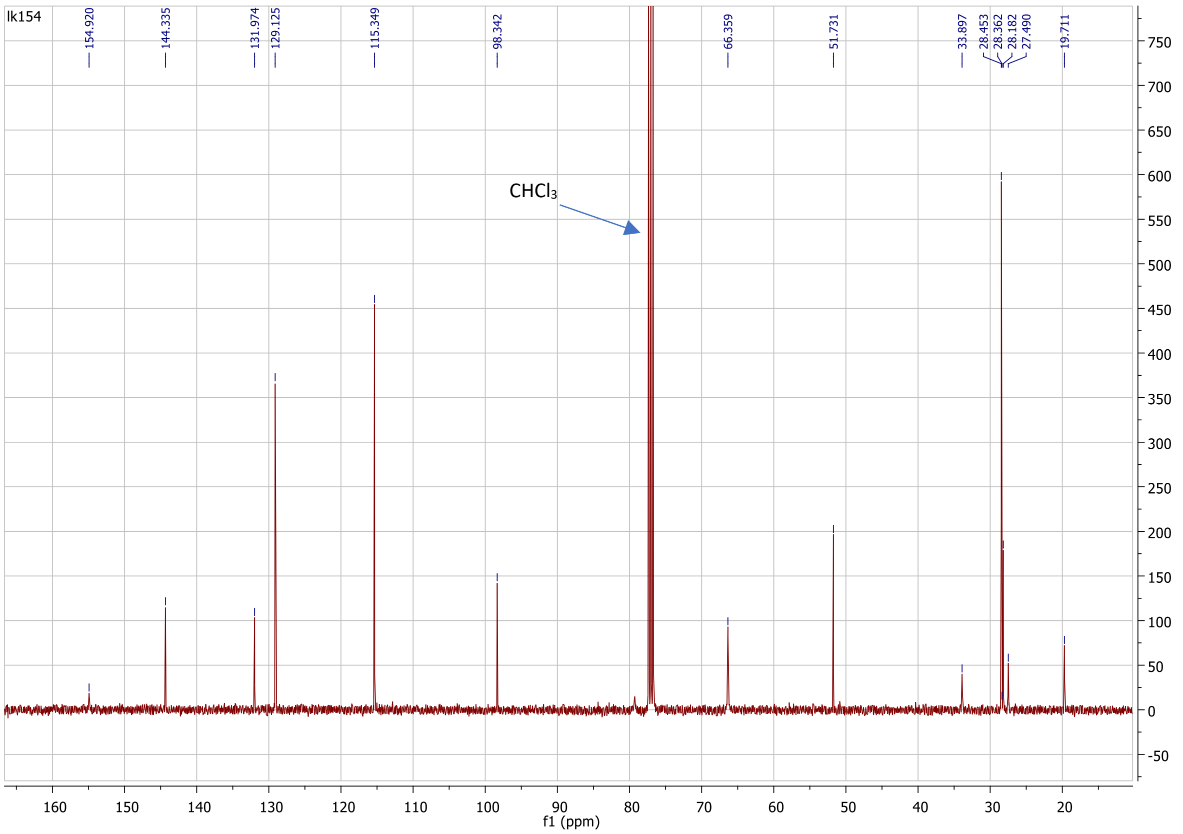

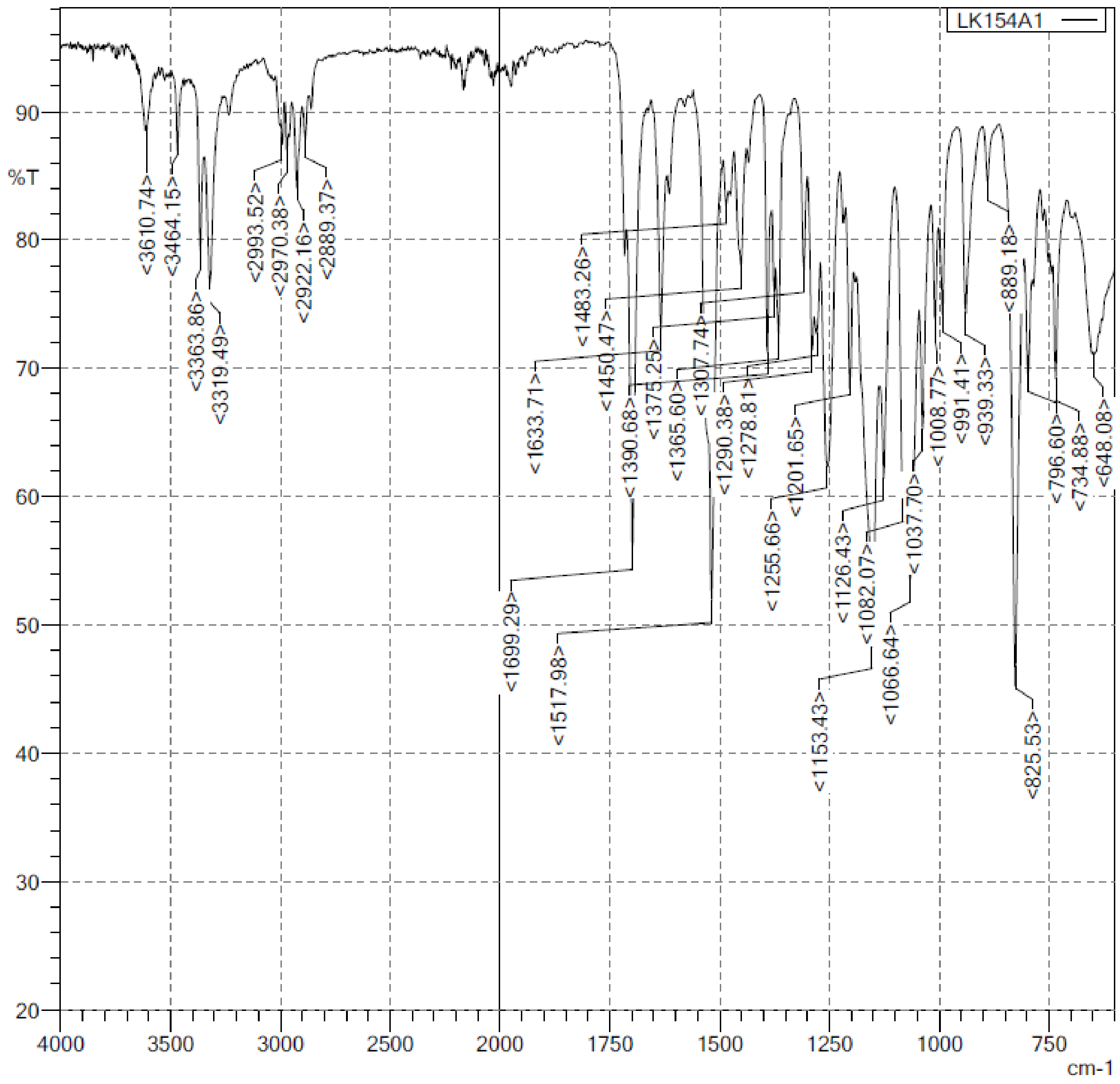

LK154A1
%T
cm-1
4000
3500
3000
2500
2000
1750
1500
1250
1000
750
20
30
40
50
60
70
80
90
<3610.74>
<3464.15>
<3363.86>
<3319.49>
<2993.52>
<2970.38>
<2922.16>
<2889.37>
<1699.29>
<1633.71>
<1517.98>
<1483.26>
<1450.47>
<1390.68>
<1375.25>
<1365.60>
<1307.74>
<1290.38>
<1278.81>
<1255.66>
<1201.65>
<1153.43>
<1126.43>
<1082.07>
<1066.64>
<1037.70>
<1008.77>
<991.41>
<939.33>
<889.18>
<825.53>
<796.60>
<734.88>
<648.08>

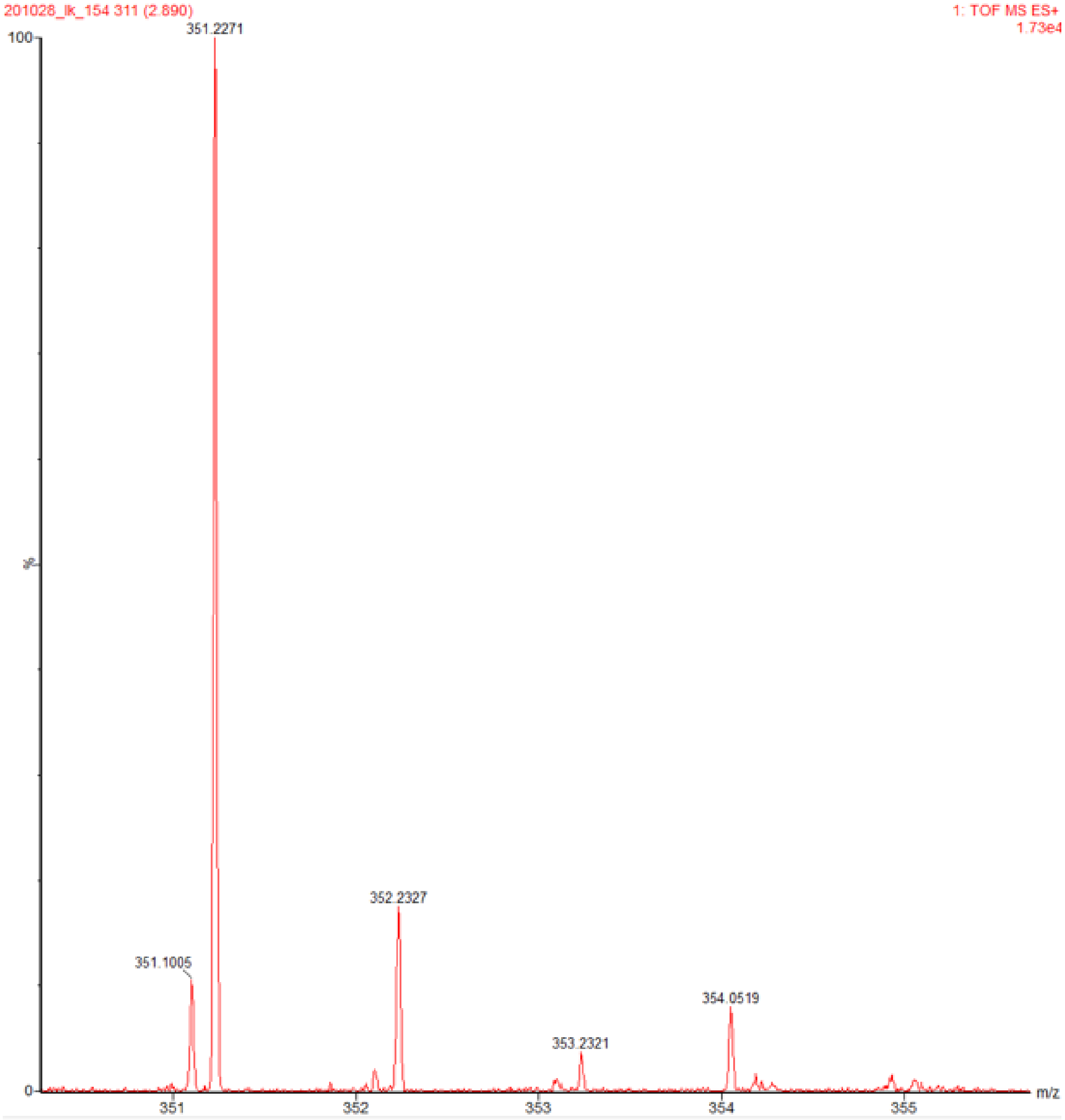

201028_lk_154 311 (2.890)
1: TOF MS ES+
1.73e4
351.2271
352.2327
351.1005
354.0519
353.2321
100
%
0
351
352
353
354
355
m/z

tert-butyl (2,2-dimethyl-5-(4-(oct-1-yn-1-yl)phenethyl)-1,3-dioxan-5-yl)carbamate **14**

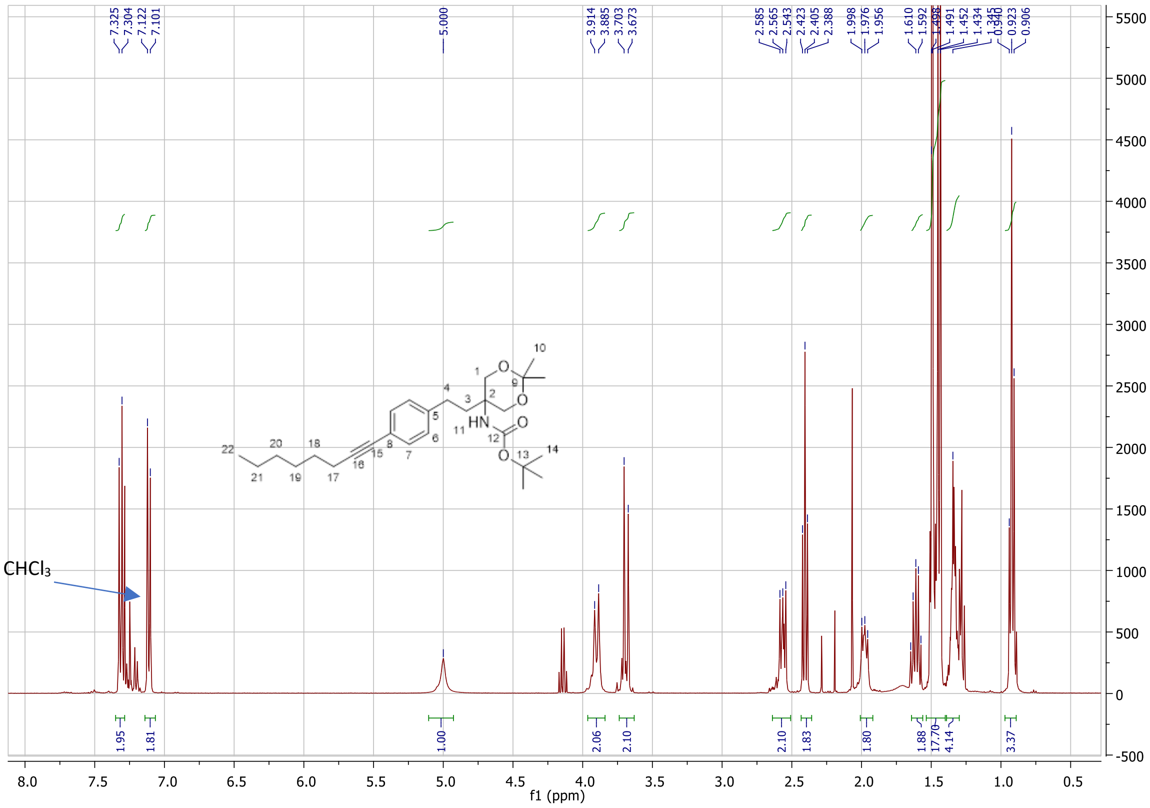

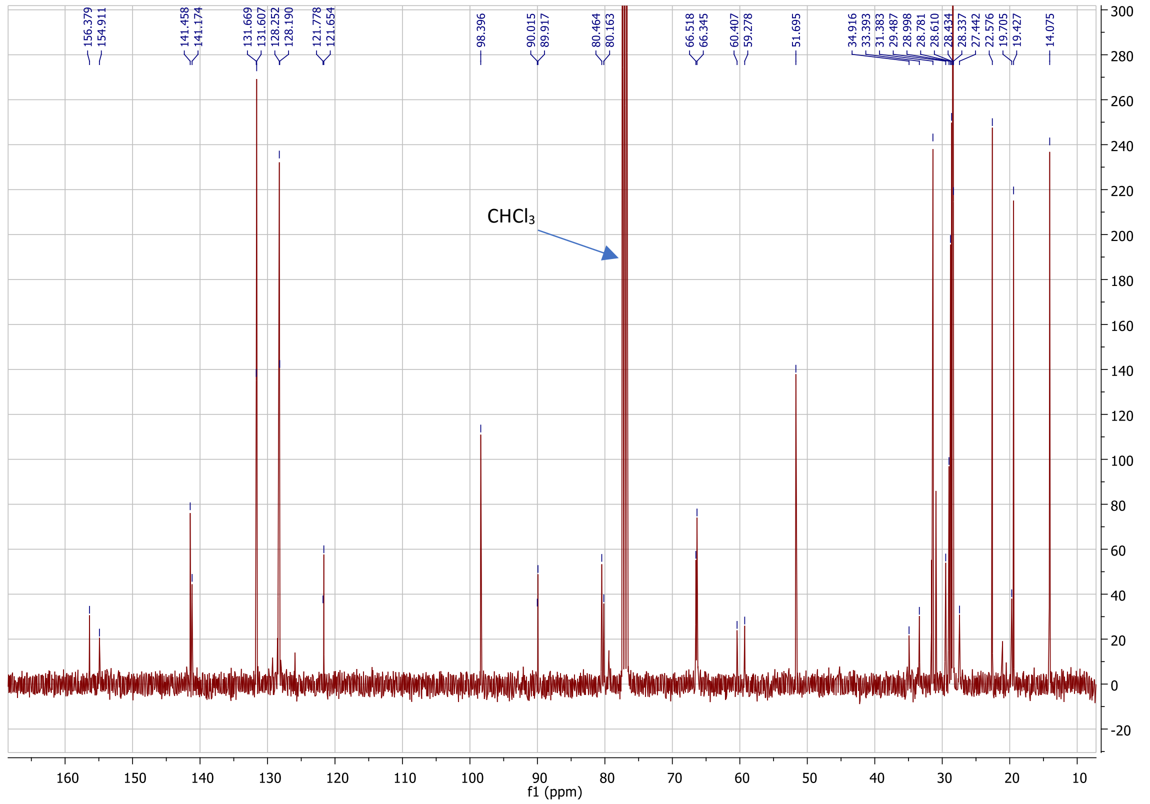

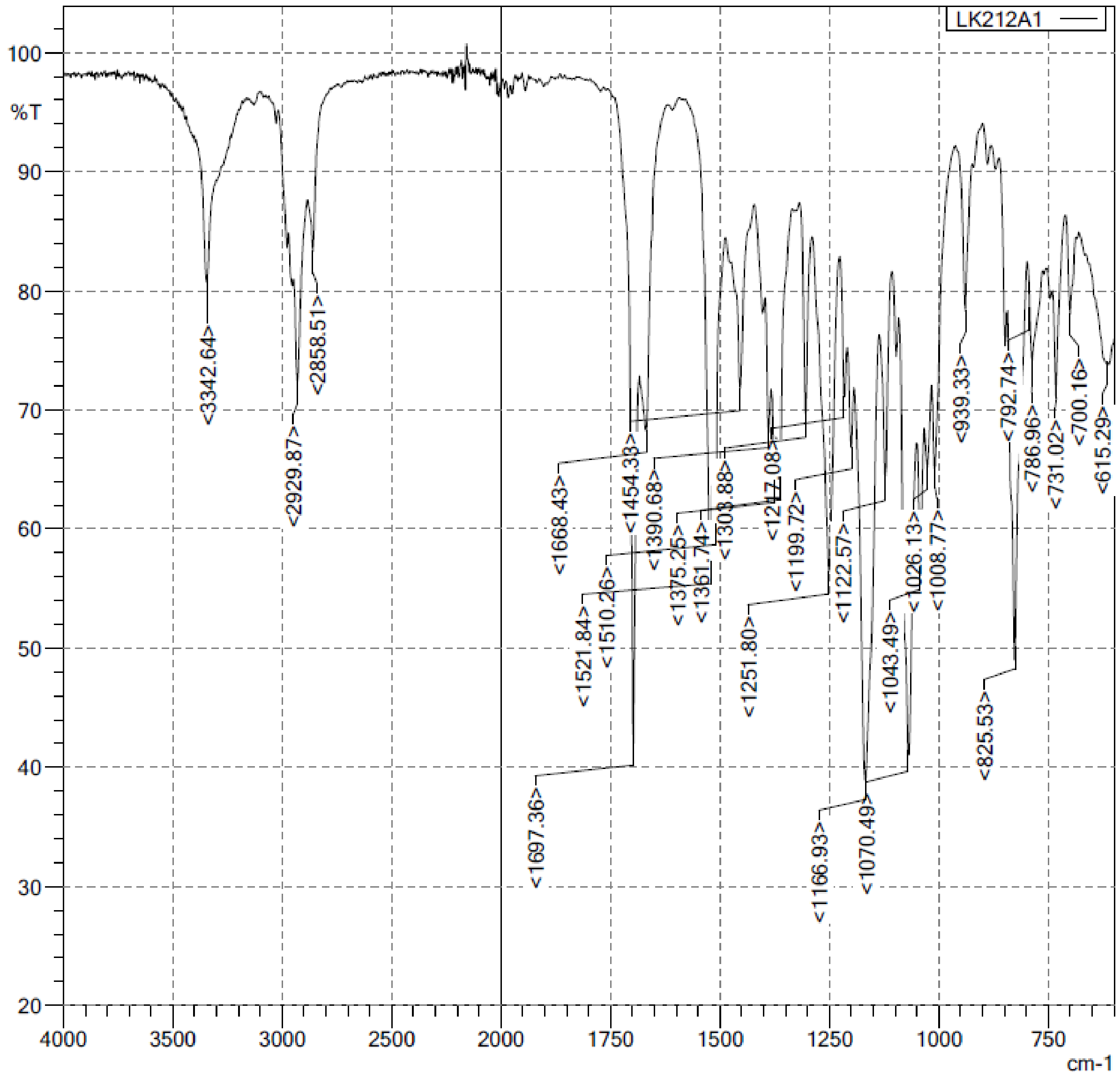
LK212A1
%T
cm-1
<3342.64>
<2929.87>
<2858.51>
<1697.36>
<1668.43>
<1521.84>
<1510.26>
<1454.33>
<1390.68>
<1375.25>
<1361.74>
<1303.88>
<1247.08>
<1251.80>
<1199.72>
<1166.93>
<1122.57>
<1070.49>
<1043.49>
<1026.13>
<1008.77>
<939.33>
<825.53>
<792.74>
<786.96>
<731.02>
<700.16>
<615.29>

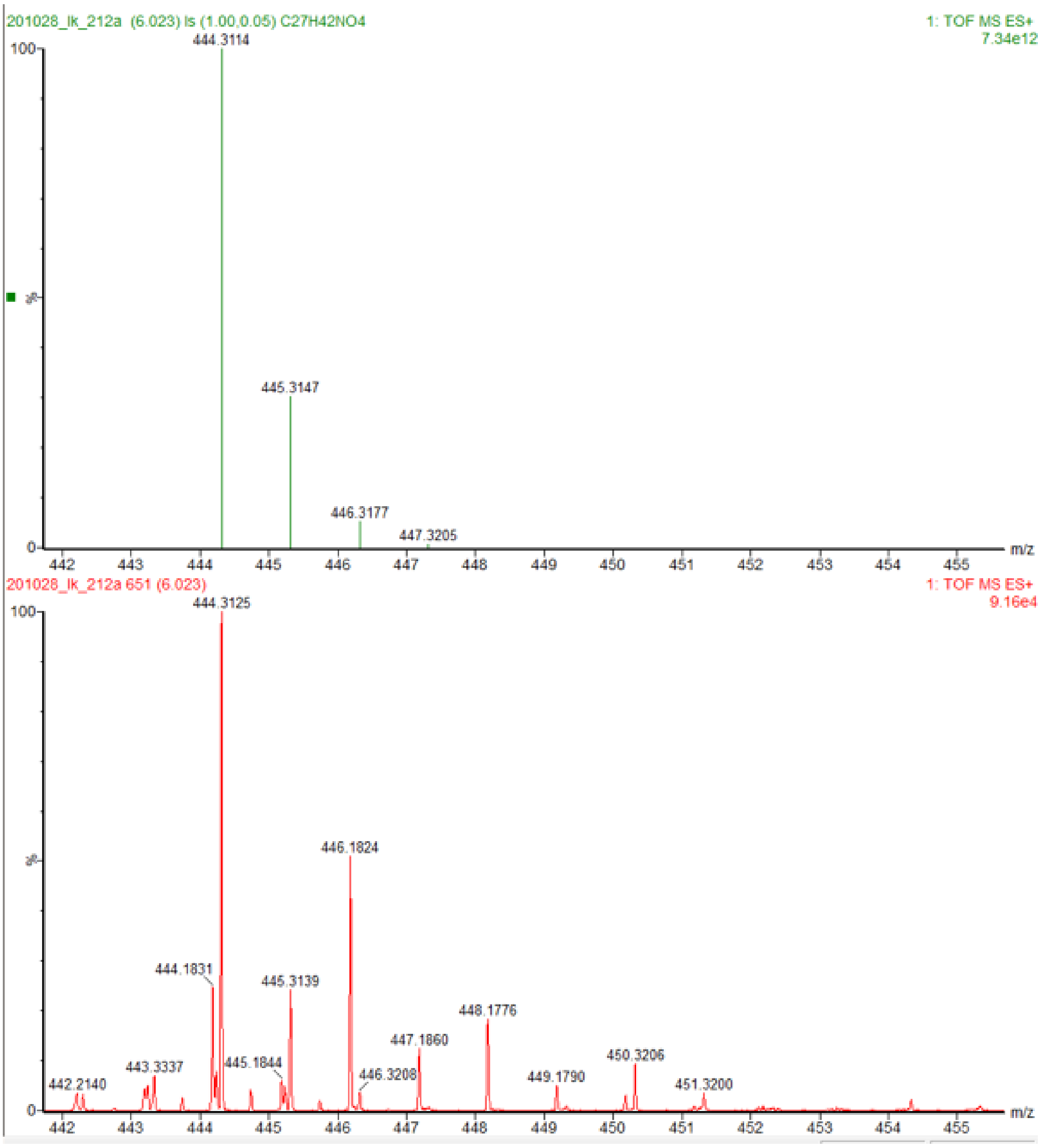

201028_lk_212a (6.023) Is (1.00,0.05) C27H42NO4
1: TOF MS ES+
7.34e12
100
%
0
444.3114
445.3147
446.3177
447.3205
442
443
444
445
446
447
448
449
450
451
452
453
454
455
m/z
201028_lk_212a 651 (6.023)
1: TOF MS ES+
9.16e4
444.3125
446.1824
444.1831
445.3139
448.1776
447.1860
450.3206
443.3337
445.1844
446.3208
449.1790
442.2140
451.3200

tert-butyl (2,2-dimethyl-5-(4-(Z-oct-1-ene-1-yl)phenethyl)-1,3-dioxan-5-yl)carbamate **15**

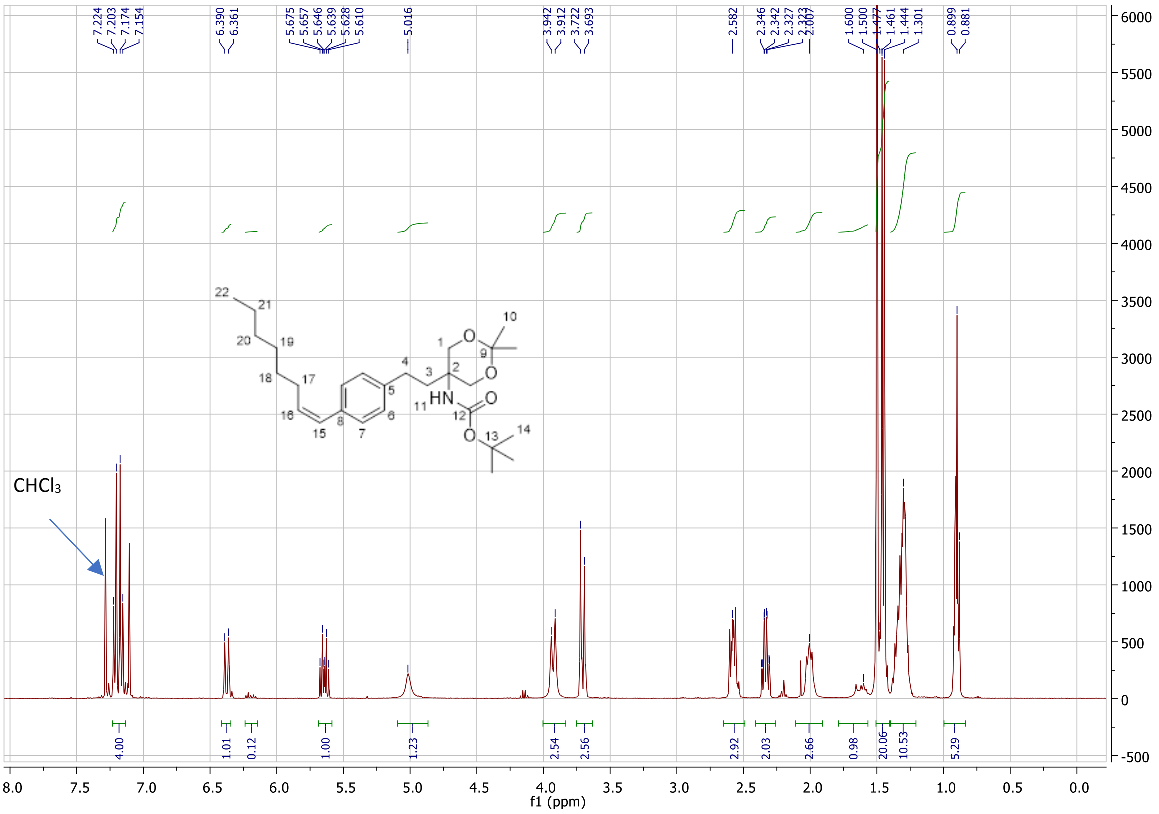

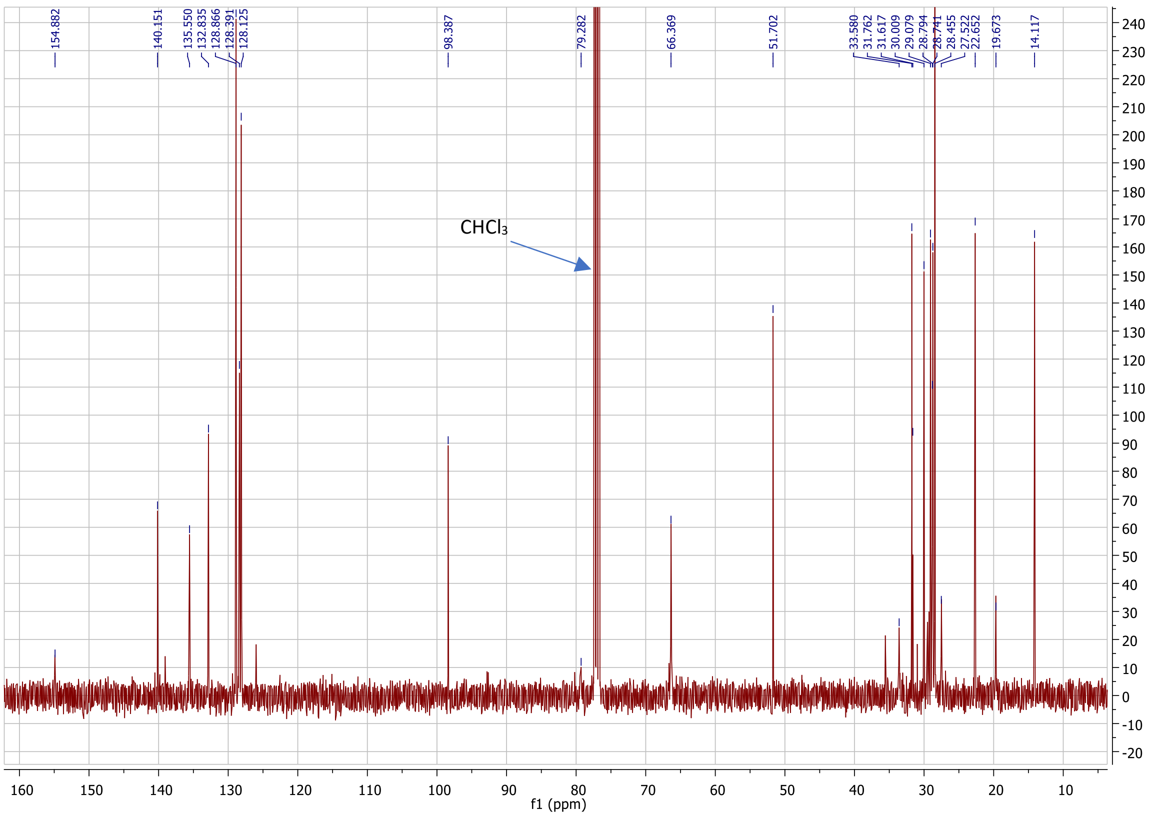

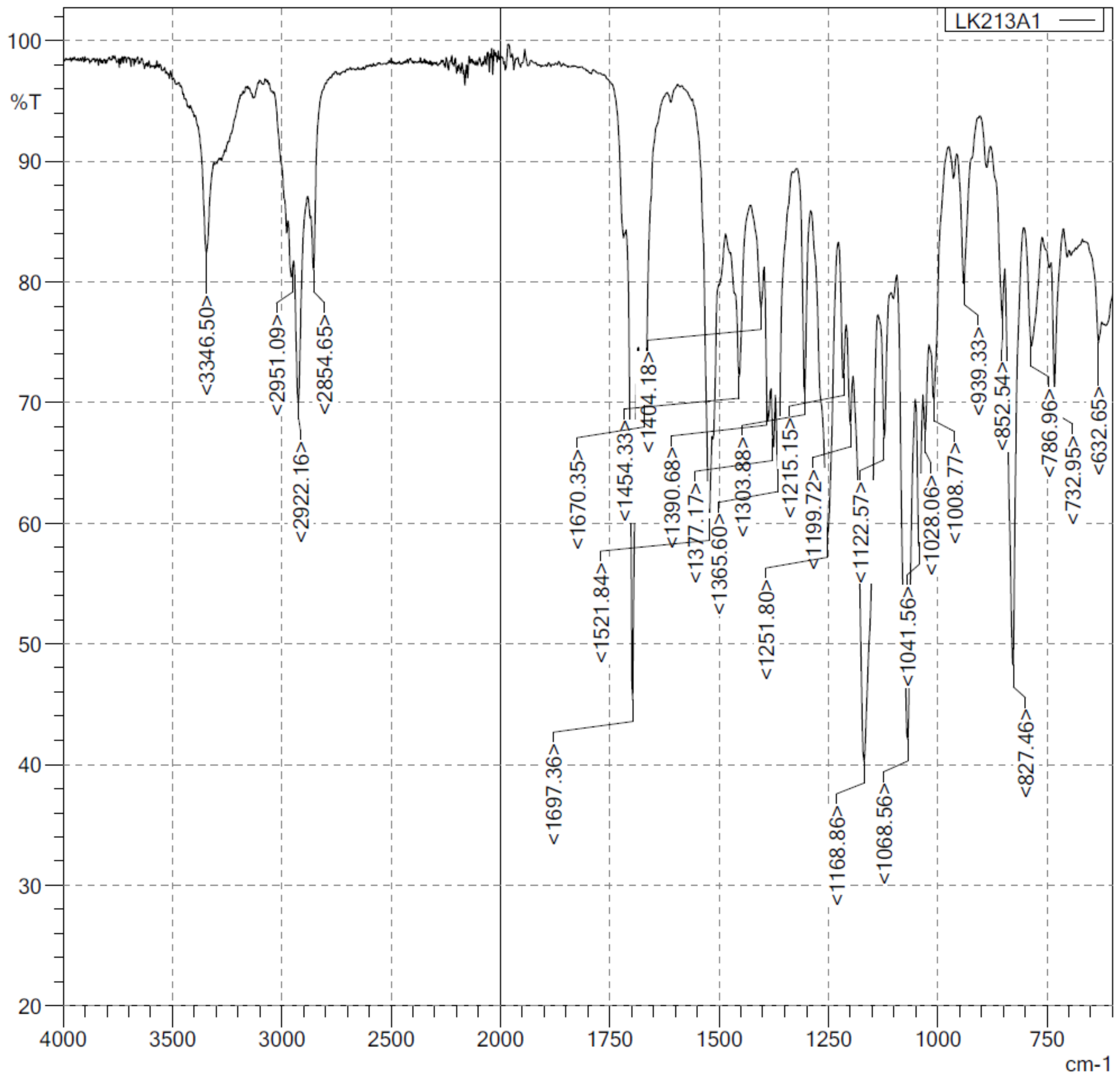
LK213A1
100
%T
90
80
70
60
50
40
30
20
4000
3500
3000
2500
2000
1750
1500
1250
1000
750
cm-1
<3346.50>
<2951.09>
<2922.16>
<2854.65>
<1697.36>
<1670.35>
<1521.84>
<1454.33>
<1404.18>
<1390.68>
<1377.17>
<1365.60>
<1303.88>
<1251.80>
<1215.15>
<1199.72>
<1168.86>
<1122.57>
<1068.56>
<1041.56>
<1028.06>
<1008.77>
<939.33>
<852.54>
<827.46>
<786.96>
<732.95>
<632.65>

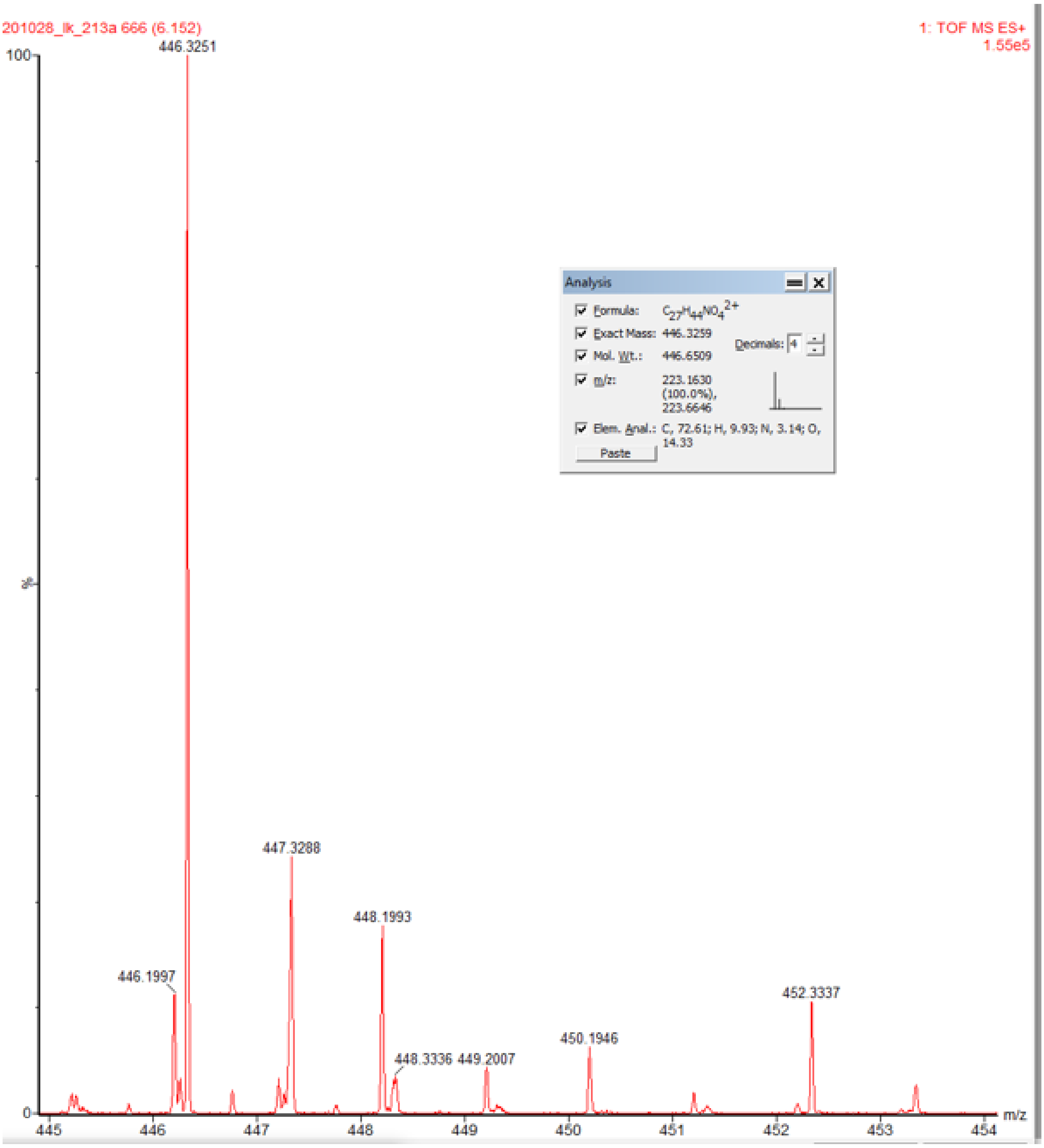
201028_lk_213a 666 (6.152)
1: TOF MS ES+
1.55e5
100
%
0
446.3251
447.3288
448.1993
446.1997
452.3337
450.1946
448.3336 449.2007
445
446
447
448
449
450
451
452
453
454
m/z
Analysis
Formula: C27H44NO4 2+
Exact Mass: 446.3259
Mol. Wt.: 446.6509
Decimals: 4
m/z: 223.1630 (100.0%), 223.6646
Elem. Anal.: C, 72.61; H, 9.93; N, 3.14; O, 14.33
Paste

2-amino-2-(4-(Z-oct-1-en-1-yl)phenethyl)propane-1,3-diol **16**

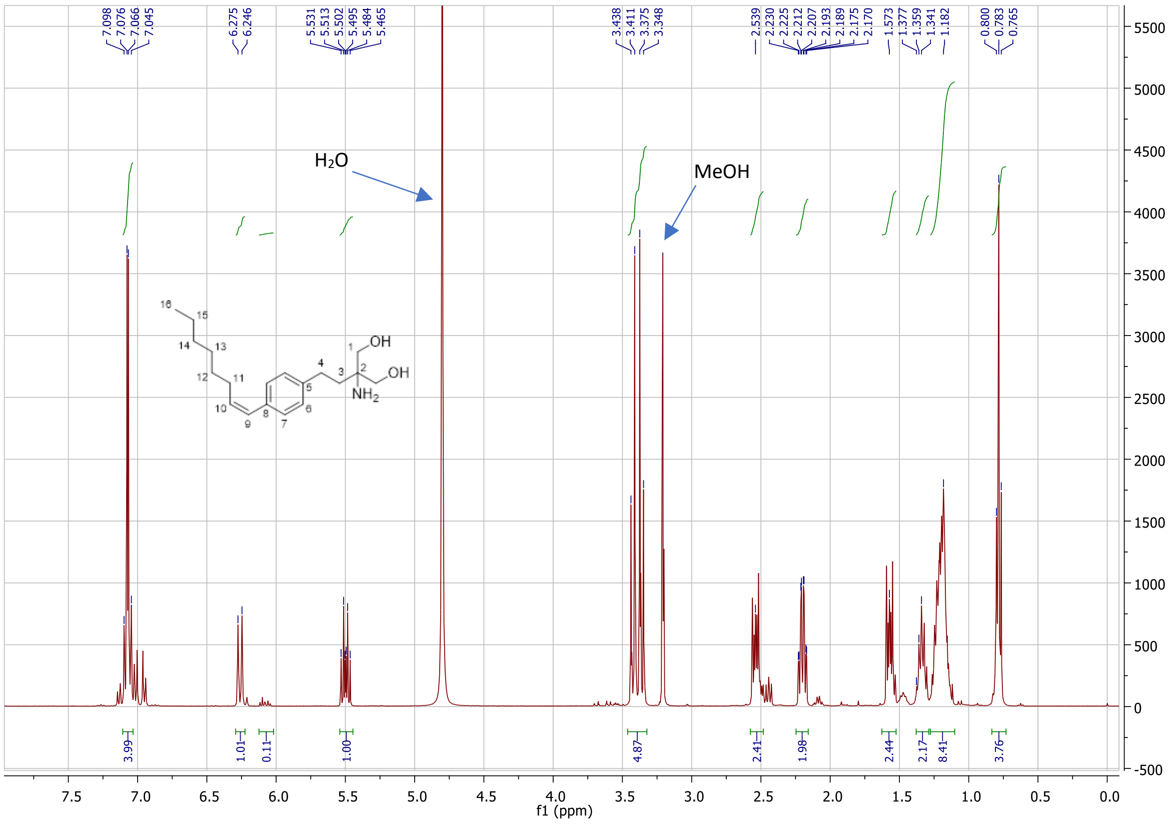

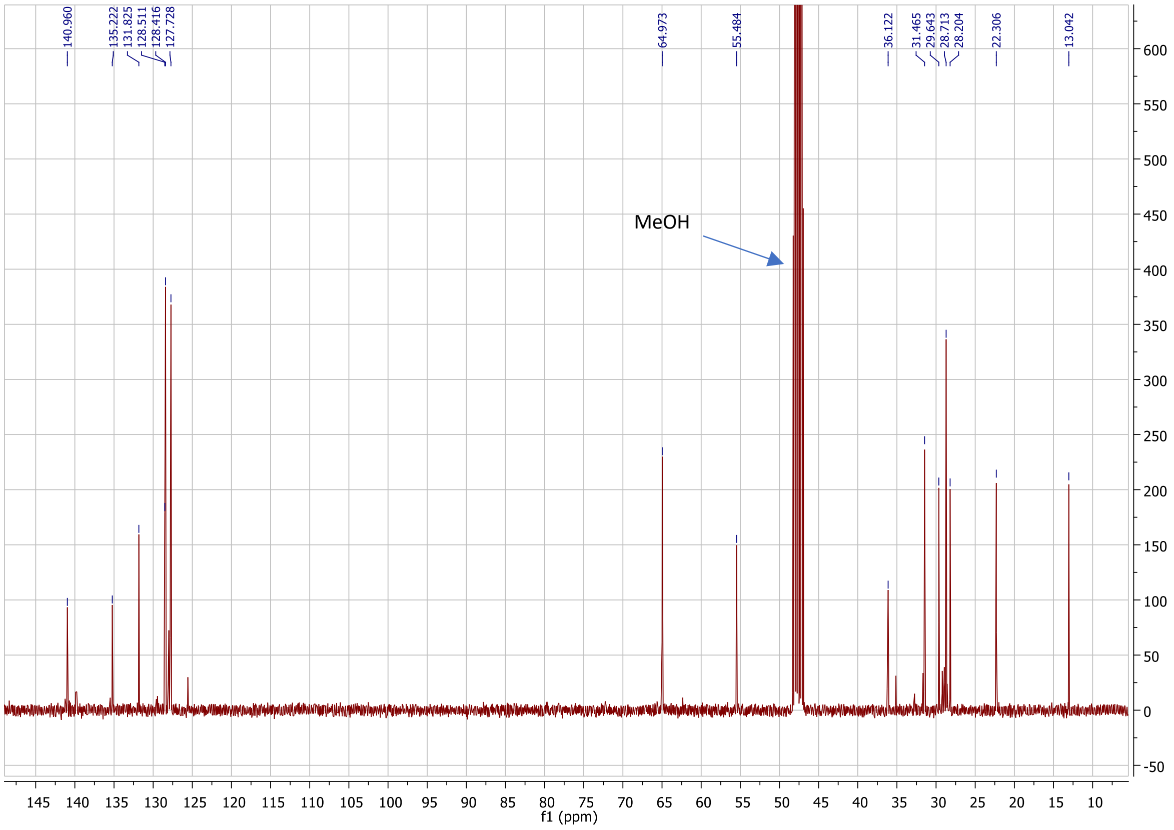

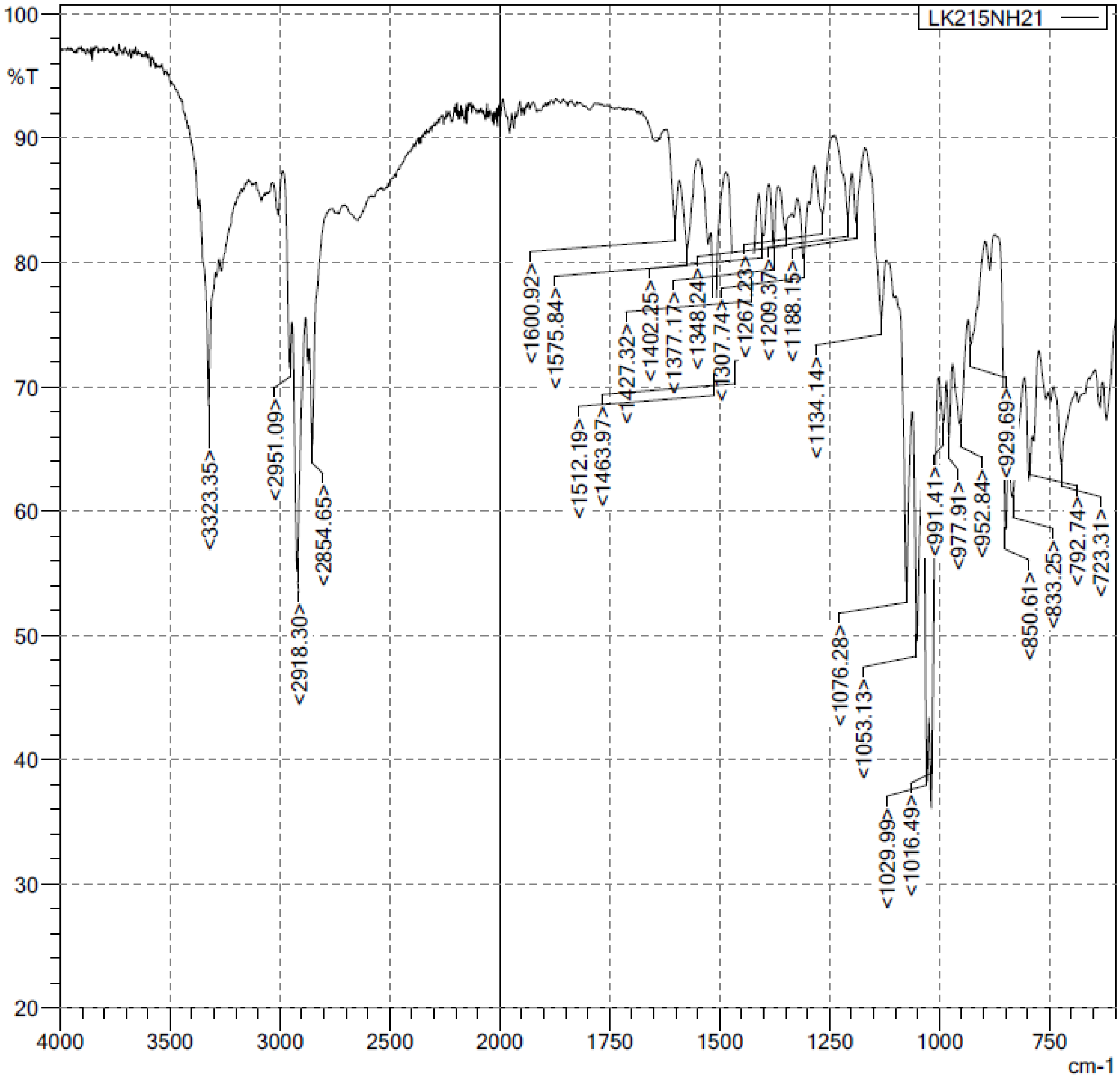
LK215NH21
%T
100
90
80
70
60
50
40
30
20
4000
3500
3000
2500
2000
1750
1500
1250
1000
750
cm-1
<3323.35>
<2951.09>
<2918.30>
<2854.65>
<1600.92>
<1575.84>
<1512.19>
<1463.97>
<1427.32>
<1402.25>
<1377.17>
<1348.24>
<1307.74>
<1267.23>
<1209.37>
<1188.15>
<1134.14>
<1076.28>
<1053.13>
<1029.99>
<1016.49>
<991.41>
<977.91>
<952.84>
<929.69>
<850.61>
<833.25>
<792.74>
<723.31>

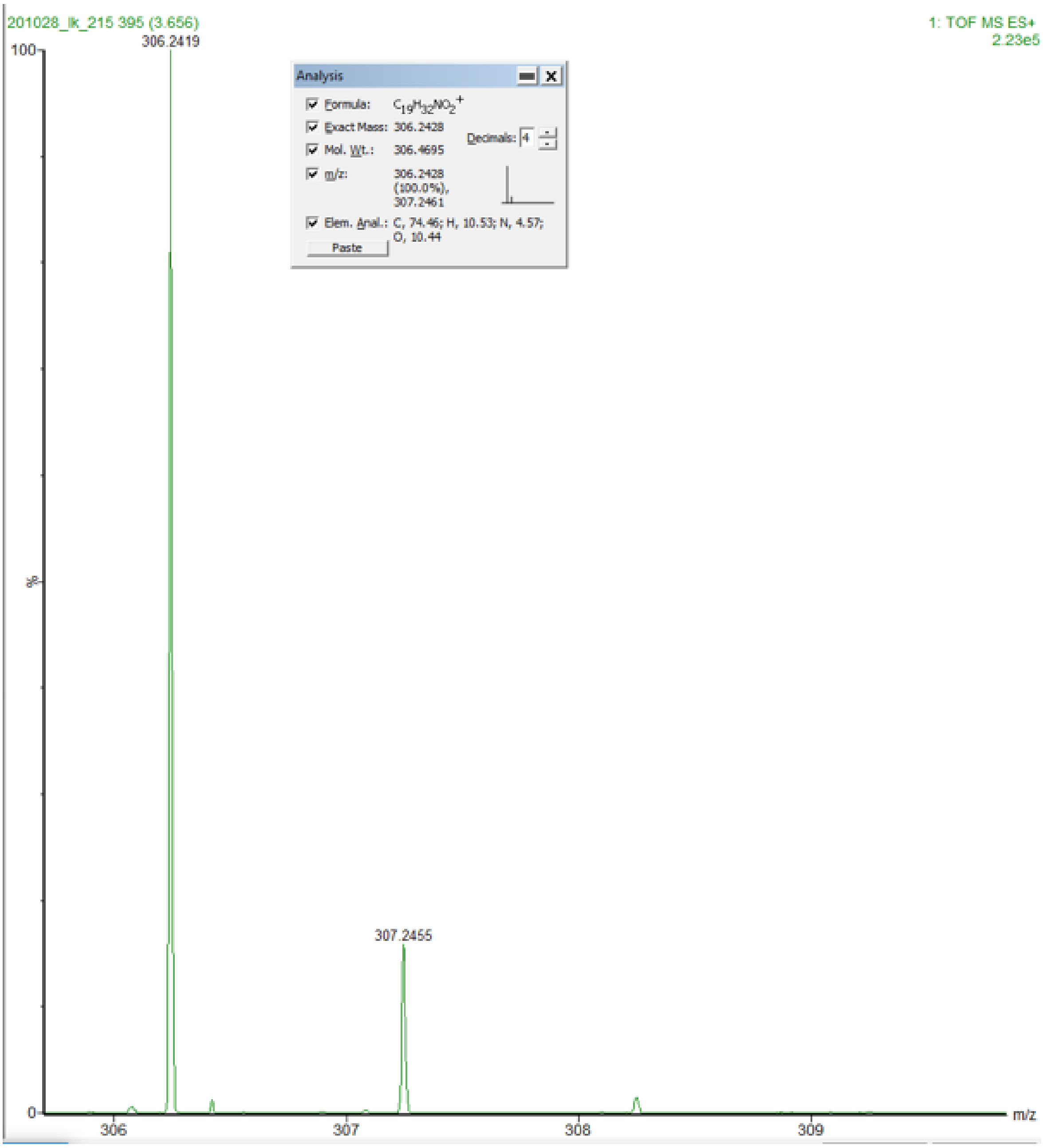

201028_lk_215 395 (3.656)
1: TOF MS ES+
2.23e5
306.2419
307.2455
100
%
0
306
307
308
309
m/z
Analysis
Formula: $C_{19}H_{32}NO_2^+$
Exact Mass: 306.2428
Mol. Wt.: 306.4695
m/z: 306.2428 (100.0%), 307.2461
Decimals: 4
Elem. Anal.: C, 74.46; H, 10.53; N, 4.57; O, 10.44
Paste

Supporting Information References

(1) Shaikh, R. S.; Schilson, S. S.; Wagner, S.; Hermann, S.; Keul, P.; Levkau, B.; Schäfers, M.; Haufe, G. Synthesis and Evaluation of Fluorinated Fingolimod (FTY720) Analogues for Sphingosine-1-Phosphate Receptor Molecular Imaging by Positron Emission Tomography. *J. Med. Chem.* **2015**, *58* (8), 3471–3484. https://doi.org/10.1021/jm502021d.

(2) Dong, Q.; Li, G.; Wang, H.; Wing-Tat Pong, P.; Leung, C. W.; Manners, I.; Ho, C. L.; Li, H.; Wong, W. Y. Investigation of Pyrolysis Temperature in the One-Step Synthesis of L10 FePt Nanoparticles from a FePt-Containing Metallopolymer. *J. Mater. Chem. C* **2015**, *3* (4), 734–741. https://doi.org/10.1039/c4tc02058h.

(3) Gao, Y.; Yang, C.; Bai, S.; Liu, X.; Wu, Q.; Wang, J.; Jiang, C.; Qi, X. Visible-Light-Induced Nickel-Catalyzed Cross-Coupling with Alkylzirconocenes from Unactivated Alkenes. *Chem* **2020**, *6* (3), 675–688. https://doi.org/10.1016/j.chempr.2019.12.010.

(4) Kiuchi, M.; Adachi, K.; Kohara, T.; Minoguchi, M.; Hanano, T.; Aoki, Y.; Mishina, T.; Arita, M.; Nakao, N.; Ohtsuki, M.; Hoshino, Y.; Teshima, K.; Chiba, K.; Sasaki, S.; Fujita, T. Synthesis and Immunosuppressive Activity of 2-Substituted 2- Aminopropane-1,3-Diols and 2-Aminoethanols. *J. Med. Chem.* **2000**, *43* (15), 2946–2961. https://doi.org/10.1021/jm000173z.

(5) Radl, S.; Doubsky, J. A Method for the Preparation of Fingolimod. WO 2013/185740 A1, 2013.

(6) Mei, T. W.; Luo, Y.; Feng, X. J.; Lu, W.; Yang, B. Suzuki Coupling Based Synthesis and in Vitro Cytotoxic Evaluation of Fingolimod and Analogues. *Tetrahedron* **2013**, *69* (14), 2927–2932. https://doi.org/10.1016/j.tet.2013.02.030.
